# NMR Observation of Sulfhydryl Signals in SARS‐CoV‐2 Main Protease Aids Structural Studies

**DOI:** 10.1002/cbic.202200471

**Published:** 2022-09-07

**Authors:** Angus J. Robertson, Jinfa Ying, Ad Bax

**Affiliations:** ^1^ Laboratory of Chemical Physics National Institute of Diabetes and Digestive and Kidney Disease National Institutes of Health Bethesda Maryland 20892 USA; ^2^ Biophysical Chemistry Center for Molecular Protein Science Lund University 22100 Lund Sweden

**Keywords:** AlphaFold-Multimers, ligand binding, NMR spectroscopy, M^pro^/3CLpro, SARS-CoV-2

## Abstract

The 68‐kDa homodimeric 3C‐like protease of SARS‐CoV‐2, M^pro^ (3CL^pro^/Nsp5), is a key antiviral drug target. NMR spectroscopy of this large system proved challenging and resonance assignments have remained incomplete. Here we present the near‐complete (>97 %) backbone assignments of a C145A variant of M^pro^ (M^pro^_C145A_) both with, and without, the N‐terminal auto‐cleavage substrate sequence, in its native homodimeric state. We also present SILLY (Selective Inversion of thioL and Ligand for NOESY), a simple yet effective pseudo‐3D NMR experiment that utilizes NOEs to identify interactions between Cys‐thiol or aliphatic protons, and their spatially proximate backbone amides in a perdeuterated protein background. High protection against hydrogen exchange is observed for 10 of the 11 thiol groups in M^pro^_C145A_, even those that are partially accessible to solvent. A combination of SILLY methods and high‐resolution triple‐resonance NMR experiments reveals site‐specific interactions between M^pro^, its substrate peptides, and other ligands, which present opportunities for competitive binding studies in future drug design efforts.

## Graphical abstract

**Sulfhydryl signals** were shown to be readily observable in nuclear Overhauser enhancement spectra of perdeuterated proteins, even for partially solvent‐accessible thiol groups. For dimeric SARS‐CoV‐2 main protease, these signals proved invaluable both for achieving nearly complete NMR backbone assignment in both apo‐ and substrate‐bound states and for validating structural features.

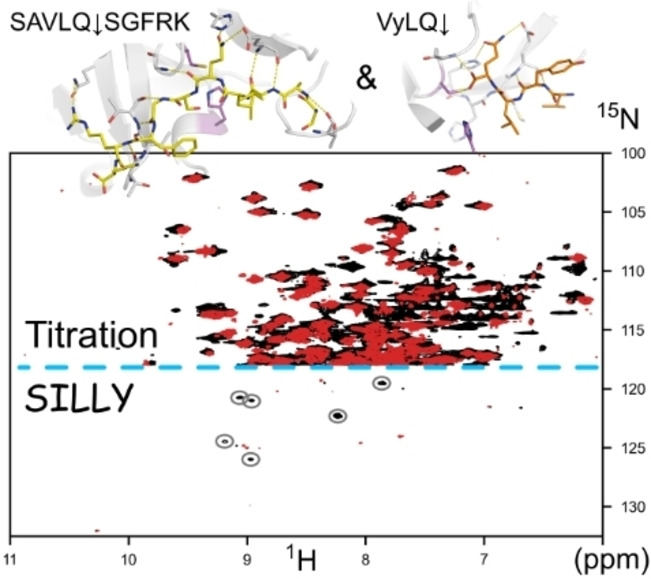

## Introduction

Thiol (‐SH) groups of Cys residues in proteins can play important structural and functional roles but are often omitted from NMR studies because the proton lacks large J couplings to stable spin‐1/2 isotopes that are required to record triple‐resonance NMR spectra. We demonstrate that the uniquely upfield thiol chemical shifts, combined with considerable protection from solvent exchange, make these signals readily observable in a perdeuterated protein, where they provide valuable information for validating both assignments and structural models. The approach is illustrated for the main protease of the SARS‐CoV‐2 virus, termed M^pro^, 3CL^pro^ or Nsp5, a chymotrypsin‐like cysteine protease that plays an essential role in the viral replication process by excising 11 non‐structural proteins (Nsp's), including itself, from the virus’ two polyprotein chains. The H41‐C145 catalytic dyad cleaves at the recognition sequence LQ↓S, and following the success of protease inhibitors to fight other viral diseases such as AIDS and hepatitis‐C, the first FDA‐approved specific M^pro^ inhibitor is also showing high clinical effectiveness.^[1]^ M^pro^ is only catalytically active as a homodimer,^[2]^ with one solvent exposed active site (an oxyanion hole) per monomer. Inactivation of the monomeric enzyme is mediated by the conversion of a loop (S138‐C145) to a short 3_10_‐helix, which collapses the oxyanion hole,^[3]^ a feature that was shown to be reversible on re‐binding of substrate peptide with implications for M^pro^ liberation from the polypeptide chain.^[4]^ The recognition sequence of M^pro^ is nominally five residues, including four residues prior to the cleavage site (occupying subsites S4…S1) and the first residue past the cleavage site (P1’ in subsite S1’).^[5]^ The sequence of the SARS‐CoV‐2 variant of M^pro^ is 96 % identical to that of SARS‐CoV, with a high fitness cost to viral particles that develop M^pro^ inhibitor‐resistance.^[6]^ The recent myriad of structure determinations by X‐ray crystallography^[7]^ and corroboration by NMR^[8]^ indicate that none of the 12 conservative sequence differences significantly impact the backbone structure of M^pro^.

In a search for leads in M^pro^‐targeted drug development, a recent NMR study of M^pro^ identified 38 hits.^[8a]^ Resonance assignment of large homodimeric proteins such as M^pro^ proved challenging and prior backbone amide assignments were limited to ∼63 %.^[8a]^ To prevent auto‐proteolysis of the enzyme during prolonged exposure to the high concentrations (0.2–1.1 mM) and temperature (25–35 °C) needed for NMR measurements, we focus on two M^pro^ active site mutants, H41Q (M^pro^_H41Q_) and C145 A (M^pro^_C145A_). Furthermore, NMR study of this 68‐kDa homodimeric protein requires deuteration of the non‐exchangeable hydrogens in combination with TROSY‐based observation of the back‐exchanged amide protons.^[9]^ Here, we take advantage of the 11 M^pro^_C145A_ Cys sulfhydryl protons (^1^H^γ^) that also exchange with solvent, as well as M^pro^‐substrate interactions, to validate and extend the M^pro^ assignments to essential completeness (>97 %) and demonstrate that the interaction with its N‐terminal autocleavage substrate extends well beyond the canonical five substrate‐binding subsites S4–S1’.

## Results and Discussion

**Figure 1:**
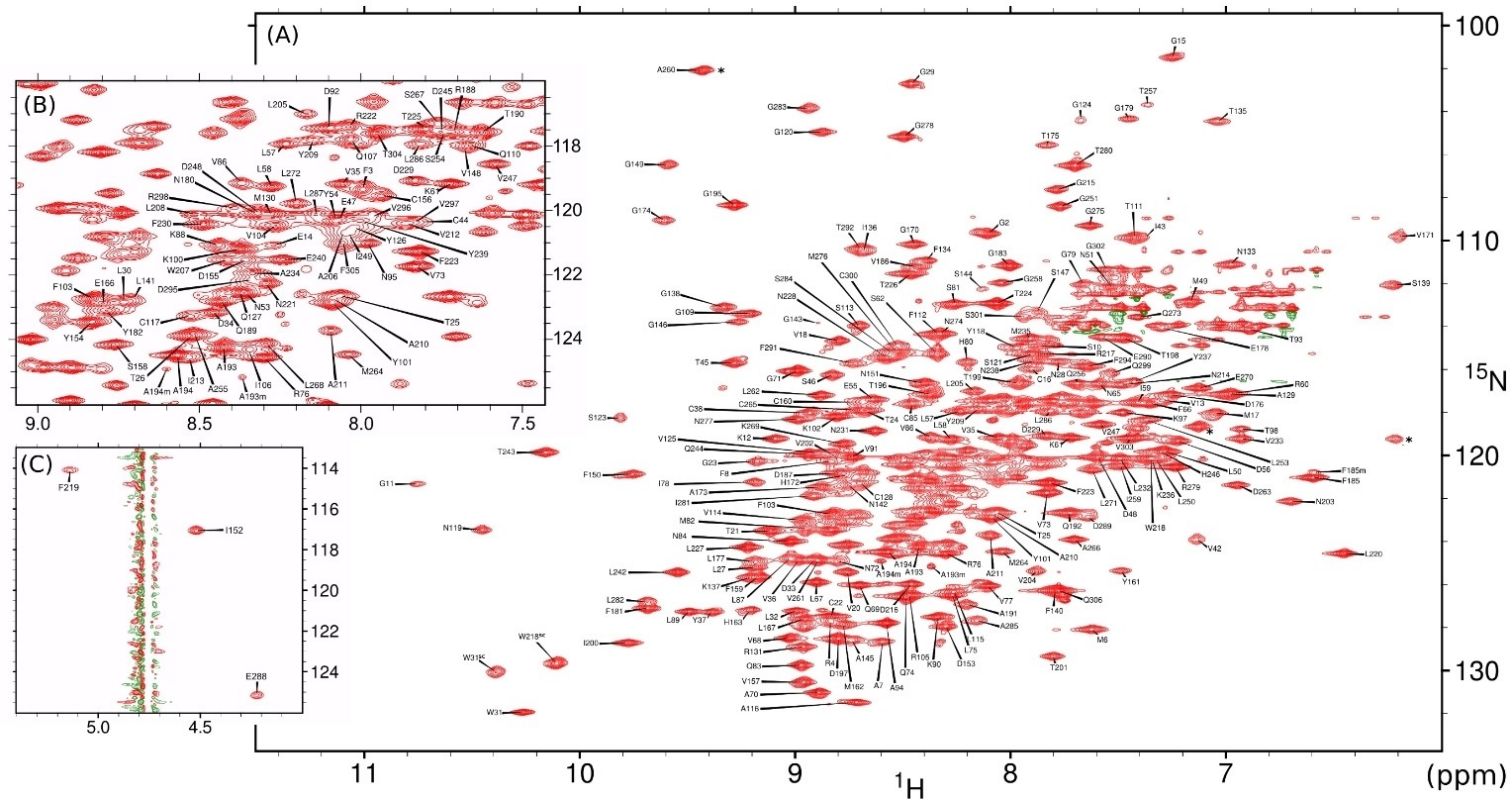
^1^H^15^N ‐TROSY‐HSQC spectrum (900 MHz, 25 °C) of reprotonated M^pro^_C145A_ in 10 mM sodium phosphate pH 7.0, supplemented with 3 % D_2_O and 0.5 mM tris(2‐carboxyethyl)phosphine hydrochloride (TCEP). (A) Full spectrum with aliased peaks marked with asterisks. (B) Expanded view of most crowded region of (A) with the contour threshold raised two‐fold for clarity, and (C) three residues near the H_2_O resonance, observed by omitting the typical water flip‐back pulses from the ^1^H^15^N‐TROSY‐HSQC pulse scheme.

The M^pro^ NMR assignment process proved considerably more challenging than expected (Figure 1; Supporting Information Experimental Section). Not only was a precarious partial‐refolding step necessary to fully re‐protonate the backbone amide groups after expression in D_2_O (Figure S1, see Supporting Information text), but resonance broadening from conformational and solvent exchange (particularly for residues near the active site; Figure S2,S3), as well as conformational heterogeneity involving the presence of both *cis* and *trans* isomers of P184 (Figure S4) posed major challenges to software^[10]^ that usually makes the resonance assignment step straightforward. Nevertheless, nearly complete backbone assignments of M^pro^_C145A_, M^pro^_H41Q_, and the complex with its N‐terminal autocleavage substrate analogue, M^pro^_C145A_:SAVLQSGFRK, were achieved (BMRB: 51455, 51456, Tables S1–S4, Supporting Information Figures S5–S7).

**Figure 2:**
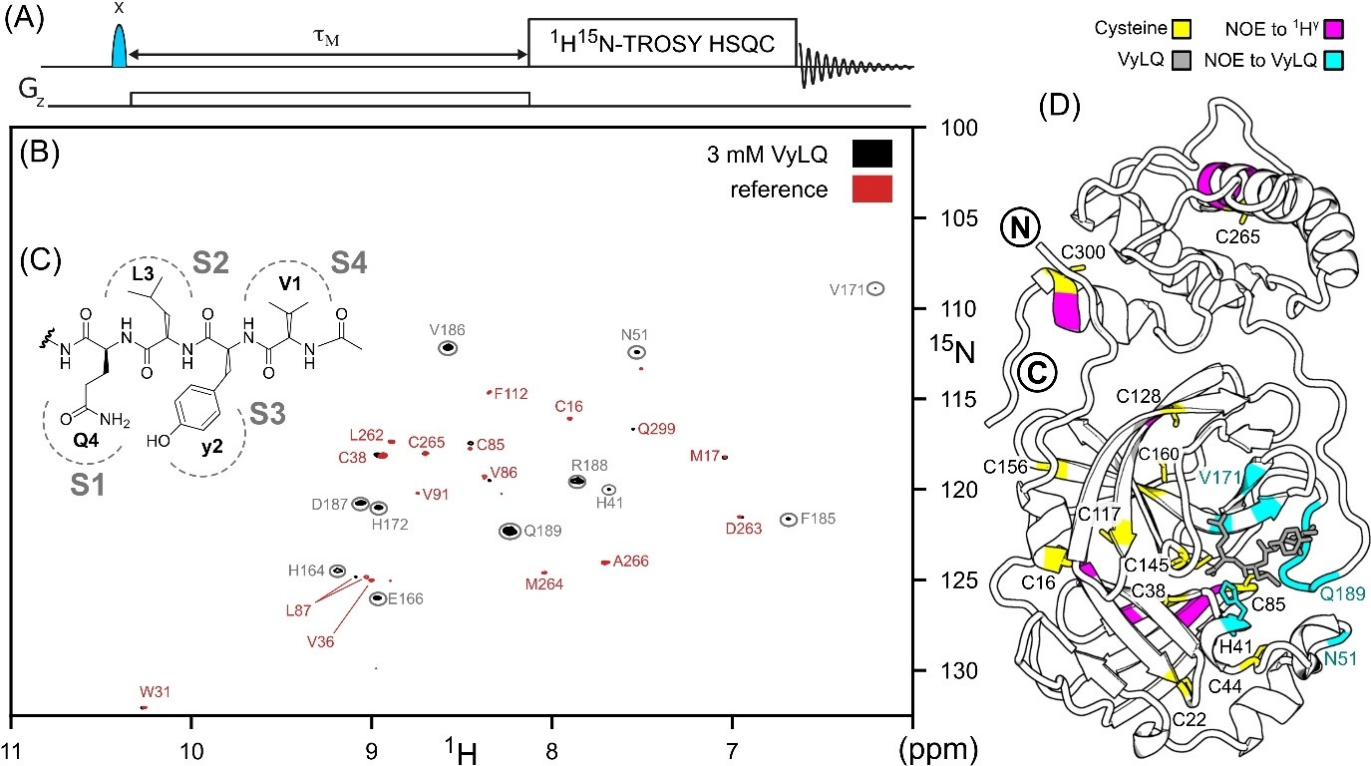
SILLY‐TROSY spectra (800 MHz, 25 °C) used to investigate the binding of VyLQ to M^pro^_C145A_ in 10 mM NaPi pH 7, 0.5 mM TCEP, 3 % D_2_O. (A) Schematic view of the SILLY element preceding a standard TROSY‐HSQC pulse scheme, with the band‐selective I‐BURP2 inversion pulse^[20]^ applied either on‐ (1.4 ppm) or off‐ (100 ppm) resonance in an interleaved manner, and a weak (0.35 G/cm) z‐gradient applied during the NOE mixing period, τ_M_. Care should be taken to eliminate water perturbation by the ‘on‐resonance’ inversion pulse because even small (∼2 %) perturbations of the water signal will lead to bleed‐through artifacts in the final spectrum and complicate analysis. The difference of spectra collected with the SILLY‐element applied off/on resonance yields the final SILLY‐TROSY spectrum. (B) Superimposed 800‐MHz SILLY‐TROSY spectra recorded for 0.3 mM (reprotonated) ^2^H^15^N‐M^pro^_C145A_ in either the absence (red) or presence (black) of 3 mM VyLQ, revealing M^pro^ backbone amides that have NOEs (τ_M_=0.5 s) to ^1^H^γ^ thiol (red labels) or aliphatic protons of the VyLQ peptide (grey circles). The long mixing time favors detection of NOEs to excess ligands in exchange between free and bound states. Shorter mixing times favor detection of NOEs to thiol protons (Figure S12). (C) VyLQ substrate analogue, with its corresponding subsite locations. (D) Residues with intra‐ (yellow; except C22 and C145) or inter‐(magenta) residue NOEs to Cys ^1^H^γ^ groups are indicated on the top ranked AF−M model of the M^pro^_C145A_:VyLQ complex (Figure S15), with both the H41/C145 catalytic dyad and the substrate peptide shown as sticks, and the N‐ and C‐termini of M^pro^ indicated with encircled N and C characters, respectively. The tetrapeptide is presented as grey sticks, with intermolecular NOEs from VyLQ to M^pro^_C145A_ colored cyan on the AF−M model.

For dimethylsulfoxide (DMSO) and weakly binding ligands such as boceprevir^[11]^ (Figures S8 and S9),^[12]^ the site of interaction on the protein is commonly identified by observing chemical shift perturbations (CSPs) of protein resonances,^[13]^ which can be correlated with ligand observations.^[14]^ The absence of CSPs is commonly used to demonstrate the lack of interaction ‐ as applies for ivermectin, which was proposed to act as a M^pro^ inhibitor based on computational modeling^[15]^ (Figure S10). For ligands where the exchange rate between free and bound species is comparable to (or slower than) the difference in protein chemical shift between the apo and ligated states, CSPs can be difficult to ascertain, particularly for larger proteins with crowded spectra. In this case, Nuclear Overhauser effects (NOEs) between ligand and protein can be used instead. For perdeuterated proteins, the sensitivity of this NOE‐based approach can be strongly enhanced by using a band‐selective pulse to simultaneously invert all upfield resonances (+3.5 to −0.7 ppm), representing most of the ligand's aliphatic hydrogens, and monitoring NOE transfer of their magnetization to protein backbone amide protons.^[16]^ Recovery of longitudinal ^1^H magnetization in large, perdeuterated proteins such as M^pro^ is dominated by efficient ^1^H−^1^H cross relaxation with hydroxyl and amide protons that rapidly exchange with the water signal, which is kept along the z‐axis by the TROSY pulse scheme,^[9b]^ permitting short delays between scans (Table S6). The band‐selective pulse also inverts protein sulfhydryl resonances (random coil chemical shift *ca* 1.9 ppm), prior to readout by the highly sensitive 2D ^1^H^15^N‐TROSY‐HSQC pulse sequence, and therefore include NOE interactions to Cys‐^1^H^γ^ resonances.^[9b]^ We refer to this method as “Selective Inversion of thioL and Ligand for NOESY” (SILLY), and the difference of spectra collected with‐ and without‐ the band‐selective inversion pulse yields the final SILLY‐TROSY spectrum. When applying the SlLLY method to M^pro^_C145A_ in the presence of a 10‐fold excess of the substrate analogue tetrapeptide N‐acetyl‐Val‐DTyr‐Leu‐Gln (VyLQ), the SILLY‐TROSY spectrum shows many amides whose CSPs are challenging to resolve without NOE‐editing (Figure 2). Because VyLQ binds the protein with moderate affinity (K_d_ ≈80 μM; Figure S11, Table S5) and the exchange between free and bound ligand is on the intermediate NMR time scale, an excess of ligand and a relatively long mixing time of 500 ms were used to maximize transfer of ligand magnetization to the protein, analogous to transferred NOE experiments.^[17]^ In contrast, magnetization from thiol protons is not replenished by exchange with free ligand and decays rapidly with increasing mixing times due to cross relaxation. NOEs from thiol to amide protons are therefore more intense at shorter mixing times such as 70 ms, where interactions are observed for the majority of Cys residues and their immediate neighbors in M^pro^_C145A_ (Figure S12). Such signals potentially provide valuable “anchors” during the early stage of sequential assignment and validation markers at a later stage, in particular for regions where sequential connectivities are sparse. Recording of the spectrum in the absence of ligand is essential to distinguish SILLY‐TROSY resonances originating from thiol and unusually upfield shifted hydroxyl protons from ligand protons. In cases where perdeuteration is less complete than achieved in our work (*ca* 98 %), weak signals resulting from NOEs to residual aliphatic sidechains may also appear in the SILLY‐TROSY spectrum.

**Figure 3:**
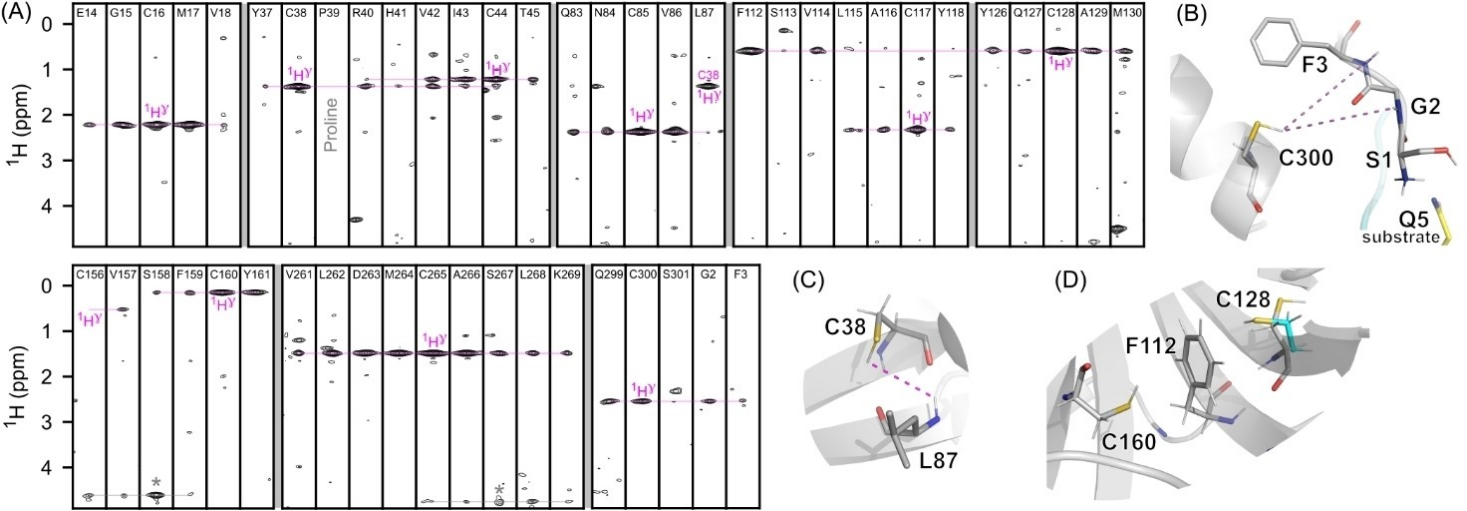
Observation of Cys−^1^H^γ^ in the M^pro^_C145A_:SAVLQSGFRK complex. (A) Narrow strips taken from the 800‐MHz ^15^N‐separated 3D NOESY‐TROSY spectrum (800 MHz, 25 °C), recorded with a 200 ms NOE mixing time, that show cross peaks from Cys−^1^H^γ^ protons (magenta annotations) to backbone ^1^H^N^ atoms of residues indicated at the top of each strip. The full set of strips is included in Figure S17. (B,C,D) Examples of observed long range interactions between thiol protons and backbone amides or aromatic groups in the AF−M model of M^pro^:SAVLQSGFRK, with long‐range NOEs between thiol‐^1^H^γ^ and amide‐^1^H^N^ atoms shown as dashed magenta lines. In (B), substrate residue Q5 (yellow carbons) occupies the S1 subsite of the companion monomer, while in (D) the alternate conformer of C128 (cyan) taken from PDB 7MNG is required to reproduce the *ca* −1.3 ppm ring current shift, rather than the AF−M model (grey).

A more detailed view of the NOE interactions with the thiol signals can be obtained from three‐dimensional (3D) ^15^N‐separated NOE spectra, recorded for an M^pro^_C145A_ sample that contained the peptide N‐acetyl‐SAVLQSGFRK (SAVLQSGFRK). This auto‐substrate peptide contains the M^pro^ N‐terminal cleavage site required to excise itself from the SARS‐CoV‐2 precursor pp1a, where S6_peptide_ corresponds to the N‐terminal residue of mature M^pro^. Affinity of M^pro^ for this peptide is high (K_d_≤4±2 μM) and NOE interactions extend from the peptide's N‐terminus to R9_peptide_ (Figure S13) validating both a PDB X‐ray structure,^[4]^ and AlphaFold‐Multimer (AF−M)^[18]^ models of this complex (Figures S14, S15). Strips taken through such spectra at the ^15^N/^1^H frequencies of selected amide groups then reveal the thiol‐^1^H^γ^ chemical shifts (Figure 3). Such resonances are valuable markers for validating resonance assignments, in particular for proteins such as M^pro^ for which good structural models are available.^[19]^ For example, C265−^1^H^γ^ shows NOEs to residues A260‐K269, spanning nearly its entire α‐helix (Figure 3A). Whereas most thiol NOEs are observed to amides proximate in sequence to each Cys, long‐range NOEs are also observed. For example, NOEs from C300‐^1^H^γ^ in the protein's C‐terminal α‐helix show proximity to N‐terminal residues G2 and F3 that are essential to M^pro^ dimerization and catalytic activity (Figure 3B). Other examples include L87, which pairs with C38 in an antiparallel β‐sheet and shows a strong NOE to the C38 thiol, and F112 which shows a strong NOE to the thiol of its β‐sheet partner C128 (Figure 3C, 3D). Many more such interactions can be seen in the full set of strips (Figures S16, S17) and are invaluable to validate the backbone resonance assignments, typically based on triple‐resonance 3D experiments such as HNCA (Figure S18).

The observation of sharp resonances for 10 of the 11 Cys sulfhydryl resonances in M^pro^_C145A_ is perhaps surprising considering that thiol protons exchange with solvent at rates that are catalyzed by both OH^−^ and phosphate and the intrinsic hydrogen exchange (HX) rate under the conditions of our sample is larger than 10^4^ s^−1^.^[21]^ Many of the thiols are buried in the protein interior and appear engaged in structure‐stabilizing hydrogen bonds. With an S−H bond length of 1.34 Å and an C^β^−S^γ^−H^γ^ bond angle of ∼95°–100°,^[22]^ thiols can act as donor for one H‐bond and acceptor for two. The sulfhydryl can also engage in interactions between an aromatic π donor‐orbital and the S−H σ* acceptor‐orbital,^[22]^ as appears to apply for C128‐H^γ^ interacting with F112‐C^ζ^ (Figure 3D). Because the proton in such interactions is situated above the ring carbon, the ring current contribution to its chemical shift (*ca* −1.3 ppm) is smaller than if positioned directly above the center of the aromatic ring, and very sensitive to its precise location. For several of the other M^pro^ thiols, the pattern of thiol‐stabilizing interactions is less clear cut. For example, with a *gauche* ^+^ χ_1_ rotamer, the C160 thiol appears to donate a third H‐bond to G149‐O, which already accepts two backbone H‐bonds from the C160 and Y161 amide groups. However, a strong NOE interaction from C160‐H^γ^ to Y161‐H^N^ in the 70‐ms mixing time SILLY‐TROSY spectrum (Figure S12) is consistent with this geometry.

The observation of strong cross peaks to thiol resonances in the 3D NOESY spectrum (Figure 3A, S17), which employed a 200 ms NOE mixing time, indicates that solvent exchange of these labile protons cannot be much faster than 5 s^−1^. The only thiol resonance missing from the 3D spectrum (Figure S17) is that of partially solvent‐exposed C22, whose intensity may also be adversely impacted by its *trans* χ_1_ rotamer, resulting in a larger intraresidue H^N^‐H^γ^ distance; rapid exchange with solvent of G23‐H^N^, which is close to C22‐S^γ^ in the X‐ray structure,^[19]^ prevents observation of this NOE. Other surface‐exposed sulfhydryls include C85, C156, and C160 which show strong NOE interactions to one or more backbone amides, indicating that intramolecular H‐bonds strongly protect these thiol protons from exchange with solvent.

The H^γ^ chemical shifts, seen in the 3D NOESY spectrum, allow multiple amides to be linked to individual thiol resonances, providing information not available from the SILLY‐TROSY 2D spectrum. However, the sensitivity of the 2D spectrum is considerably higher, as reflected in a more than 20‐fold shorter measurement time.

## Conclusion

The importance of the local structure around Cys thiol groups, which due to their reactive chemistry can play important functional roles, is well recognized.^[23]^ The positions of hydrogen atoms are typically inaccessible in X‐ray diffraction data and instead their presence is commonly modeled based on nearby H‐bond acceptor atoms, which can often be ambiguous. The clear appearance of sulfhydryl signals in NOE spectra of perdeuterated proteins provides a straightforward avenue for studying these reactive groups in more detail, while simultaneously aiding protein resonance assignment. For M^pro^, many of the thiols are close to aromatic moieties, leading to upfield ring current shifts (e. g. C128‐F112; C160‐F150; C265‐F230) or, when located near the plane of such rings, to decreased chemical shielding (C85‐F181; C300‐F3), so that chemical shifts provide another sensitive tool for probing local structure, in addition to isotope effects.^[23]^ The nearly complete backbone assignment of M^pro^ together with the introduction of two active site binding peptides with affinities of *ca* 4 and 80 μM can facilitate competitive binding NMR assays in further drug development.

## Experimental Section

The materials and methods used in our study can be found in the associated Supporting Information.

## Conflict of interest

The authors declare no conflict of interest.

1

## Supporting information

As a service to our authors and readers, this journal provides supporting information supplied by the authors. Such materials are peer reviewed and may be re‐organized for online delivery, but are not copy‐edited or typeset. Technical support issues arising from supporting information (other than missing files) should be addressed to the authors.

Supporting Information

## Data Availability

The backbone chemical shifts for M^pro^_C145A_ (BMRB: **51455**) and for M^pro^_C145A_:SAVLQSGFRK (BMRB: **51456**) complexes have been deposited in the BioMagResBank (http://www.bmrb.wisc.edu/), and additional assignments are presented in the Supporting Information.

## References

[1] S. Günther , et al., Science 2021, 372, 642–646. 33811162 10.1126/science.abf7945 PMC8224385

[2a] H. Yang , M. Yang , Y. Ding , Y. Liu , Z. Lou , Z. Zhou , L. Sun , L. Mo , S. Ye , H. Pang , G. F. Gao , K. Anand , M. Bartlam , R. Hilgenfeld , Z. Rao , Proc. Natl. Acad. Sci. USA 2003, 100, 13190–13195; 14585926 10.1073/pnas.1835675100 PMC263746

[2b] C.-Y. Chou , H.-C. Chang , W.-C. Hsu , T.-Z. Lin , C.-H. Lin , G.-G. Chang , Biochemistry 2004, 43, 14958–14970. 15554703 10.1021/bi0490237

[3a] J. Shi , J. Sivaraman , J. Song , J. Virol. 2008, 82, 4620–4629; 18305031 10.1128/JVI.02680-07 PMC2293028

[3b] T. Hu , Y. Zhang , L. Li , K. Wang , S. Chen , J. Chen , J. Ding , H. Jiang , X. Shen , Virology 2009, 388, 324–334. 19409595 10.1016/j.virol.2009.03.034 PMC7103376

[4] S. Chen , F. Jonas , C. Shen , R. Hilgenfeld , Protein Cell 2010, 1, 59–74. 21203998 10.1007/s13238-010-0011-4 PMC4875104

[5a] K. Anand , J. Ziebuhr , P. Wadhwani , J. R. Mesters , R. Hilgenfeld , Science 2003, 300, 1763–1767; 12746549 10.1126/science.1085658

[5b] T. Muramatsu , C. Takemoto , Y. T. Kim , H. Wang , W. Nishii , T. Terada , M. Shirouzu , S. Yokoyama , Proc. Natl. Acad. Sci. USA 2016, 113, 12997–13002. 27799534 10.1073/pnas.1601327113 PMC5135343

[6] X. Deng , S. E. StJohn , H. L. Osswald , A. O′Brien , B. S. Banach , K. Sleeman , A. K. Ghosh , A. D. Mesecar , S. C. Baker , J. Virol. 2014, 88, 11886–11898. 25100843 10.1128/JVI.01528-14 PMC4178758

[7] Z. Jin , X. Du , Y. Xu , Y. Deng , M. Liu , Y. Zhao , B. Zhang , X. Li , L. Zhang , C. Peng , Y. Duan , J. Yu , L. Wang , K. Yang , F. Liu , R. Jiang , X. Yang , T. You , X. Liu , X. Yang , F. Bai , H. Liu , X. Liu , L. W. Guddat , W. Xu , G. Xiao , C. Qin , Z. Shi , H. Jiang , Z. Rao , H. Yang , Nature 2020, 582, 289–293. 32272481 10.1038/s41586-020-2223-y

[8a] F. X. Cantrelle , E. Boll , L. Brier , D. Moschidi , S. Belouzard , V. Landry , F. Leroux , F. Dewitte , I. Landrieu , J. Dubuisson , B. Deprez , J. Charton , X. Hanoulle , Angew. Chem. Int. Ed. 2021, 60, 25428–25435; 10.1002/anie.202109965 PMC8653025 34570415

[8b] A. J. Robertson , J. M. Courtney , Y. Shen , J. Ying , A. Bax , J. Am. Chem. Soc. 2021, 143, 19306–19310. 34757725 10.1021/jacs.1c10588 PMC8592127

[9a] K. Pervushin , R. Riek , G. Wider , K. Wüthrich , Proc. Natl. Acad. Sci. USA 1997, 94, 12366–12371; 9356455 10.1073/pnas.94.23.12366 PMC24947

[9b] K. V. Pervushin , G. Wider , K. Wuthrich , J. Biomol. NMR 1998, 12, 345–348. 21136330 10.1023/A:1008268930690

[10] E. Schmidt , P. Guntert , J. Am. Chem. Soc. 2012, 134, 12817–12829. 22794163 10.1021/ja305091n

[11] L. Fu , F. Ye , Y. Feng , F. Yu , Q. Wang , Y. Wu , C. Zhao , H. Sun , B. Huang , P. Niu , H. Song , Y. Shi , X. Li , W. Tan , J. Qi , G. F. Gao , Nat. Commun. 2020, 11, 4417. 32887884 10.1038/s41467-020-18233-x PMC7474075

[12] S. Keiffer , M. G. Carneiro , J. Hollander , M. Kobayashi , D. Pogoryelev , E. Ab , S. Theisgen , G. Muller , G. Siegal , J. Biomol. NMR 2020, 74, 521–529. 32901320 10.1007/s10858-020-00343-9 PMC7683447

[13] M. V. Schoenle , Y. Li , M. Yuan , M. W. Clarkson , I. A. Wilson , W. Peti , R. Page , J. Am. Chem. Soc. 2021, 143, 7930–7934. 34018723 10.1021/jacs.1c03945 PMC12624566

[14] S. Sreeramulu , C. Richter , H. Berg , M. A. Wirtz Martin , B. Ceylan , T. Matzel , J. Adam , N. Altincekic , K. Azzaoui , J. K. Bains , M. J. J. Blommers , J. Ferner , B. Furtig , M. Gobel , J. T. Grun , M. Hengesbach , K. F. Hohmann , D. Hymon , B. Knezic , J. N. Martins , K. R. Mertinkus , A. Niesteruk , S. A. Peter , D. J. Pyper , N. S. Qureshi , U. Scheffer , A. Schlundt , R. Schnieders , E. Stirnal , A. Sudakov , A. Troster , J. Vogele , A. Wacker , J. E. Weigand , J. Wirmer-Bartoschek , J. Wohnert , H. Schwalbe , Angew. Chem. Int. Ed. 2021, 60, 19191–19200. 10.1002/anie.202103693 PMC8426693 34161644

[15] A. F. Eweas , A. A. Alhossary , A. S. Abdel-Moneim , Front. Microbiol. 2020, 11, 592908. 33746908 10.3389/fmicb.2020.592908 PMC7976659

[16] K. Weinhäupl , C. Lindau , A. Hessel , Y. Wang , C. Schütze , T. Jores , L. Melchionda , B. Schönfisch , H. Kalbacher , B. Bersch , D. Rapaport , M. Brennich , K. Lindorff-Larsen , N. Wiedemann , P. Schanda , Cell 2018, 175, 1365–1379.e1325. 30445040 10.1016/j.cell.2018.10.039 PMC6242696

[17] J. Anglister , G. Srivastava , F. Naider , Prog. Nucl. Magn. Reson. Spectrosc. 2016, 97, 40–56. 27888839 10.1016/j.pnmrs.2016.08.002

[18] R. Evans , M. O'Neill , A. Pritzel , N. Antropova , A. Senior , T. Green , A. Žídek , R. Bates , S. Blackwell , J. Yim , O. Ronneberger , S. Bodenstein , M. Zielinski , A. Bridgland , A. Potapenko , A. Cowie , K. Tunyasuvunakool , R. Jain , E. Clancy , P. Kohli , J. Jumper , D. Hassabis , bioRxiv 2021, 10.1101/2021.10.04.463034.

[19] A. Douangamath , D. Fearon , P. Gehrtz , T. Krojer , P. Lukacik , C. D. Owen , E. Resnick , C. Strain-Damerell , A. Aimon , P. Abranyi-Balogh , J. Brandao-Neto , A. Carbery , G. Davison , A. Dias , T. D. Downes , L. Dunnett , M. Fairhead , J. D. Firth , S. P. Jones , A. Keeley , G. M. Keseru , H. F. Klein , M. P. Martin , M. E. M. Noble , P. O′Brien , A. Powell , R. N. Reddi , R. Skyner , M. Snee , M. J. Waring , C. Wild , N. London , F. von Delft , M. A. Walsh , Nat. Commun. 2020, 11, 5047. 33028810 10.1038/s41467-020-18709-w PMC7542442

[20] H. Geen , R. Freeman , J. Magn. Reson. 1991, 93, 93–141.

[21] J. Chen , N. N. Yadav , T. Stait-Gardner , A. Gupta , W. S. Price , G. Zheng , NMR Biomed. 2020, 33, e4188. 31793114 10.1002/nbm.4188

[22] C. R. Forbes , S. K. Sinha , H. K. Ganguly , S. Bai , G. P. A. Yap , S. Patel , N. J. Zondlo , J. Am. Chem. Soc. 2017, 139, 1842–1855. 28080040 10.1021/jacs.6b08415 PMC5890429

[23] M. Takeda , J. Jee , T. Terauchi , M. Kainosho , J. Am. Chem. Soc. 2010, 132, 6254–6260. 20384326 10.1021/ja101205j

