# Supplementary material for: NMR Observation of Sulfhydryl Signals in SARS‐CoV‐2 Main Protease Aids Structural Studies
Source: Chembiochem. 2022 Sep 7:e202200471. Online ahead of print. doi: 10.1002/cbic.202200471 (PMC9537880; doi:10.1002/cbic.202200471)
Supplement: Supplementary file 1 — Supporting Information [file CBIC-9999-0-s001.pdf]

ChemBioChem

Supporting Information

NMR Observation of Sulfhydryl Signals in SARS-CoV-2 Main Protease Aids Structural Studies

Angus J. Robertson, Jinfa Ying, and Ad Bax*

Table of Contents

Table of Contents 2
Experimental Procedures 3
Figure S1 .... Error! Bookmark not defined.
Figure S2 9
Figure S3 10
Figure S4 .... Error! Bookmark not defined.
Figure S5 .... Error! Bookmark not defined.
Figure S6 .... Error! Bookmark not defined.
Figure S7 .... Error! Bookmark not defined.
Figure S8 15
Figure S9 .... Error! Bookmark not defined.
Figure S10 .... Error! Bookmark not defined.
Figure S11 .... Error! Bookmark not defined.
Figure S12 .... Error! Bookmark not defined.
Figure S13 .... Error! Bookmark not defined.
Figure S14 .... Error! Bookmark not defined.
Figure S15 22
Figure S16 .... Error! Bookmark not defined.
Figure S17 .... Error! Bookmark not defined.
Figure S18 .... Error! Bookmark not defined.
Table S1 .... Error! Bookmark not defined.
Table S2 .... Error! Bookmark not defined.
Table S3 .... Error! Bookmark not defined.
Table S4 .... Error! Bookmark not defined.
Table S5 .... Error! Bookmark not defined.
Table S6 .... Error! Bookmark not defined.
Author Contributions 52
References 52

Experimental Procedures

Expression construct

The gene encoding an M^{pro}-fusion protein (GenScript, USA) and was cloned into a Pet24a+ plasmid between BamH1 and Xho1 restriction sites. The fusion protein encoded for 6His tag – GB1 – SG rich linker – TEV cleavage site – M^{pro}. Initial attempts to express wild-type M^{pro} at 37 °C, 30 °C, and 25 °C were unsuccessful, with only the N-terminal GB1 fragment present at the point of cell lysis (seemingly cleaved at the TEV recognition site). The C145A variant of M^{pro} (M^{pro}_{C145A}) was generated using NEB base changer kit with GAACGGTTCTGCTGGTTCCGTCG, and AAGAACGAGCCTTTGATC primers (T_m (T_{anneal}); 57(58)°C, 59(58)°C, respectively) ordered from IDTDNA to introduce the mutation. Successful mutagenesis was confirmed by DNA sequencing (Quintra Bio) and the observation of protein expression. This plasmid was used for initial expressions of M^{pro}_{C145A} without reprotonation of buried amides, a second plasmid was used for subsequent recombinant protein expression, where GenScript (HK) had performed the same site-directed mutagenesis. An overview of the insertion fragment is presented below.

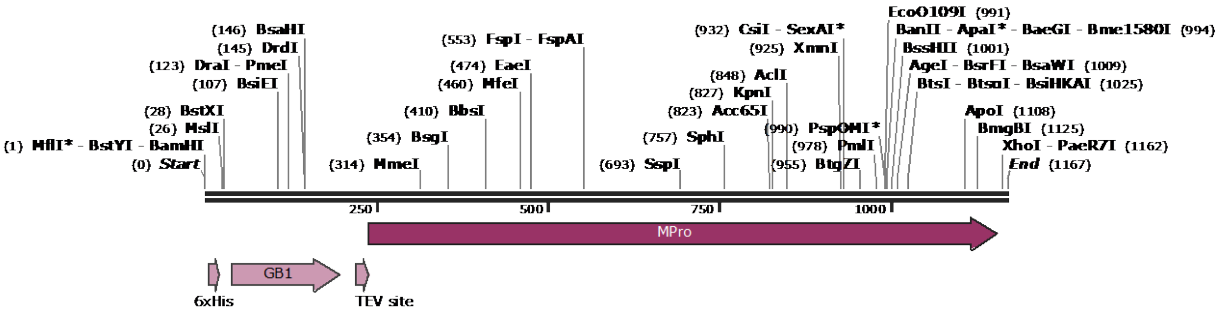

A more detailed sequence map of the N-terminal leader sequence is presented overleaf (generated using SnapGene).

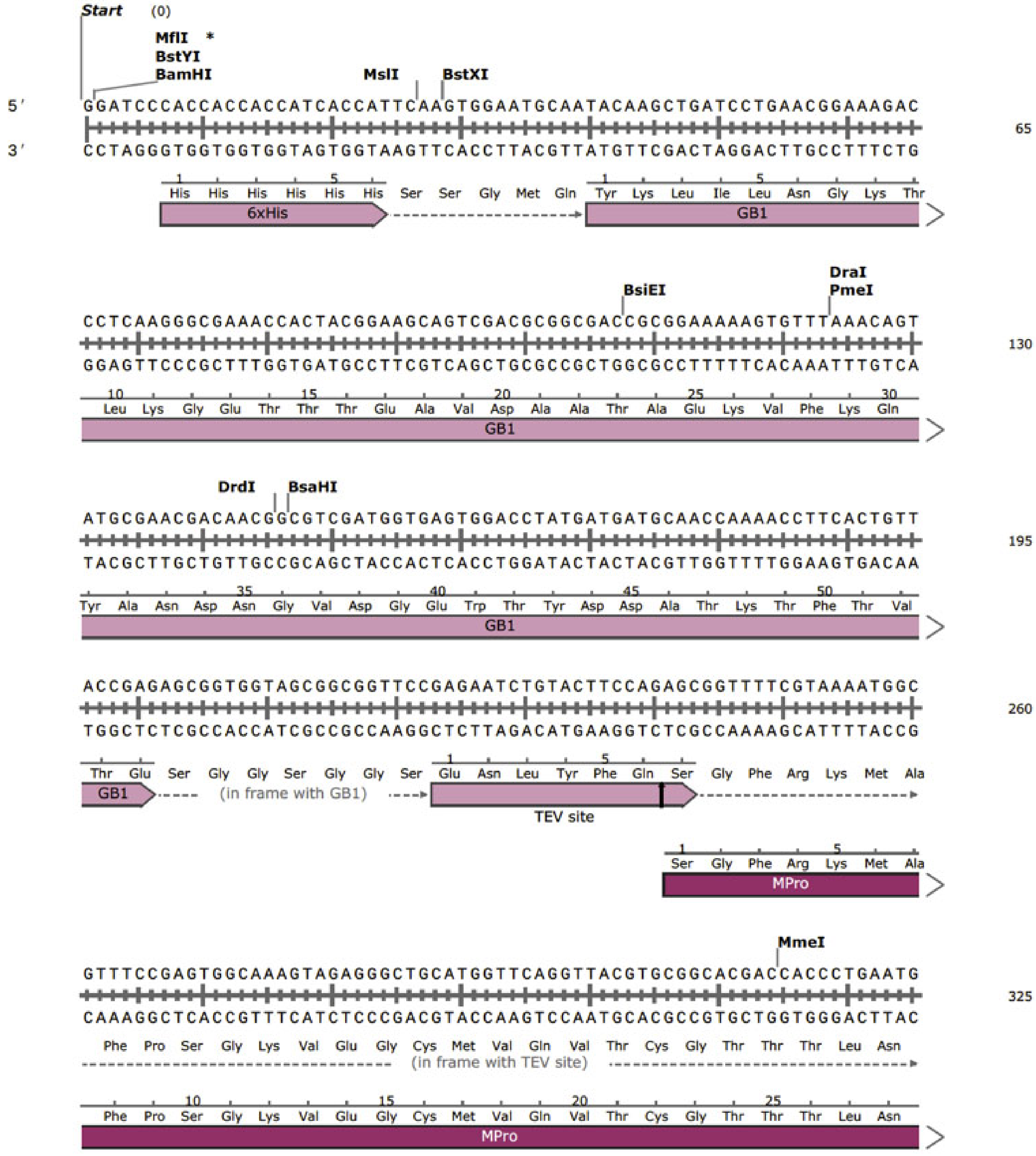
Start (0)
MflI *
BstYI
BamHI
MslI
BstXI
5′
GGATCCCACCACCACCACCATCACCATTCAAGTGGAATGCAATACAAGCTGATCCTGAACGGAAAGAC
3′
CCTAGGGTGGTGGTGGTAGTGGTAAGTTCACCTTACGTTATGTTCGACTAGGACTTGCCTTTCTG
65
His His His His His His Ser Ser Gly Met Gln Tyr Lys Leu Ile Leu Asn Gly Lys Thr
6xHis
GB1
BsiEI
DraI
PmeI
CCTCAAGGGCGAAACCACTACGGAAGCAGTCGACGCGGCGACCGCGGAAAAAGTGTTTAAACAGT
GGAGTTCCCGCTTTGGTGATGCCTTCGTCAGCTGCGCCGCTGGCGCCTTTTTCACAAATTTGTCA
130
Leu Lys Gly Glu Thr Thr Thr Glu Ala Val Asp Ala Ala Thr Ala Glu Lys Val Phe Lys Gln
GB1
DrdI
BsaHI
ATGCGAACGACAACGGCGTCGATGGTGAGTGGACCTATGATGATGCAACCAAAACCTTCACTGTT
TACGCTTGCTGTTGCCGCAGCTACCACTCACCTGGATACTACTACGTTGGTTTTGGAAGTGACAA
195
Tyr Ala Asn Asp Asn Gly Val Asp Gly Glu Trp Thr Tyr Asp Asp Ala Thr Lys Thr Phe Thr Val
GB1
ACCGAGAGCGGTGGTAGCGGCGGTTCCGAGAATCTGTACTTCCAGAGCGGTTTTCGTAAAATGGC
TGGCTCTCGCCACCATCGCCGCCAAGGCTCTTAGACATGAAGGTCTCGCCAAAAGCATTTTACCG
260
Thr Glu Ser Gly Gly Ser Gly Gly Ser Glu Asn Leu Tyr Phe Gln Ser Gly Phe Arg Lys Met Ala
GB1
(in frame with GB1)
TEV site
Ser Gly Phe Arg Lys Met Ala
MPro
MmeI
GTTTCCGAGTGGCAAAGTAGAGGGCTGCATGGTTCAGGTTACGTGCGGCACGACCACCCTGAATG
CAAAGGCTCACCGTTTCATCTCCCGACGTACCAAGTCCAATGCACGCCGTGCTGGTGGGACTTAC
325
Phe Pro Ser Gly Lys Val Glu Gly Cys Met Val Gln Val Thr Cys Gly Thr Thr Thr Leu Asn
(in frame with TEV site)
Phe Pro Ser Gly Lys Val Glu Gly Cys Met Val Gln Val Thr Cys Gly Thr Thr Thr Leu Asn
MPro

Sample preparation

Plasmid containing M^{pro} was transformed into BL21(DE3) (Agilent) using a standard transformation protocol (Agilent) and a single colony was selected from LB (50 ug/mL Kanamycin) plates and used for subsequent culture. $^{2}H^{13}C^{15}N$ labelled M^{pro}_{C145A} was grown in the following M9 medium, pH adjusted to 7.4*: Na_2HPO_4 (6 g/L), KH_2PO_4 (3g/L), NaCl (0.5 g/L), trace elements[1] (0.6 mL /L), $MgSO_4$ (1 mM), $CaCl_2$ (0.1 mM), $^{15}NH_4Cl$ (1 g/L), $^{2}H^{13}C$ glucose (2g/L), Isogro (10%, 1mL/L) Kanamycin Sulfate (50 ug/L). Cells were acclimatized to deuterated media through a series of small scale starter cultures (0% D_2O, 90% D_2O and 100% D_2O) as outlined previously,[1-2] with a full scale culture (100% D_2O) of 500 mL in 2L UltraYield flasks (Thomas Scientific) shaken at 37 °C and 220 rpm until OD_{600} = 0.8, at which point the cells were induced with isopropyl β-d-1-thiogalactopyranoside (IPTG) to a final concentration of 1 mM. Cells were incubated either for a further 4h at 37 °C, or overnight at 25 °C and were harvested by centrifugation at 4,500 rpm for 30 min and frozen at -80 °C overnight. The two incubation protocols resulted in similar yields.

Cell pellets were resuspended in 25 mL of **Buffer A** (20 mM Tris pH 7.5, 150 mM NaCl, 20 mM Imidazole, 0.5 mM TCEP) supplemented with one tablet of cOmplete protease inhibitor cocktail (Roche). The cell suspension was lysed on ice by sonication (12 cycles of 30s on, 30s off) and the insoluble fraction was removed by centrifugation at 19,000 rpm for 1 hour at 4 °C (Beckman Coulter Avanti centrifuge JA-25.50). The soluble fraction was loaded (2 mL/min) onto two concatenated 5 ml HisTrap HP columns (GE Healthcare) pre-equilibrated in Buffer A using a 50 mL SuperLoop (GE Healthcare). After loading, the column was washed with 60 mL Buffer A; then the protein was eluted using a step gradient to **Buffer B** (20 mM Tris pH 7.5, 150 mM NaCl, 300 mM Imidazole, 0.5 mM TCEP). The peak elutes across 3 x 6 mL fractions which were then pooled and diluted to 3 mg/mL protein (using A280 = 1.2×10^3 for 1 mg/mL).

To this solution, pre-dissolved 1M dithiothreitol (DTT) was added to a final concentration of 20 mM, with gentle swirling to mix the two solutions. Next, degassed **Buffer C** (50 mM Tris pH 8, 1.5 M Guanidine HCl, 10% glycerol) was added in a 6:4 ratio, and incubated for 4-48h at room temperature without shaking in a 100 mL glass Duran sealed under N_2 (g) (a method adapted from the approach used for PETNR [3]). Extensive reprotonation was observed after 4 hours, with additional reprotonation observed for a subset of resonances after extending the incubation period to 48 hours. However, numerous buried residues in required an additional procedure to achieve full back exchange (see below). The reaction mix, typically *ca.* 60 mL was added to a 70 mL Slide-A-Lyzer dialysis cassette (10 kDa cutoff; Thermo Scientific), followed by dialysis against 4L (N_2(g) degassed) **Buffer D** (PBS supplemented with 0.5 mM TCEP) for 2h at room temperature to remove the majority of the GuHCl. Subsequently, 0.5 mg of TEV protease and 10 mM DTT was added to the dialysis cassette and the reaction mixture was dialyzed against a fresh 4L of N_2 degassed Buffer D overnight at 4 °C (TEV expressed in-house).

The reaction mixture was then loaded back onto the cleaned (Buffer B; supplemented with 8M urea) HisTrap column pre-equilibrated in Buffer A. The flow through was collected and concentrated to *ca.* 10 mL using a 10 kDa MWCO Amicon centrifugal concentrator, before being loaded onto a Hiload 26/60 Superdex G75 size-exclusion column previously washed with 1 column volume of 1 M NaOH and equilibrated in **Buffer E** (10 mM NaPi pH 7.0, 0.5 mM TCEP). Fractions containing M^{pro}_{C145A} were checked for purity using SDS-PAGE and pooled together, before being concentrated to ~ 1 mM by Amicon centrifugal concentrator (10 kDa MWCO) and flash frozen in liquid nitrogen for storage at − 80 °C. Protein concentrations were estimated by absorbance at 280 nm (ε_{280} = 32890 M^{-1} cm^{-1}) which assumed all cysteines were reduced.

All chemical reagents were of analytical grade and were purchased from Sigma-Aldrich (USA), CortecNet (USA), or Fischer.

Full back exchange of amide protons

Despite the presence of roughly the expected number of backbone amide peaks in the TROSY-HSQC spectrum, initial assignments revealed that *ca.* 40 residues were missing in buried β-sheet regions, as well as in the C-terminal α-helical domain, whereas residues in the linker between domains II and III showed duplicate resonances. This indication of incomplete back exchange was later confirmed when an improved back-exchange protocol was developed for this protein. This method was adapted from the approach taken for another slowly back-exchanging globular protein, PETNR[3], with minor modifications to adapt it for M^{pro}. Initial work characterizing the 2002 variant of M^{pro} [4] indicated that the 0.75M GuHCl would be sufficient to substantially unfold the catalytic N-terminal domain of the monomer, but would be insufficient to unfold the helical C-terminal domain (in our hands 0.9 – 1.1 M was the highest GuHCl that could be used, but at the top of this range, a substantial increase of protein precipitation was observed). Using these observations, and the presence of the solubility-tag / IMAC-tag at the N-terminus, an unfolding – refolding step was introduced into the middle of the purification protocol. It should be noted that, unfolding of final purified protein product was observed to be effectively irreversible under standard conditions and temperatures, as were unfolding attempts using > 1.1M GuHCl and pH values over 8, as DTT and TCEP no-longer protect the 11 remaining Cys residues. Moreover, attempts to refold the fusion construct on the His-trap column were unsuccessful, likely limited by the permissible concentration of reducing agent and high effective protein concentration on the column.

The optimized protocol involved a step-elution from the IMAC column to retain a relatively high concentration (frequent elution at *ca.* 5 mg/mL) to avoid the need for a concentration step. A similar unfolding-refolding protocol was employed as for PETNR [3], namely, the protein was diluted to 2 mg/mL at pH 8 and slowly added 1:1 to an equal volume of buffered 2.0 M GuHCl - to a total volume of ca. 70 mL. This mixture (both initial buffers containing 10-20 mM DTT) was incubated for 2d at room temperature in a glass Duran. Rather than snap-refolding the back-exchanged protein by dilution (as for PETNR[3]; necessitating extensive concentration), we found that dialysis at room temperature for 2h in a 70mL dialysis cassette resulted in minimal protein precipitation. At this point, TEV protease was added to this dialysis cassette for the proteolytic cleavage of the N-terminal solubility/affinity tag overnight at 4 ºC (see methods

for full details). Retention of the N-terminal solubility tag (unaffected by the increase in GuHCl concentration) for the unfolding step was important, preventing the partially unfolded peptide population from substantive aggregation (<1% observed). Following the cleavage reaction, the reaction mixture was passed back down an IMAC column which removed the TEV and (small population of) uncleaved M^{pro}_{C145A} fusion construct. The following concentration and gel filtration / buffer exchange steps are common practice (see methods). Nearly complete back exchange of backbone amides was indicated by the presence of many new peaks in the TROSY NMR spectrum (Figure S1), and was further substantiated on collection of triple resonance backbone assignment spectra and NOE spectra. Several very weak, additional peaks observed in the reprotonated spectrum (Figure S1) correspond to minor species that may result from the relatively harsh conditions during the reprotonation protocol. Their origin has not been further investigated. In both the main text and the Supporting Information, distinction is made between samples that either were (reprotonated) or were not (not-reprotonated) exposed to this partial refolding step, with samples being reprotonated unless otherwise stated. Because the reprotonation protocol was only developed towards the end of our study of M^{pro}, spectra obtained during the early phase of our study were recorded on non-reprotonated protein. The absence of chemical shift perturbation between spectra of reprotonated and non-reprotonated samples indicates that the reprotonation protocol does not impact the structure or properties of the protein.

NMR experiments

Backbone assignment spectra were acquired on 1.1 mM ^{2}H,^{15}N,^{13}C-labelled M^{pro}_{C145A} samples in **NMR buffer** (10 mM $NaPO_4$, 0.5 mM TCEP buffer (pH 7.0) supplemented with $^{2}H_2O$ (3% v/v) and 0.3 mM sodium 3-(trimethylsilyl)propane-1-sulfonate (DSS)), but sample conditions are often repeated in figure captions for convenience. All assignment experiments were recorded at 298K unless otherwise stated. TROSY versions of the standard HNCO, HN(CA)CO, HNCA, and HNCB experiments [5] were recorded on a 700 MHz Bruker Avance III spectrometer equipped with a 5-mm TCI probe containing triple-axis gradients and running TopSpin software version 3.2. Additionally, ^{1}H NOESY-TROSY experiments were acquired on a 900 MHz (800 MHz) Bruker spectrometer with a Neo (Avance III) console, fitted with a 5-mm TCI probe equipped with single-axis (triple-axis) gradients and running TopSpin software version 4.1 (3.1).

Backbone $^{1}H^{N}$, ^{15}N, $^{13}C'$, $^{13}C^{\alpha}$, and $^{13}C^{\beta}$ chemical shifts were assigned for substrate-free M^{pro}_{C145A} using the standard triple resonance methodology (Gardner and Kay 1998). Spectra were processed in NMRPipe,[6] peak picking was performed in SPARKY [7] and POKY.[8] Frequency matching of the backbone assignments was achieved in an iterative manner, using FLYA [9] and manual checking in both Sparky and NMRView. SPARTA+ [10] was used to generate chemical shift predictions from PDB entry 6Y84 which were used by FLYA to guide the assignment strategy. SPARTA+ predicted chemical shifts were also used to cross-check the final assignments, along with comparison to the equivalent residues in the previously deposited BMRB entries (BMRB entries **17251**[11] and **17911**).

The backbone $^{1}H_N$, ^{15}N, $^{13}C'$, $^{13}C^{\alpha}$, $^{13}C^{\beta}$ chemical shifts have been deposited in the BioMagResBank (http://www.bmrb.wisc.edu/) for M^{pro}_{C145A} (BMRB: **51455**) and for M^{pro}_{C145A}:SAVLQSGFRK (BMRB: **51456**) complexes. Excluding the 13 proline residues and the N-terminal serine residue, 284 out of a possible 292 residues were assigned in the $^{1}H^{15}N$ TROSY spectrum of $M^{pro}_{C145A.}$ In total, 97.6% of all backbone resonances were assigned (97.3% $^{1}H_N$, 97.3% ^{15}N, 93.5% $^{13}C'$, 98.7% $^{13}C_{\alpha}$, 86.6% $^{13}C_{\beta}$ nuclei).

Programs for visualization and analysis were written using freely available python libraries,[12] as well as NMR-specific python libraries.[13]

AlphaFold-Multimer calculations

The installation of AlphaFold-2,[14] previously used by us, [15] was modified according to the protocol outlined by Evans et al.[16] and using an available online resource: https://github.com/jcheongs/alphafold-multimer (date retrieved: 2022/06/22). The full implementation of AlphaFold-Multimer (AF-M) was used (including Amber [17] forcefield relaxation, and without sequence restriction). AF-M calculations were performed for both (monomeric; 3h) M^{pro}:SAVLQSGFRK and (dimeric; 15h) $2M^{pro}$:SAVLQSGFRK complexes, on a modest graphics card (NVIDIA RTX1080), and the predicted structures (SI Fig. S15) were close to PDB entry 2Q6G (SI Fig. S14), with non-H atom RMSD values for M^{pro} (monomer; dimer) of (0.51Å; 0.84Å), and substrate (1.7Å; 3.9Å). The same process was repeated for VyLQ substrate analog. AlphaFold reports per-residue predicted local-distance difference test (pLDDT) scores that quantify confidence in local structure prediction,[14] which correlates with solution structure accuracy.[15, 18] pLDDT scores are consistently high across the monomeric and dimeric M^{pro} models, however only the dimeric M^{pro} complex confidently predicts the N- and C- termini which occupy the inter-monomer interface. pLDDT scores for the decapeptide are high for pockets S4-S1' in the monomer, with a relatively tight clustering of binding poses across the AF-M predictions (SI Fig. S15). However, both clustering and pLDDT confidence scores deteriorate in the dimeric complex (SI Fig. S15). The final step of AF-M structure prediction is an energy minimization using Amber, which requires a fully protonated protein. Experimentally, proton positions are only accessible using exotic neutron-scattering experiments, rendering X-ray structures devoid of protons and requiring *post-hoc* software solutions for accurate proton placement (eg. reduce,[19] or DYNAMO[20]). Consequently, the comparison between AF-M and X-ray should be restricted to non-H atoms.

Backbone Assignment Strategy

Several issues emerged due to repeated peak usage by the FLYA algorithm, so the *keepassigned=true* option was used in conjunction with a *N15HSQCassigned.peaks* reference shift file in order to transition to a semi-automated assignment protocol. Two

states were observed with *ca* 9:1 intensity ratio in the TROSY-HSQC spectrum, centered around Pro184. Inspection of the $^{13}C^{\beta}$(i-1) peak of F185 revealed that the two states were due to cis-trans isomerization of P184, as evidenced by a substantial downfield $^{13}C^{\beta}$ shift which is highly diagnostic. Increased flexibility of residues in the vicinity of P184 caused many of the minor cis conformer resonances to have stronger intensities than amides in other regions of the protein that were impacted by exchange broadening.

Given the relatively large range of intensities observed for M^{pro}, this weaker *Minor* form therefore complicated the initial assignment, and precluded the effective use of many common assignment packages. *Minor* states were manually sought, identified, and excluded from the pool of spin systems, leading to a final coverage of 97.3% of all backbone resonances (97.3% $^{1}H_{N}$, 97.3% ^{15}N, 93.5% $^{13}C'$, 98.7% $^{13}C_{\alpha}$, 86.6% $^{13}C_{\beta}$ nuclei), with 20 residues displaying a minor conformer. The missing residues are largely localized to the active site.

Chemical shift perturbation

In keeping with other approaches to combine chemical shift perturbations from multiple incomplete sets of heteronuclei,[21] we used the following equation to quantify the overall chemical shift perturbation (CSP):

$$CSP = \sqrt{\frac{1}{N}\sum_{i=1}^{N}(\delta_i/\alpha_i)^2}$$

where for a given residue, N is the number of atoms available for comparison, α_i is the RMS of secondary shifts in folded proteins in the SPARTA+ database,[10] which are 1.04, 1.16, 1.14, 2.56 and 0.54 ppm for $^{13}C^{\alpha}$, $^{13}C^{\beta}$, $^{13}C'$, ^{15}N and $^{1}H^{N}$, respectively.

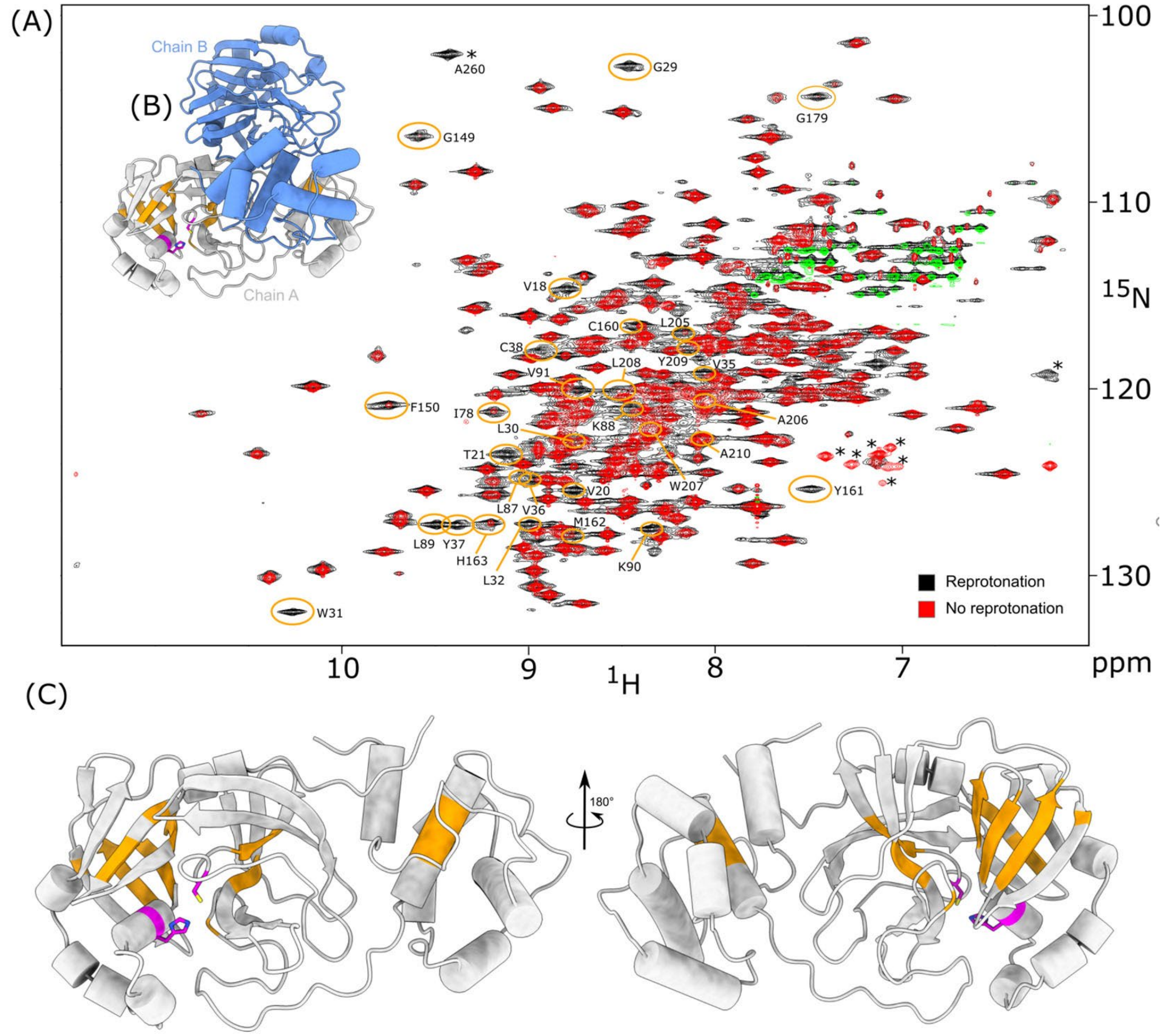

Figure S1: Location of residues that require unfolding for full reprotonation of $^{2}H^{15}N$-M^{pro}_{C145A} in 10 mM NaPi pH 7.0 buffer supplemented with 0.5 mM TCEP and 0.3 mM DSS for chemical shift referencing. (A) Overlay of fully reprotonated (black) and non-reprotonated (red) $^{1}H^{15}N$-TROSY-HSQC spectra. Asterisks denote sidechain peaks that were aliased, while the backbone amide peak of A260 is aliased from 136.2 ppm and is fully reprotonated without unfolding. For improved visualization of overlayed resonances in both the reprotonated and non-reprotonated spectra, slightly more aggressive apodization in the ^{1}H dimension for the non-reprotonated spectrum was used. Sidechain amide resonances of Gln and Asn that are incompletely suppressed by the TROSY pulse scheme are shown in green. (B) Illustration of the M^{pro} homodimer with slowly reprotonating residues (time constant > 3 months) colored orange, with separate monomer chains colored grey and blue for clarity, and the catalytic dyad of C145A and H41 colored magenta. (C) A single-chain representation of (B).

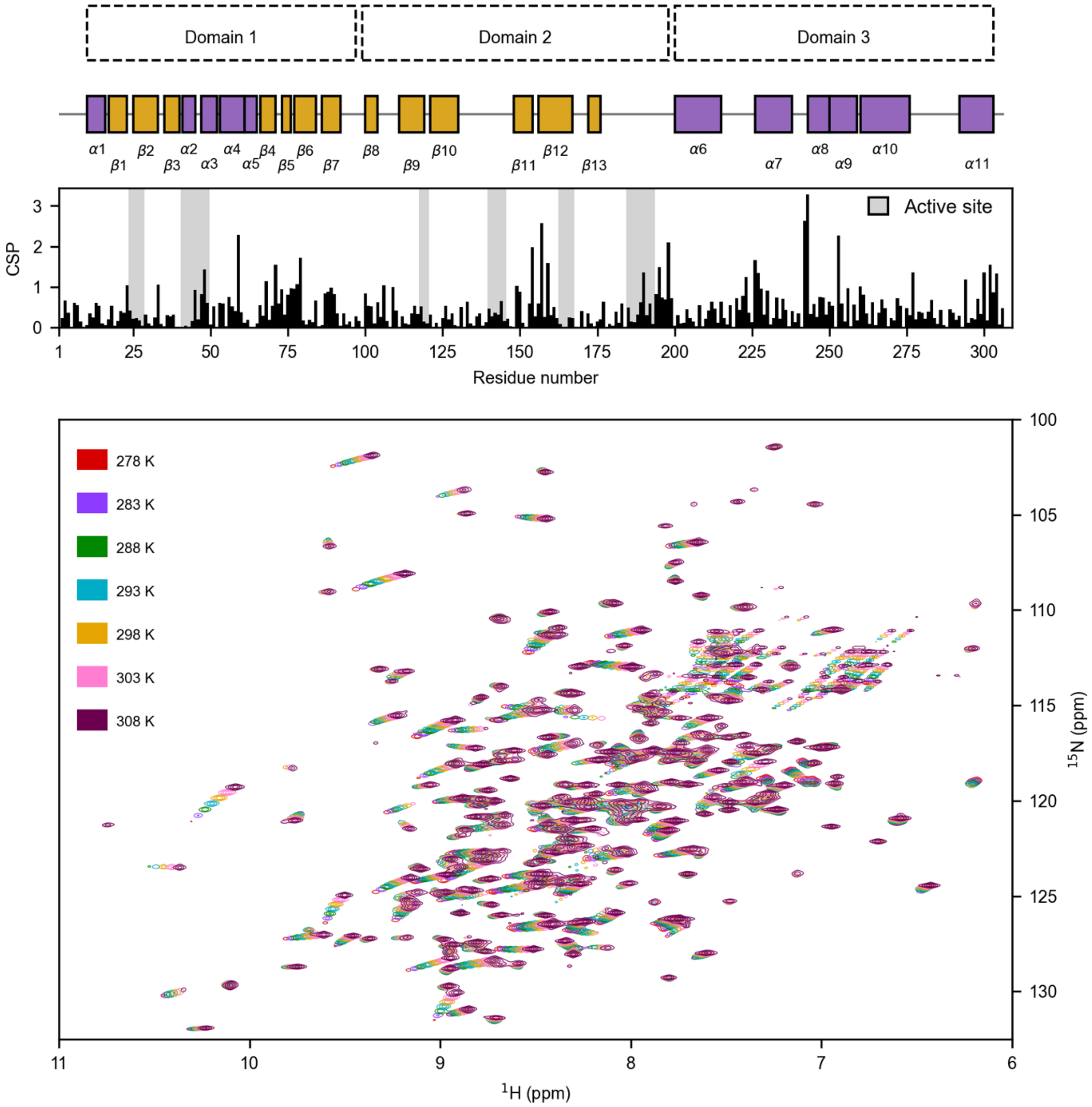

Figure S2: Temperature titration of (reprotonated) $^{2}H^{15}N^{13}C$-M^{pro}_{C145A} in 10 mM NaPi pH 7.0, 0.5 mM TCEP, 0.3 mM DSS, 3% D_2O at 900 MHz (bottom). The chemical shift perturbation (CSP (ppm)) bar plot (top) compares 278K and 308K titration points, with secondary structure elements extracted from PDB entry 5R8T, and the active site region indicated in cyan.

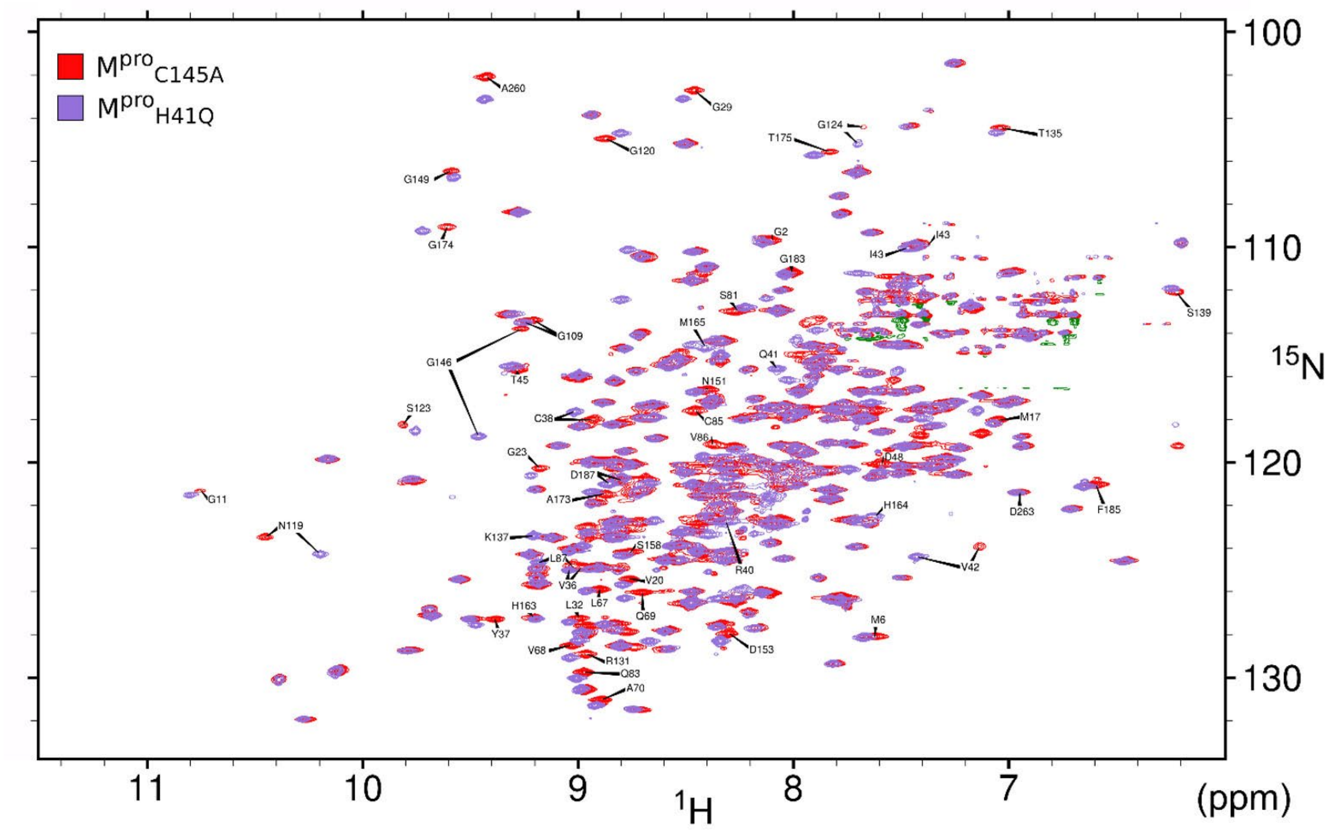

Figure S3: Overlay of reprotonated $^2H^{15}N$-labelled M^{pro}_{C145A} (red) and M^{pro}_{H41Q} (violet) with annotation of residues that either display moderate to large chemical shift perturbation between the two enzyme variants, or residues that are substantially sharper in the M^{pro}_{H41Q} variant due to modulation of conformational exchange processes in the active site such as R40, Q41, and I43.

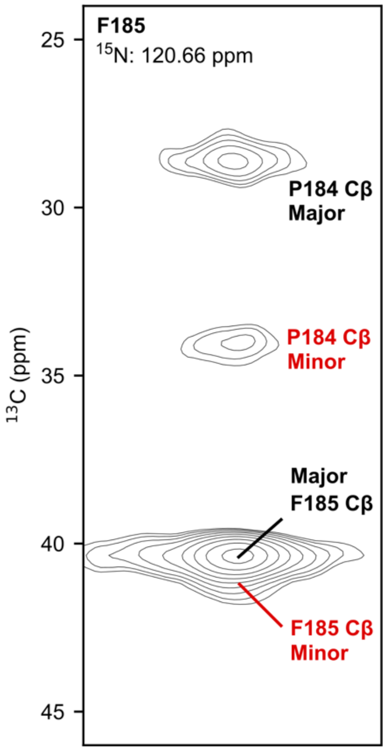

Figure S4: Cross section of the HNCB spectrum for residue F185, where the major and minor species are readily observable (exponential contour spacing separated by factors of 1.4). The difference in P184 C^{β} chemical shift is 5.5 ppm, which is demonstrative of cis-trans isomerism in solution. These two species are clearly in slow-exchange on the NMR timescale which is common for proline isomerism in proteins.

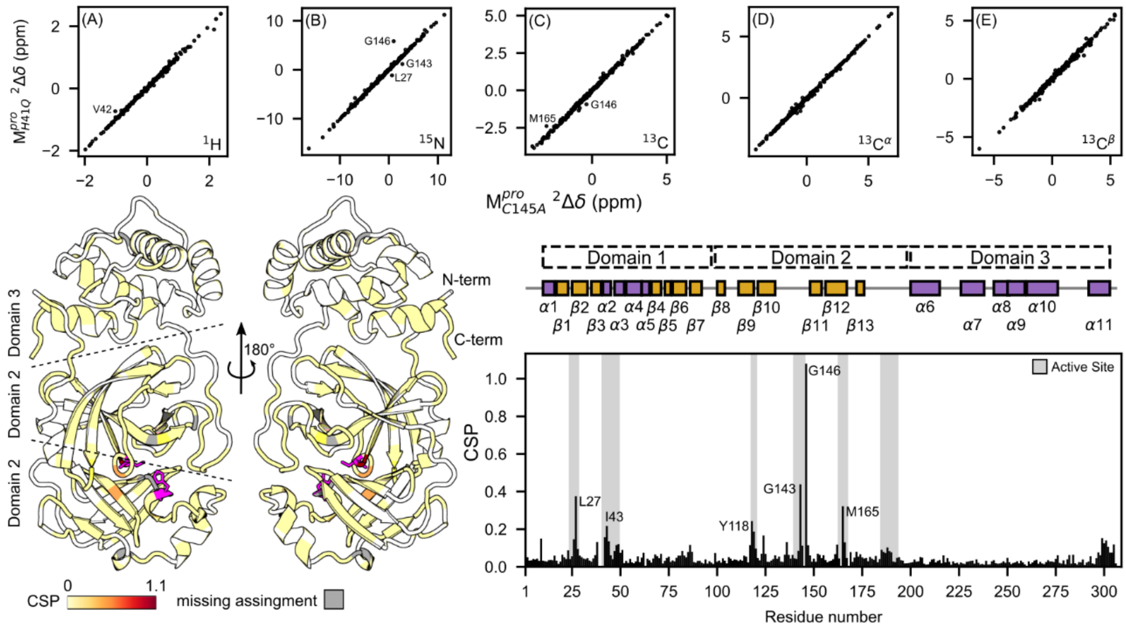

Figure S5: Comparison of chemical shift differences between the assignments of M^{pro}_{C145A} and M^{pro}_{H41Q} at 308K using the method of Williamson.[21] (A-E) Correlation plots of secondary-shift perturbations for backbone ^{1}H (A), ^{15}N (B), ^{13}C' (C), ^{13}C$^{\alpha}$ (D), ^{13}C$^{\beta}$ (E) nuclei. (F) Chemical shift perturbations (CSPs; see Methods section) were plotted on the X-ray structure of M^{pro} - PDB 5R8T - with missing residues colored in grey, and the catalytic dyad H41 and C145 shown in magenta. (G) A bar chart of CSP by residue with key residues annotated, and with active site regions shaded light blue. Secondary structure elements extracted from PDB 5R8T are annotated above the figure, with the three separate globular domains also indicated.

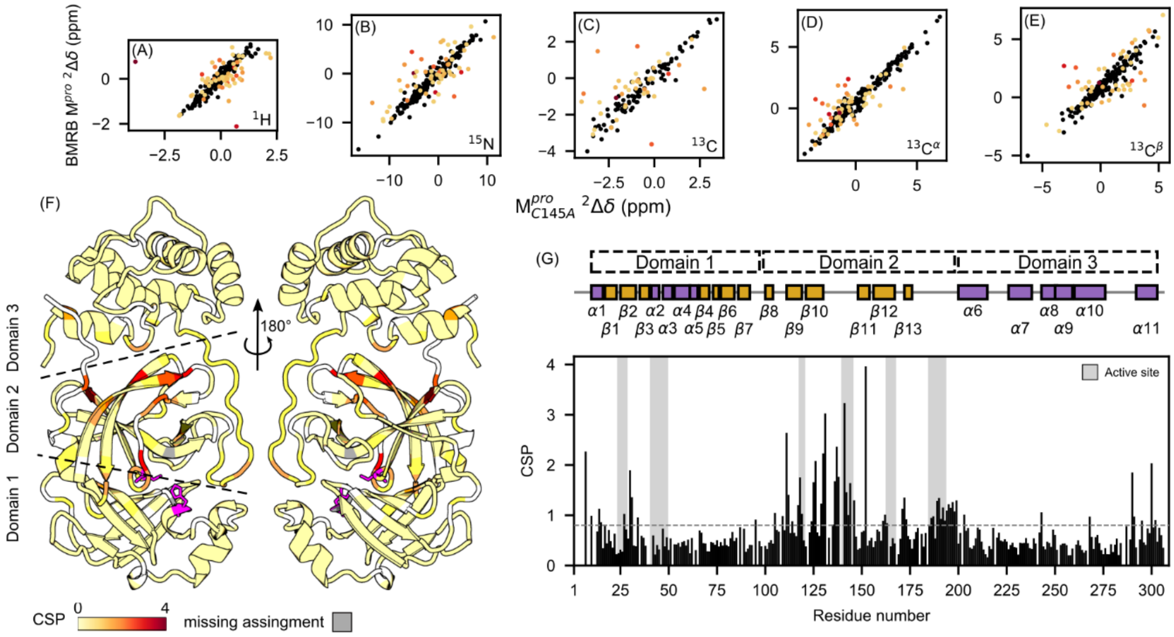

Figure S6: Comparison of chemical shift differences between the assignments of M^{pro}_{C145A} and BMRB deposition IDs 17251 (N-term; 308K) and 17911 (C-term; 298K). (A-E) Correlation plots of secondary-shift differences for backbone ^{1}H (A), ^{15}N (B), $^{13}C'$ (C), $^{13}C\alpha$ (D), $^{13}C\beta$ (E) nuclei. (F) Chemical shift differences (CSPs; see Methods section) plotted on the X-ray structure of M^{pro} - PDB 5R8T - with missing residues colored in grey, and the catalytic dyad H41 and C145 shown in magenta. Residues 1-199 were compared for assignments at 308K, residues 200-306 were compared to the M^{pro}_{C145A} assignment at 298K. Standard C^{α} and C^{β} deuterium isotope shifts were subtracted from M^{pro}_{C145A} resonances prior to comparison.[10] (G) A bar chart of CSP by residue with active site regions shaded light blue. Secondary structure elements extracted from PDB entry 5R8T are annotated above the figure, with the three separate globular domains also indicated. A dashed line at 0.8 indicates the threshold for coloring points in subplots A-E by magnitude of CSP (using the same color ramp as subplot F).

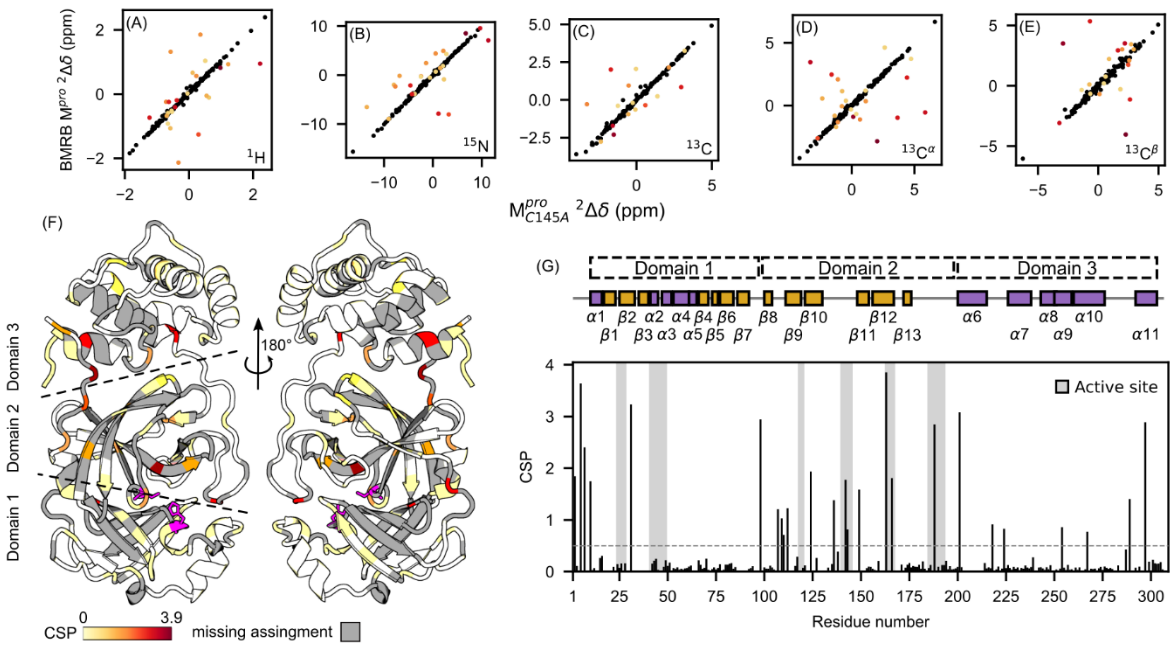

Figure S7: Comparison of chemical shift differences between the assignments of M^{pro}_{C145A} (10 mM NaPi pH 7.0, 0.5 mM TCEP, 0.3 mM DSS, 3% D_2O), and the partial M^{pro} assignment BMRB 50780 (50mM NaPi pH 6.8, 40mM NaCl, 0.1 mM EDTA, 3mM THP (Tris(hydroxypropyl)phosphine), 5% D_2O).[22] (A-E) Correlation plots of secondary shifts for backbone ^{1}H (A), ^{15}N (B), ^{13}C' (C), $^{13}C^{\alpha}$ (D), and $^{13}C^{\beta}$ (E) nuclei. Residues with chemical shift perturbations (CSPs; see Methods section) greater than 0.5 (grey dashed line) are colored according to (F) – and plotted on the X-ray structure of M^{pro} - PDB entry 5R8T with missing residues shown in grey, and the catalytic dyad H41 and C145 shown in magenta. Standard C^{α} and C^{β} deuterium isotope shifts were subtracted from M^{pro}_{C145A} resonances prior to comparison.[10] (G) A bar chart of CSP by residue with key residues annotated, and with active site regions shaded light blue. Secondary structure elements extracted from PDB entry 5R8T are annotated above the figure, with the three separate globular domains also indicated.

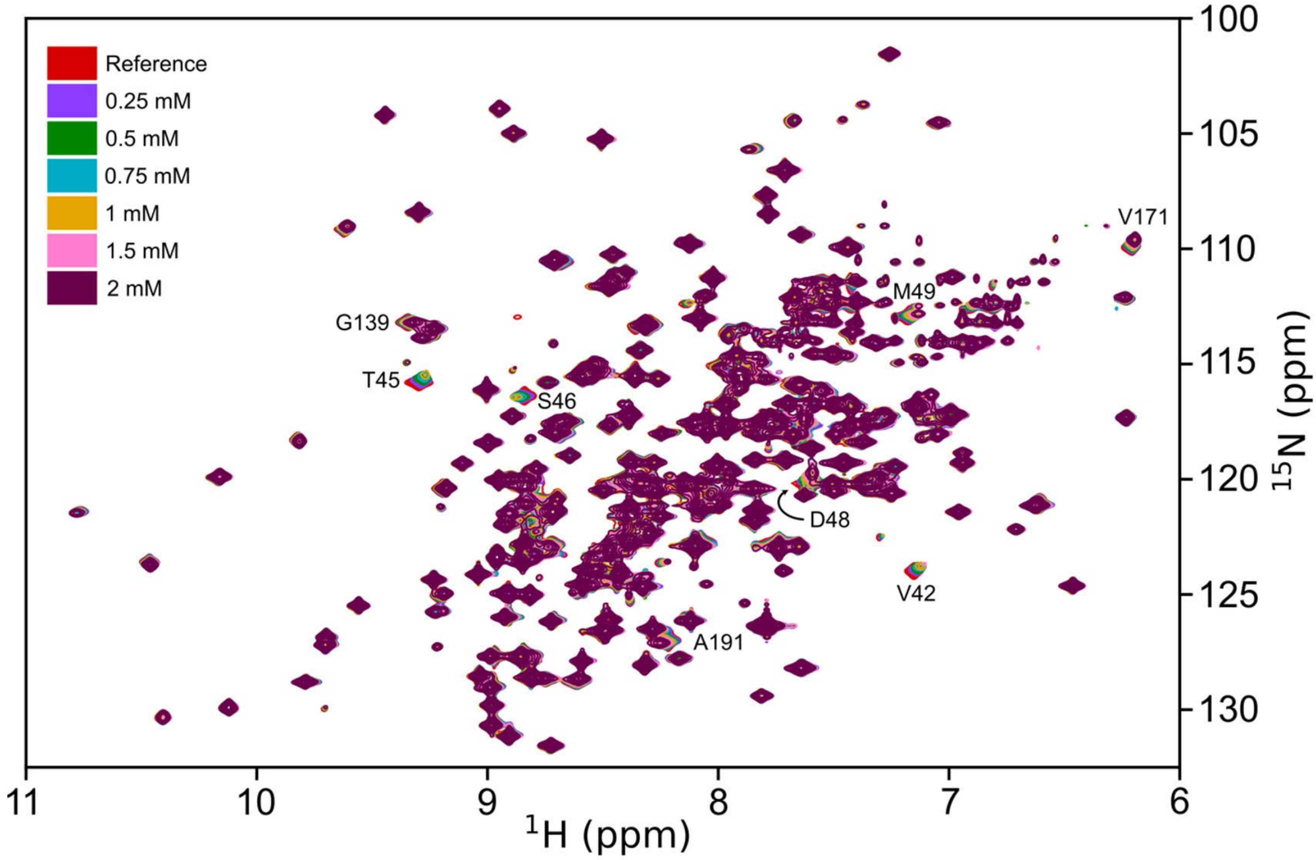

Figure S8: Titration of boceprevir (200 mM stock in d6-DMSO) into 0.2 mM (non-reprotonated) $^{2}H^{15}N$-labelled M^{pro}_{C145A} in 10 mM NaPi pH 7.0, 150 mM NaCl, 0.5 mM TCEP, 0.3 mM DSS, 3% D_2O at 900 MHz. Spectra colored according to boceprevir concentration indicated in the key.

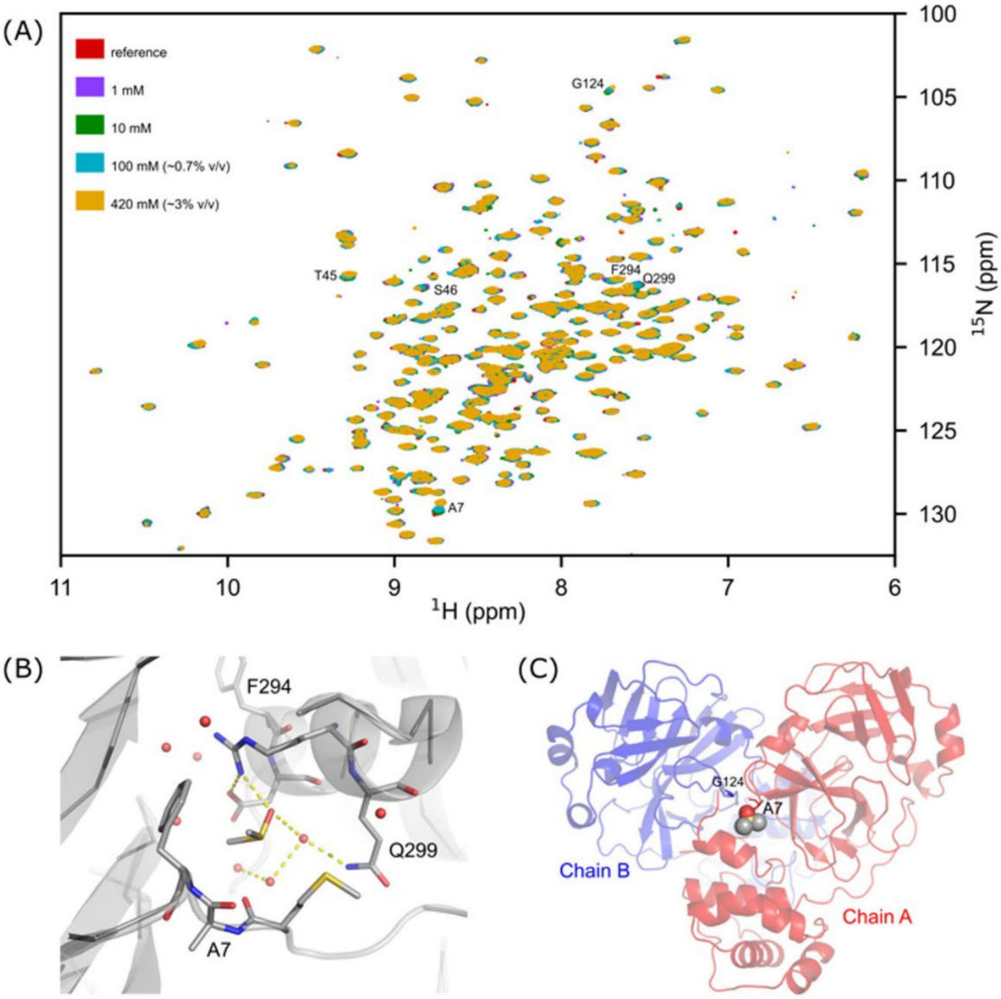

Figure S9: Characterization of protonated DMSO binding to (reprotonated) $^{2}H^{15}N$-M^{pro}_{C145A} in 10 mM NaPi pH 7.0, 150 mM NaCl, 1 mM TCEP, 3% D_2O. (A) A titration from 0-3% DMSO which is the usual range used and often assumed to be innocuous. (B) Illustration of a DMSO binding pocket from PDB: 5R8T, where proximity is illustrated between DMSO and residues with the largest perturbations in the TROSY spectrum (A7 and Q299). (C) Illustration of the DMSO binding site in context of the two monomer chains termed A (red) and B (blue), with the DMSO molecule illustrated as spheres for clarity.

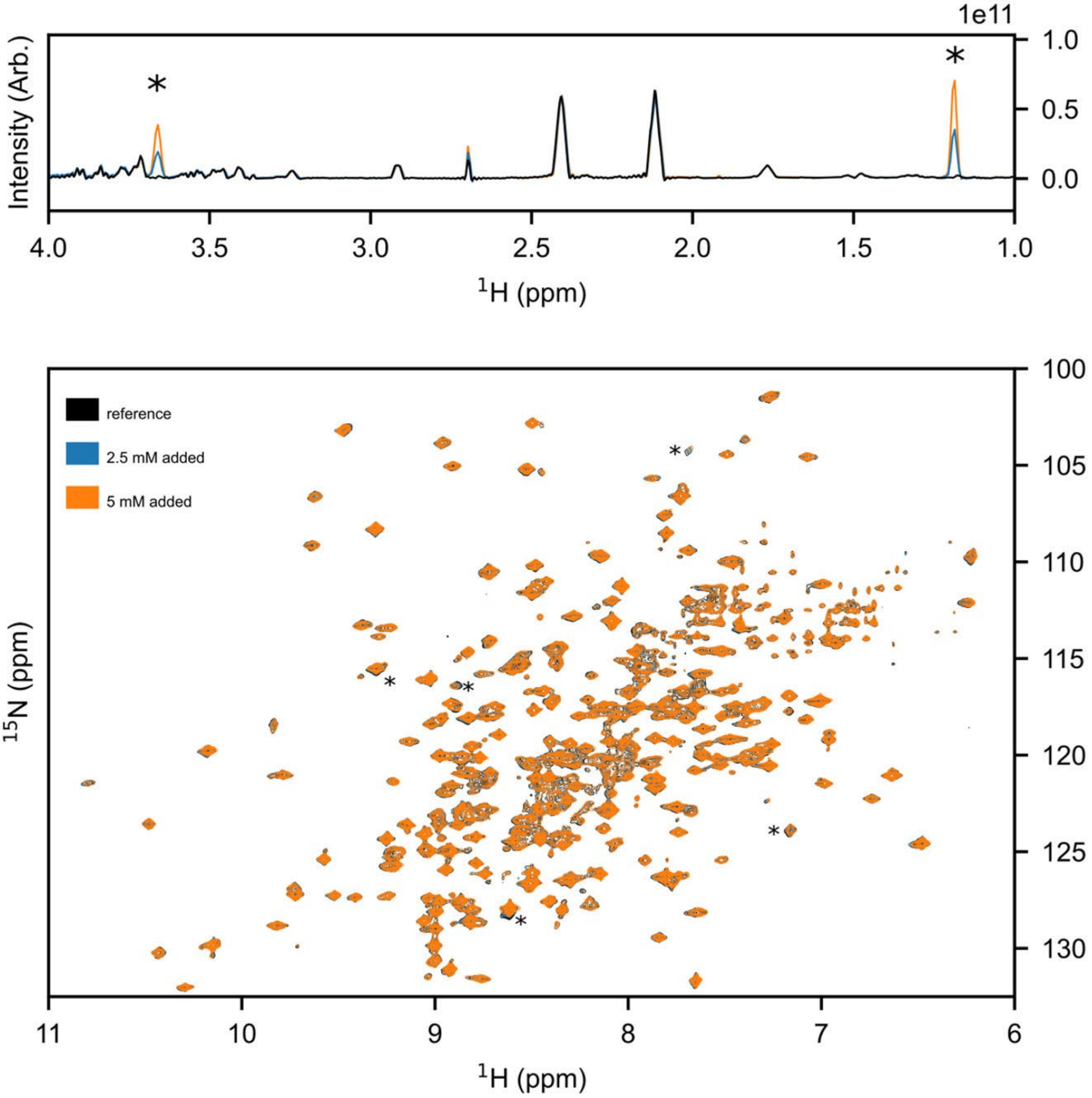

Figure S10: The absence of binding between ivermectin and (reprotonated) M^{pro}_{C145A} (10 mM NaPi pH 7.0, 150 mM NaCl, 1 mM TCEP, 3% D_2O). (Top) ^{1}H 1D spectra for: 0 mM (black), 2.5 mM (blue), and 5 mM (orange) added ivermectin, with peaks arising following the addition of ivermectin (200 mM stock in 100% d6-DMSO) indicated with an asterisk. (Bottom) ^{1}H^{15}N-TROSY spectra for the three titration points, the only peaks that show small perturbations are marked with an asterisk. All of these perturbations are due to interaction with DMSO, see SI Fig. S9.

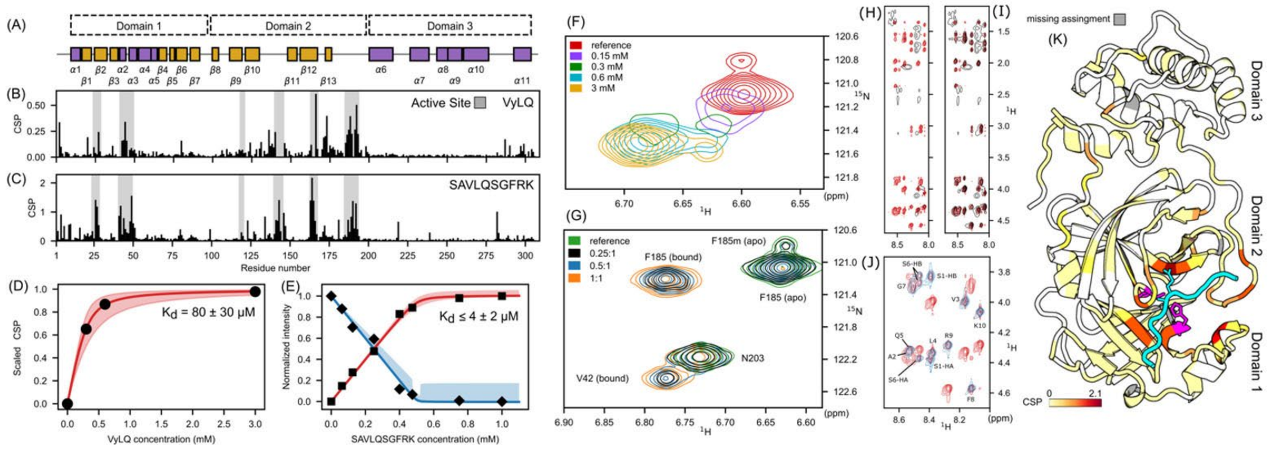

Figure S11: Binding of VyLQ and SAVLQSGFRK synthetic peptides to M^{pro}_{C145A}. (F, G) Titrations of (F) VyLQ and (G) SAVLQSGFRK into M^{pro}_{C145A}, followed by $^{1}H^{15}N$-TROSY-HSQC spectra (900 MHz, 25 ºC). (B, C) Combined chemical shift perturbation (CSP (ppm)) values were plotted by residue for points at the start and end of the titration (decapeptide CSP includes backbone ^{13}C shifts), with secondary structure and domain annotations derived from PDB 5R8T (A). Fits of the total CSP on VyLQ binding (D; 24 residues), and population change of bound (red) and unbound (blue) states on addition of SAVLQSGFRK (E; 17 residues) were used to determine K_d values (standard deviation of fit values reported as error). Selected regions of $^{1}H^{15}N$-TROSY-HSQC spectra from the VyLQ (F; 0.3 mM M^{pro}_{C145A}) and SAVLQSGFRK (G; 0.5 mM M^{pro}_{C145A}) titrations, with stoichiometries in (G) given as ligand:protein. (H-J) Small sections of 2D ^{1}H-^{1}H NOESY spectra of M^{pro}_{C145A}(0.5 mM):SAVLQSGFRK complex at (H) 20:19 stoichiometry, and (I) 1:2 stoichiometry (protein:substrate), NOESY spectra (τ_M = 200 ms) without (with) M^{pro}_{C145A} are colored red (black). (J) Assignment of the H^N-H^α TOCSY cross-peak region (blue) of SAVLQSGFRK in the absence of M^{pro}_{C145A}. (K) Magnitude of M^{pro}_{C145A} CSP observed on addition of SAVLQSGFRK, plotted as a color ramp (white-yellow-red) on the top ranked AlphaFold-Multimer prediction of the M^{pro}:SAVLQSGFRK complex.

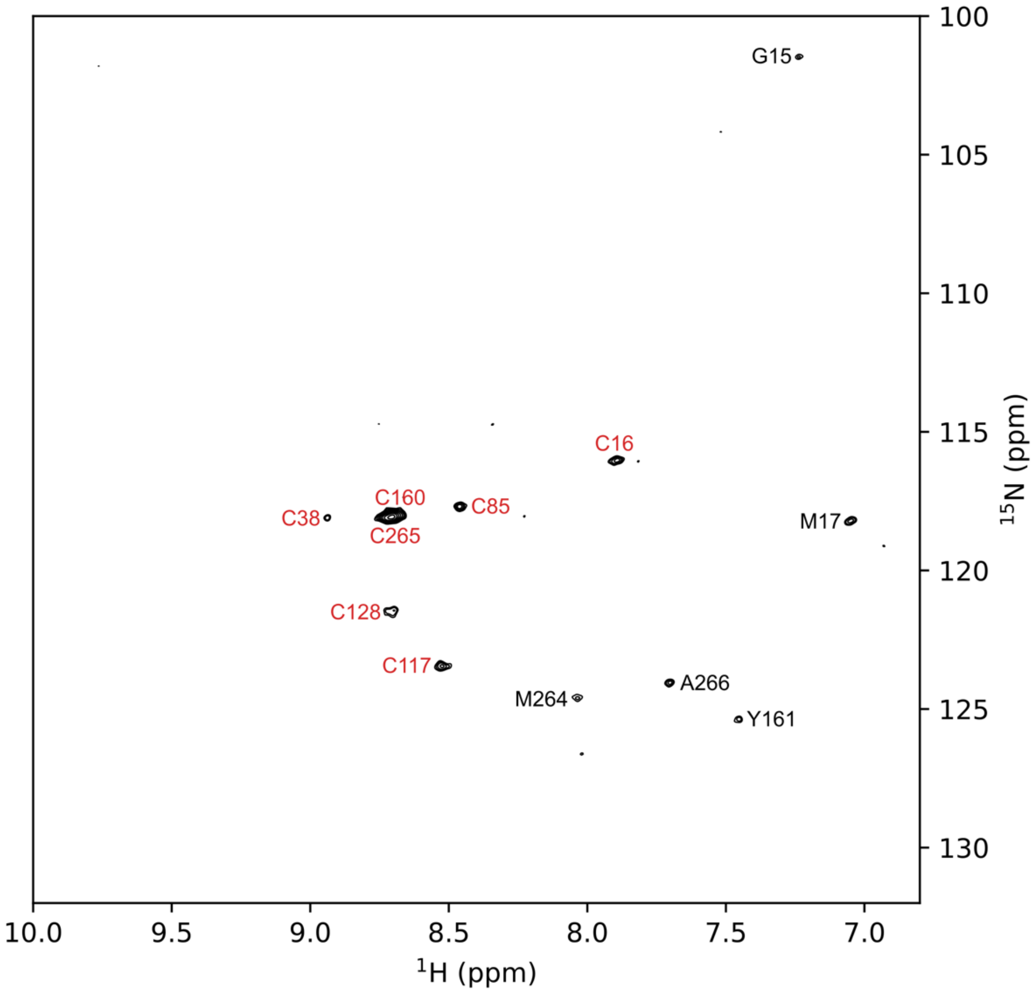

Figure S12: 800-MHz SILLY-NOESY TROSY spectrum recorded on (reprotonated) M^{pro}_{C145A} in the absence of ligand. The spectrum was recorded with a 70 ms NOE mixing period, with Cys residues annotated in red and their immediate neighbors labeled in black.

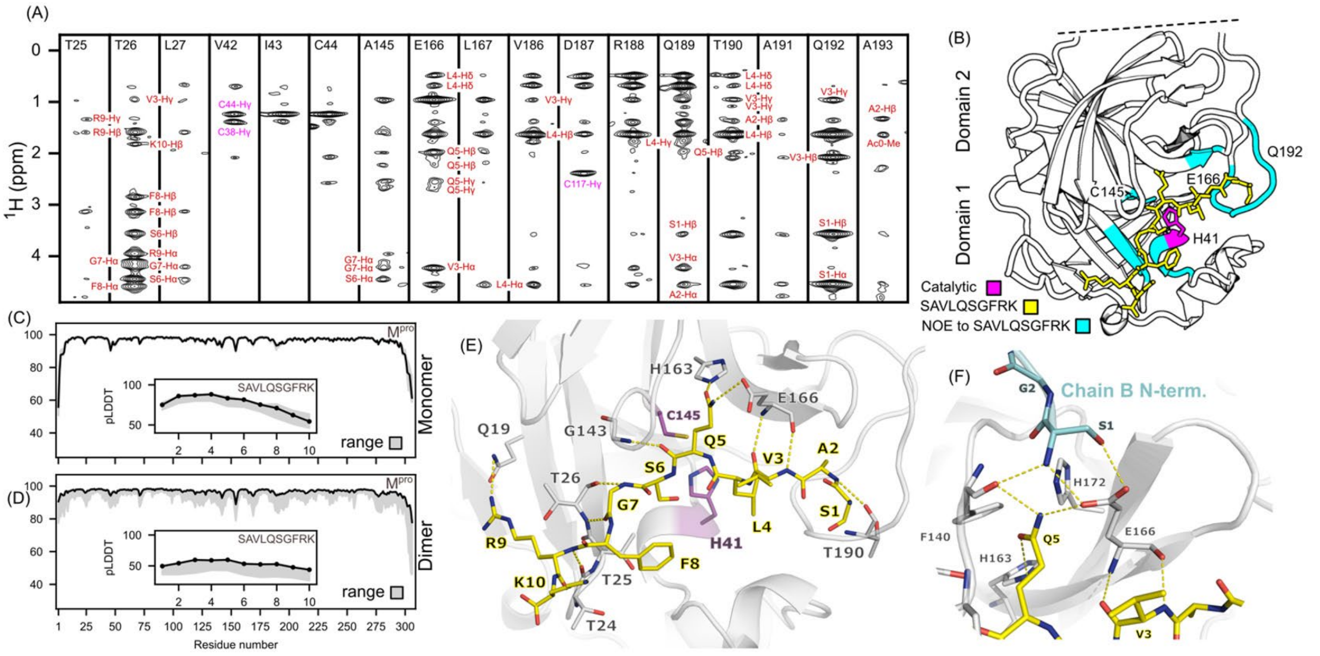

Figure S13: 3D-NOESY-TROSY spectra used to validate the AF-M predicted M^{pro}_{C145A}:SAVLQSGFRK binding pose. (A) Strips from the NOESY-TROSY spectrum for active site residues that have NOE (τ_M = 200 ms) cross peaks to the SAVLQSGFRK substrate (red annotations), or intra-/inter-residue NOEs to $^1H^{\gamma}$ atoms (magenta annotations). (B) Illustration of observed intermolecular NOEs (cyan) for residues of the top-ranked AF-M model of the (monomeric) M^{pro}:SAVLQSGFRK complex, with substrate residues in yellow and protein residues for which NOEs to peptide were observed in cyan. The H41/C145A catalytic dyad residues are shown as sticks and annotated. (C, D) AF-M pLDDT confidence scores for the models of a single SAVLQSGFRK peptide bound to a M^{pro} monomer (C), or M^{pro} homodimer (D), with the pLDDT scores for the peptide shown in the insets. pLDDT scores for the top ranked model (range) were colored black (shaded grey), with a substantial improvement in model confidence at the N- and C- termini for the homodimeric models. (E) Illustration of the H-bonding pattern in the active site of the top ranked AF-M model, with C145 and the scissile peptide bond in Van der Waals contact. (F) Illustration of the H-bonding pattern observed in the S1 binding pocket at the interface between the two monomers for the top-ranked AF-M model of the dimeric M^{pro}:SAVLQSGFRK complex.

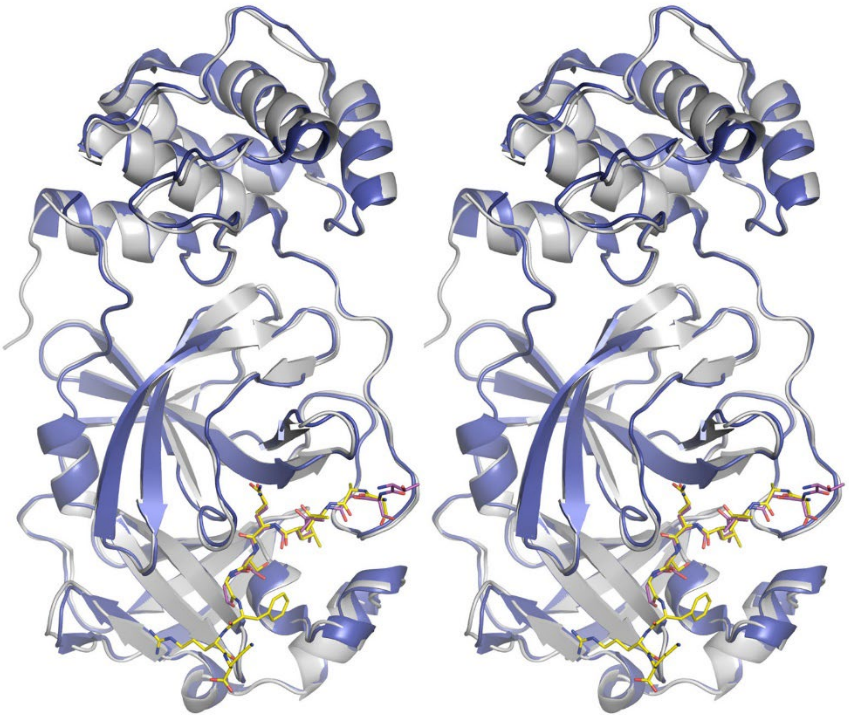

Figure S14: Cross-eye stereo comparison between the top-ranked AF-M model of the M^{pro}:SAVLQSGFRK complex (grey protein, yellow substrate), and PDB 2Q6G [23] of the M^{pro}:TSAVLQSGFRK complex (blue protein, magenta ligand) from 2010. Non-H atom RMSD for protein (0.50 Å) and substrate (1.15 Å) were determined using pymol (open-source v.2.4).

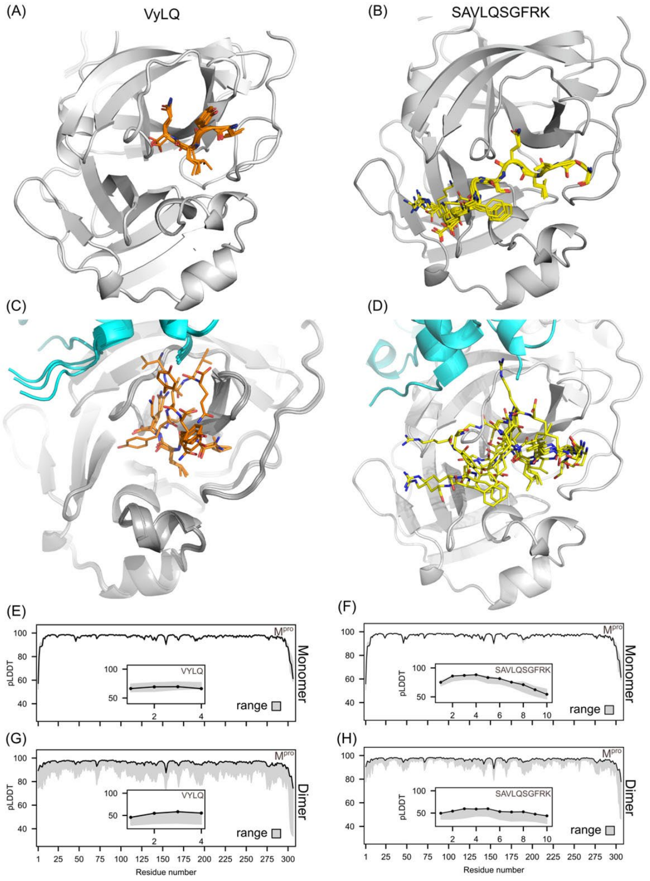

Figure S15: Overlay of structures predicted by AlphaFold-Multimer for monomeric (A, B) or dimeric (C, D) M^{pro} complexed with VyLQ (A, C) and SAVLQSGFRK (B, D). The substrate peptides are colored orange (VyLQ) or yellow (SAVLQSGFRK) with oxygen (red) and nitrogen (blue) atoms also colored, and for subfigures C and D, the companion monomer is colored cyan for clarity. The 5 AlphaFold-Multimer models in each figure were aligned (pymol 2.4), and all (non-hydrogen) peptide atoms drawn as sticks. (E-H) AlphaFold-Multimer, per-residue predicted local-distance difference test (pLDDT) confidence scores for the models of a single VyLQ peptide (E, G) or a single SAVLQSGFRK peptide (F, H) bound to a M^{pro} monomer (E, F), or M^{pro} homodimer (G, H), with the pLDDT scores for the peptide shown as insets. The top ranked model is in black, and the range of pLDDT scores is indicated with grey shading.

Figure S16 (overleaf): NOESY-TROSY strip plot of 0.5 mM (reprotonated) U-$^{2}H^{13}C^{15}N$-M^{pro}_{C145A} in the presence of 5 mM SAVLQSGFRK decapeptide in 10 mM NaPi pH 7.0, 0.5 mM TCEP, 0.3 mM DSS, 3% D_2O, recorded at 800 MHz. NUS data (40% sampled) acquired using a 15-ms indirect ^{1}H chemical shift evolution, 60 ms of ^{15}N chemical shift evolution, and a 200-ms NOESY mixing period (see **Table S6** for details). The spectrum was reconstructed using SMILE[24] with 50% extension of indirect dimensions. NOESY peaks were automatically picked and curated using Sparky and used to both aid the automated assignment of the M^{pro}_{C145A}:SAVLQSGFRK complex, and validate assignments manually using NMRDraw and the *scroll.tcl* macro. Missing assignments are annotated.

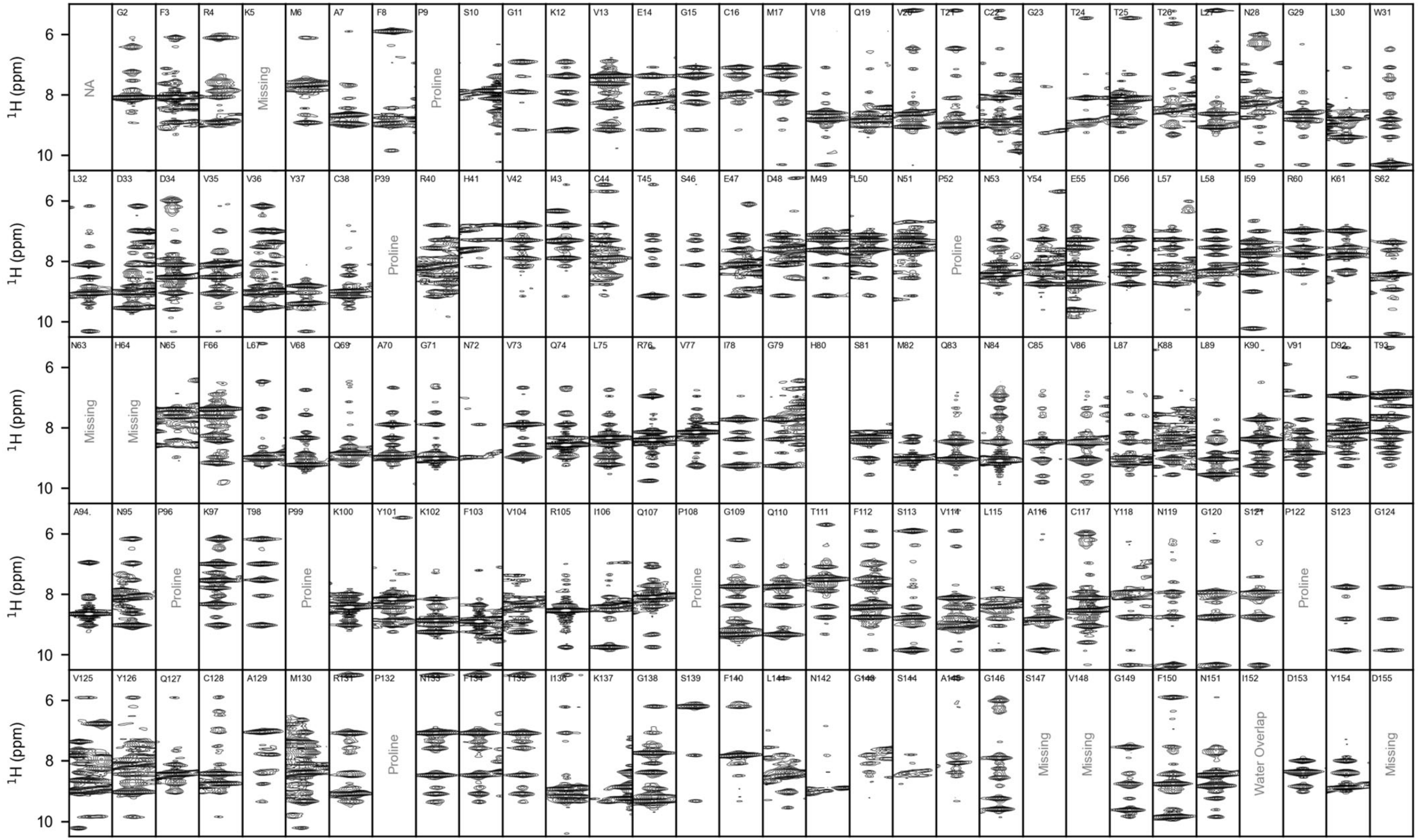

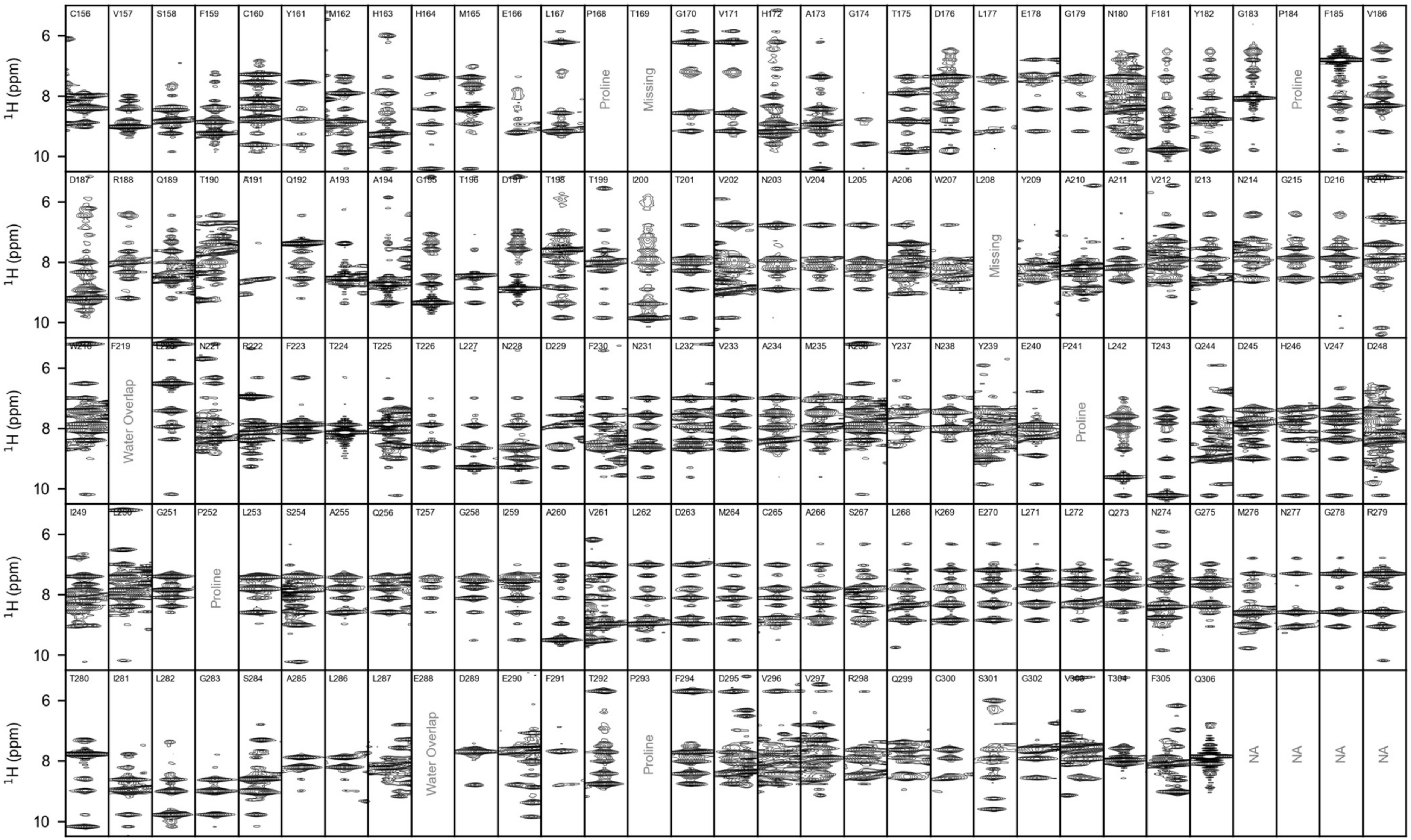
1H (ppm)
6
8
10
C156 V157 S158 F159 C160 Y161 M162 H163 H164 M165 E166 L167 P168 T169 G170 V171 H172 A173 G174 T175 D176 L177 E178 G179 N180 F181 Y182 G183 P184 F185 V186
Proline
Missing
Proline
D187 R188 Q189 T190 A191 Q192 A193 A194 G195 T196 D197 T198 T199 I200 T201 V202 N203 V204 L205 A206 W207 L208 Y209 A210 A211 V212 I213 N214 G215 D216
Missing
F219 N221 R222 F223 T224 T225 T226 L227 N228 D229 F230 N231 L232 V233 A234 M235 Y237 N238 Y239 E240 P241 L242 T243 Q244 D245 H246 V247 D248
Water Overlap
Proline
I249 G251 P252 L253 S254 A255 Q256 T257 G258 I259 A260 V261 L262 D263 M264 C265 A266 S267 L268 K269 E270 L271 L272 Q273 N274 G275 M276 N277 G278 R279
Proline
T280 I281 L282 G283 S284 A285 L286 L287 E288 D289 E290 F291 T292 P293 F294 D295 V296 V297 R298 Q299 C300 S301 G302 T304 F305 Q306
Water Overlap
Proline
NA
NA
NA
NA

Figure S17 (overleaf): The same spectrum as SI Fig. S16, but with the aliphatic region shown to highlight the utility of Cys-$^{1}H^{\gamma}$ observation on a perdeuterated protein background.

SUPPORTING INFORMATION

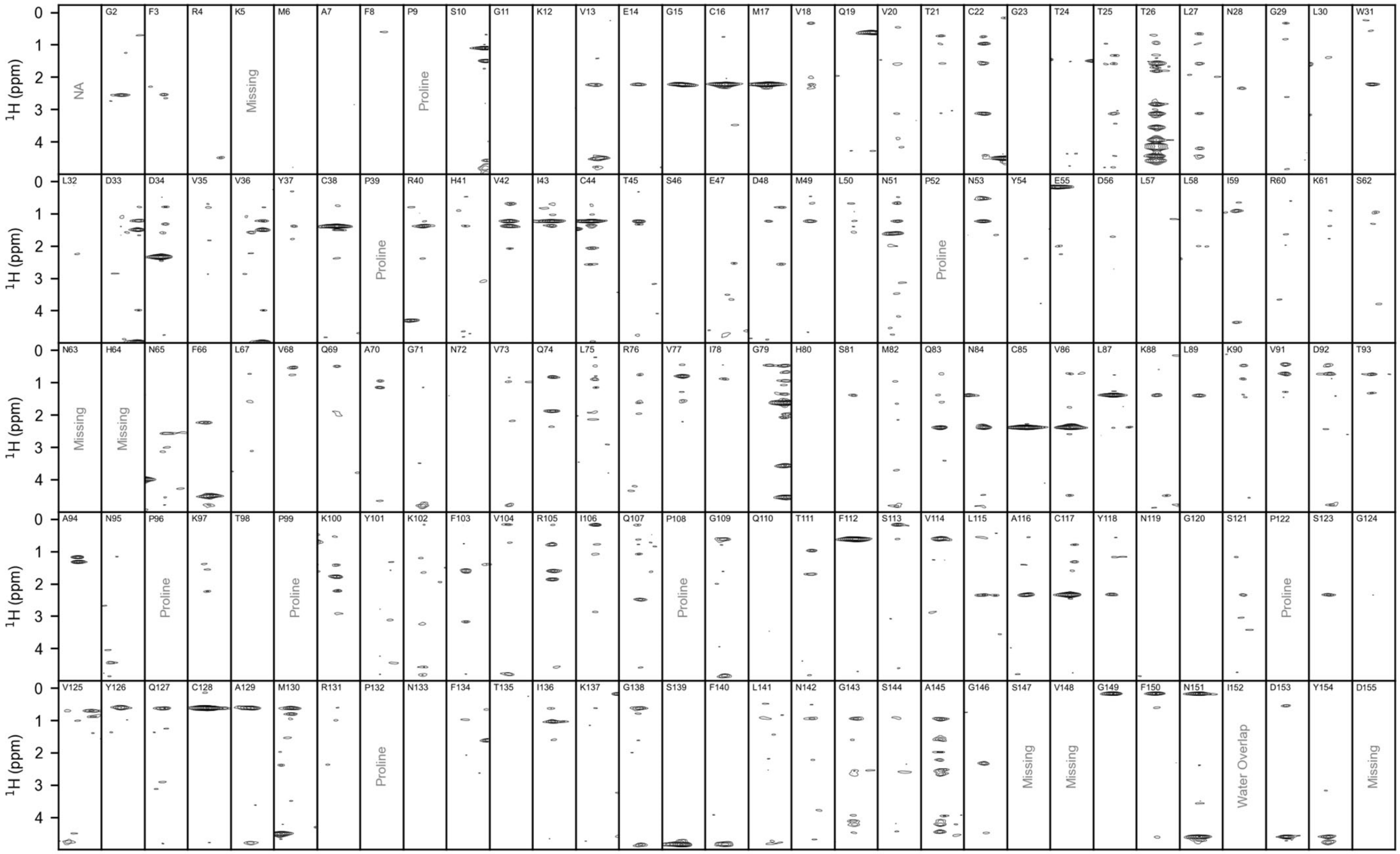

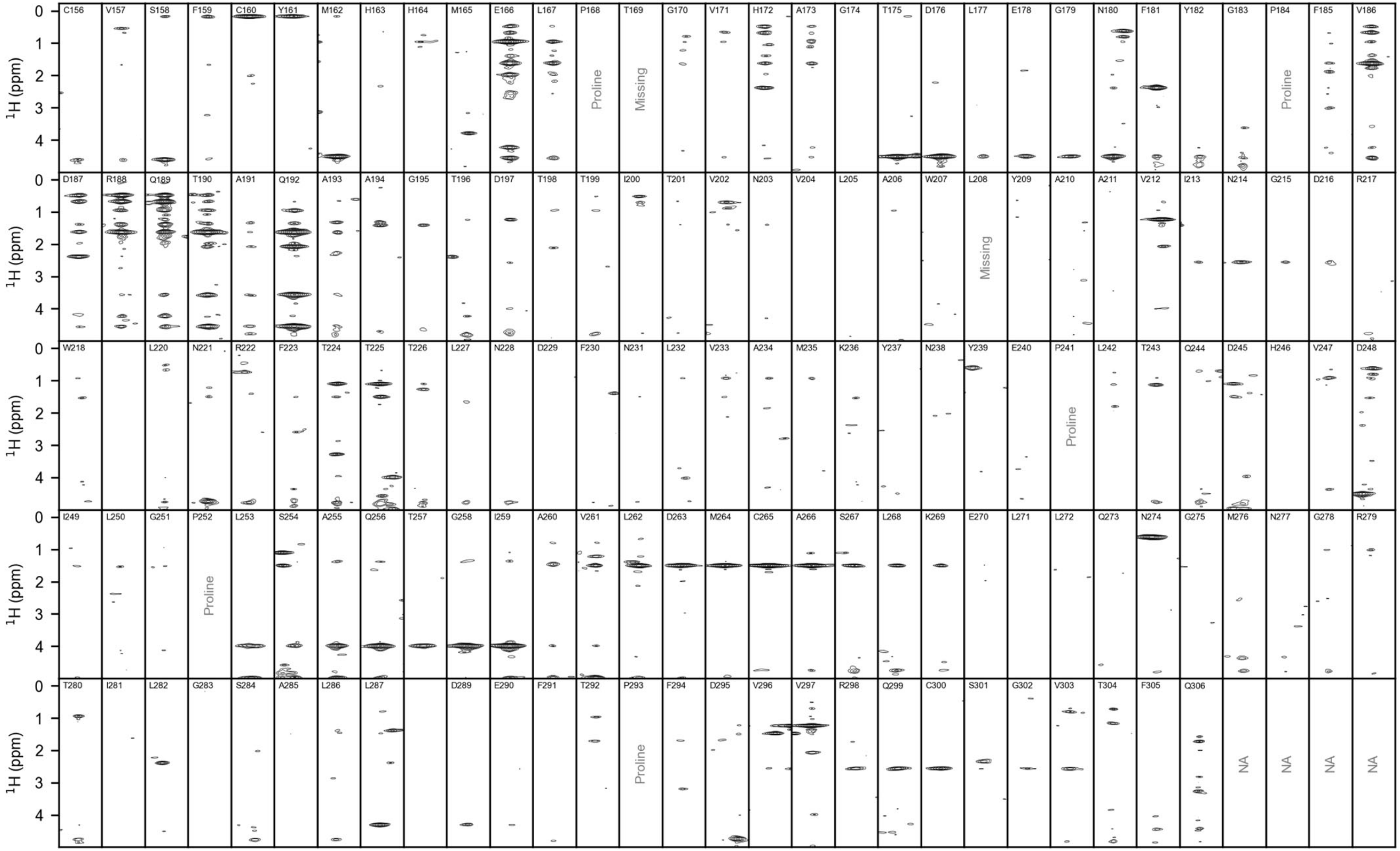
C156 V157 S158 F159 C160 Y161 M162 H163 H164 M165 E166 L167 P168 T169 G170 V171 H172 A173 G174 T175 D176 L177 E178 G179 N180 F181 Y182 G183 P184 F185 V186
D187 R188 Q189 T190 A191 Q192 A193 A194 G195 T196 D197 T198 T199 I200 T201 V202 N203 V204 L205 A206 W207 L208 Y209 A210 A211 V212 I213 N214 G215 D216 R217
W218 L220 N221 R222 F223 T224 T225 T226 L227 N228 D229 F230 N231 L232 V233 A234 M235 K236 Y237 N238 Y239 E240 P241 L242 T243 Q244 D245 H246 V247 D248
I249 L250 G251 P252 L253 S254 A255 Q256 T257 G258 I259 A260 V261 L262 D263 M264 C265 A266 S267 L268 K269 E270 L271 L272 Q273 N274 G275 M276 N277 G278 R279
T280 I281 L282 G283 S284 A285 L286 L287 D289 E290 F291 T292 P293 F294 D295 V296 V297 R298 Q299 C300 S301 G302 V303 T304 F305 Q306
Proline
Missing
NA
^{1}H (ppm)
0 1 2 3 4

Figure S18 (overleaf): 900 MHz HNCA strip plot of 1.1 mM (reprotonated) U-$^{2}H^{13}C^{15}N$-M^{pro}_{C145A} in 10 mM NaPi pH 7.0, 0.5 mM TCEP, 0.3 mM DSS, 3% D_2O. NUS data (32% sampled) were acquired using 38 ms indirect $^{13}C^{\alpha}$ chemical shift evolution (States-TPPI) and a 50 ms ^{15}N chemical shift evolution (Echo-AntiEcho), with 2 transients per increment, and a TROSY readout. $^{13}C^{\alpha}$ non-uniform sampling comprised fully (randomly) sampling data points up to 7ms (38ms), with ^{2}H decoupling achieved using a WALTZ16 scheme.[25] The spectrum was reconstructed using SMILE[24] with 50% extension of the indirect dimensions, employing virtual decoupling of the $^{1}J_{C\alpha C\beta}$ couplings, using a similar approach to the method published by Kazimierczuk *et. al* [26]. HNCA peaks were automatically picked and curated using Sparky and used to both aid the automated assignment of the apo-M^{pro}_{C145A} complex and validate assignments manually in NMRDraw using the *scroll.tcl* macro. Missing assignments are annotated.

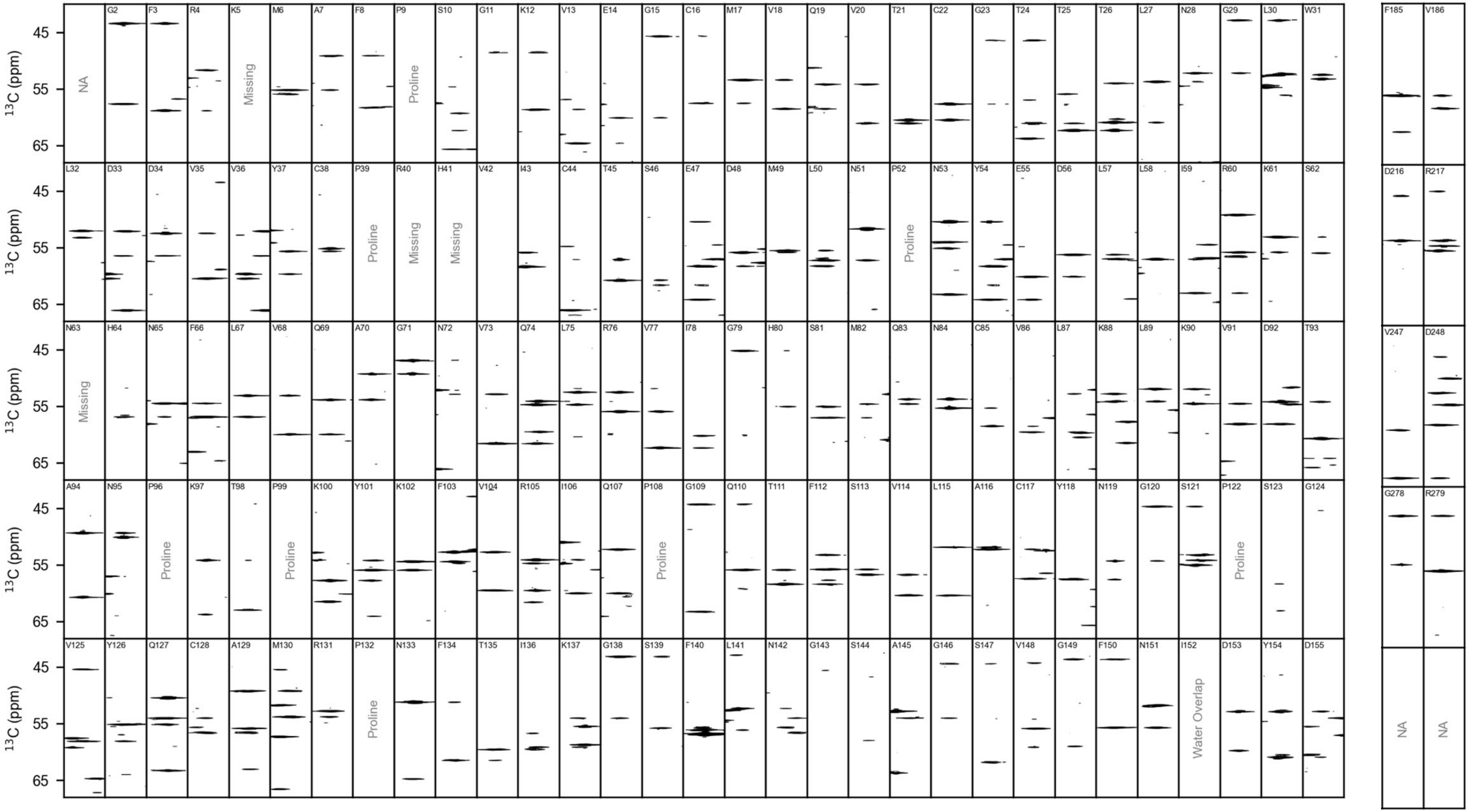
13C (ppm)
45
55
65
G2 F3 R4 K5 M6 A7 F8 P9 S10 G11 K12 V13 E14 G15 C16 M17 V18 Q19 V20 T21 C22 G23 T24 T25 T26 L27 N28 G29 L30 W31
NA
Missing
Proline
F185 V186
L32 D33 D34 V35 V36 Y37 C38 P39 R40 H41 V42 I43 C44 T45 S46 E47 D48 M49 L50 N51 P52 N53 Y54 E55 D56 L57 L58 I59 R60 K61 S62
Proline
Missing
Missing
Proline
D216 R217
N63 H64 N65 F66 L67 V68 Q69 A70 G71 N72 V73 Q74 L75 R76 V77 I78 G79 H80 S81 M82 Q83 N84 C85 V86 L87 K88 L89 K90 V91 D92 T93
Missing
V247 D248
A94 N95 P96 K97 T98 P99 K100 Y101 K102 F103 V104 R105 I106 Q107 P108 G109 Q110 T111 F112 S113 V114 L115 A116 C117 Y118 N119 G120 S121 P122 S123 G124
Proline
Proline
Proline
Proline
G278 R279
V125 Y126 Q127 C128 A129 M130 R131 P132 N133 F134 T135 I136 K137 G138 S139 F140 L141 N142 G143 S144 A145 G146 S147 V148 G149 F150 N151 I152 D153 Y154 D155
Proline
Water Overlap
NA
NA

Table S1: M^{pro}_{C145A} assignment (298K)

Res. Number	Res. Type	H	N	C'	Cα	Cβ
1	SER	-	-	171.594	57.232	62.340
2	GLY	8.151	110.462	172.761	43.121	-
3	PHE	7.997	119.346	174.622	58.464	41.133
4	ARG	8.839	127.708	175.087	51.309	34.165
5	LYS	-	-	175.025	55.559	27.918
6	MET	7.626	128.097	174.180	54.804	34.253
7	ALA	8.598	128.674	177.637	48.787	19.923
8	PHE	8.835	120.274	171.853	57.926	37.213
9	PRO	-	-	178.584	61.934	28.182
10	SER	7.875	114.903	177.404	58.894	64.926
11	GLY	10.759	121.325	176.190	48.209	-
12	LYS	9.095	119.227	178.335	58.217	31.941
13	VAL	7.295	117.440	178.265	64.115	30.301
14	GLU	8.274	121.079	179.022	59.667	29.301
15	GLY	7.250	101.476	173.969	45.345	-
16	CYS	7.905	115.970	173.652	57.145	28.447
17	MET	7.051	118.033	177.107	53.028	27.648
18	VAL	8.800	114.668	174.026	58.077	33.674
19	GLN	8.689	120.781	174.495	53.771	30.239
20	VAL	8.760	125.427	173.722	60.646	32.493
21	THR	9.119	123.522	173.844	60.094	70.308
22	CYS	8.835	127.483	174.934	57.273	26.660
23	GLY	9.177	120.267	174.654	46.088	-
24	THR	8.676	117.928	174.500	60.685	68.027
25	THR	8.066	122.642	172.200	61.932	68.673
26	THR	8.528	123.895	173.521	60.536	70.012
27	LEU	9.189	125.212	174.096	53.352	39.304
28	ASN	7.780	115.653	172.898	51.860	40.856
29	GLY	8.464	102.710	170.156	42.547	-
30	LEU	8.737	122.839	174.222	52.135	44.026
31	TRP	10.266	131.948	174.445	52.877	29.431
32	LEU	9.000	127.236	177.011	51.744	43.137
33	ASP	8.911	124.836	174.812	56.126	38.119
34	ASP	8.460	123.164	173.229	52.138	37.334
35	VAL	8.061	119.176	173.808	60.009	33.781
36	VAL	8.987	124.866	174.565	59.236	31.541
37	TYR	9.389	127.289	176.347	55.278	38.617
38	CYS	8.936	118.023	169.891	54.797	28.366
39	PRO	-	-	-	-	-
40	ARG	-	-	-	-	-
41	HIS	-	-	175.852	-	-
42	VAL	7.133	123.899	174.223	62.941	29.988
43	ILE	7.373	109.843	175.038	-	-
44	CYS	7.812	120.360	176.469	56.722	28.806
45	THR	9.287	115.698	176.334	60.377	70.350
46	SER	8.827	116.257	177.068	61.207	70.242
47	GLU	8.068	120.285	178.672	57.871	27.756
48	ASP	7.598	120.070	176.857	55.510	40.465
49	MET	7.166	112.857	177.207	55.142	29.801
50	LEU	7.288	119.805	177.834	56.879	39.856
51	ASN	7.531	112.164	170.948	51.396	36.716
52	PRO	-	-	176.478	62.907	29.701
53	ASN	8.361	122.562	175.430	50.133	35.583
54	TYR	8.080	120.330	177.241	63.765	37.432
55	GLU	8.642	117.390	178.863	59.722	27.227
56	ASP	7.227	119.332	178.187	55.901	40.674
57	LEU	8.235	117.945	180.420	56.644	40.588
58	LEU	8.276	119.264	179.723	56.670	39.653
59	ILE	7.412	117.518	176.953	62.594	36.222
60	ARG	6.978	117.129	176.517	55.420	28.547
61	LYS	7.725	119.182	174.999	52.746	31.471
62	SER	8.344	115.265	174.981	55.579	64.273
63	ASN	-	-	-	56.167	-
64	HIS	7.517	111.715	176.337	56.495	28.453
65	ASN	7.588	116.670	173.236	54.148	38.372
66	PHE	7.356	117.585	173.158	56.485	39.391
67	LEU	8.903	125.893	175.809	52.713	40.789
68	VAL	9.031	128.498	174.408	59.512	30.904
69	GLN	8.706	126.038	173.501	53.441	30.960
70	ALA	8.894	131.033	177.496	48.896	18.727
71	GLY	8.993	116.060	174.660	46.540	-
72	ASN	8.807	124.927	174.715	52.457	37.097
73	VAL	7.833	121.741	174.453	61.125	31.881
74	GLN	8.490	126.590	175.694	54.309	27.317
75	LEU	8.274	126.501	175.713	52.131	40.502
76	ARG	8.304	124.560	175.067	55.510	29.121
77	VAL	8.111	126.062	176.599	61.872	29.848

78	ILE	9.189	121.242	175.983	59.777	37.085
79	GLY	7.666	112.042	171.069	44.832	-
80	HIS	8.200	115.657	172.730	54.681	31.231
81	SER	8.261	112.933	171.111	56.622	64.110
82	MET	8.950	123.390	174.262	54.192	34.470
83	GLN	8.974	129.762	173.022	53.390	28.716
84	ASN	9.028	124.001	174.992	54.990	36.253
85	CYS	8.459	117.593	174.179	58.104	26.659
86	VAL	8.372	119.154	173.733	59.113	33.600
87	LEU	9.026	124.772	174.261	52.413	42.353
88	LYS	8.443	121.077	175.981	53.767	30.476
89	LEU	9.495	127.265	175.125	51.584	39.229
90	LYS	8.345	127.515	176.473	54.133	31.026
91	VAL	8.744	120.078	176.118	57.715	33.610
92	ASP	8.102	117.456	175.391	53.869	39.710
93	THR	6.897	114.138	171.935	60.294	71.405
94	ALA	8.580	127.798	177.785	48.967	17.357
95	ASN	7.982	121.016	176.656	49.783	33.391
96	PRO	-	-	177.007	63.295	30.078
97	LYS	7.482	118.005	175.584	53.777	29.080
98	THR	6.933	118.767	172.379	62.555	69.864
99	PRO	-	-	176.186	61.043	30.470
100	LYS	8.377	121.199	176.744	57.336	29.892
101	TYR	8.094	122.856	173.946	55.523	41.467
102	LYS	8.809	118.194	174.467	54.020	34.909
103	PHE	8.833	122.734	175.698	52.341	35.931
104	VAL	8.268	120.448	173.467	59.064	34.215

105	ARG	8.473	126.485	177.440	53.696	29.050
106	ILE	8.350	124.329	173.964	59.566	38.618
107	GLN	8.034	117.815	173.577	51.843	27.888
108	PRO	-	-	176.880	62.785	30.901
109	GLY	9.210	113.399	173.565	43.933	-
110	GLN	7.665	117.988	174.918	55.457	28.142
111	THR	7.428	109.868	175.052	58.002	71.033
112	PHE	8.338	114.349	173.013	55.403	37.377
113	SER	8.706	113.978	171.780	56.293	63.523
114	VAL	8.957	123.020	174.608	59.923	32.391
115	LEU	8.263	126.319	175.386	51.474	41.482
116	ALA	8.719	131.489	176.614	51.839	17.172
117	CYS	8.532	123.284	170.342	57.098	30.377
118	TYR	7.974	115.003	176.683	57.156	40.317
119	ASN	10.455	123.474	175.117	53.929	36.568
120	GLY	8.875	104.952	172.687	44.317	-
121	SER	7.905	115.293	172.606	54.630	63.307
122	PRO	-	-	176.111	62.661	31.525
123	SER	9.814	118.255	175.657	57.940	64.570
124	GLY	7.676	104.422	169.983	45.012	-
125	VAL	8.892	119.883	174.254	57.662	34.609
126	TYR	8.022	120.534	171.579	54.750	38.153
127	GLN	8.375	122.535	175.312	53.605	28.658
128	CYS	8.700	121.284	170.656	56.179	29.424
129	ALA	6.987	117.154	176.657	48.848	19.265
130	MET	8.282	120.115	175.031	53.420	28.541
131	ARG	8.965	128.906	177.047	52.376	24.971

132	PRO	-	-	175.817	64.390	31.341
133	ASN	6.980	111.122	175.385	50.894	35.685
134	PHE	8.394	110.940	172.560	61.078	33.748
135	THR	7.041	104.459	172.830	59.165	71.821
136	ILE	8.683	110.518	175.440	58.717	39.564
137	LYS	9.207	125.678	176.843	53.615	28.536
138	GLY	9.334	113.111	172.523	42.804	-
139	SER	6.225	112.080	173.656	55.377	-
140	PHE	7.794	126.293	174.252	55.736	42.793
141	LEU	8.709	122.757	178.237	51.939	-
142	ASN	8.754	121.379	175.656	55.336	37.137
143	GLY	8.898	113.831	174.476	45.221	-
144	SER	8.131	112.251	177.656	57.563	-
145	ALA	8.739	128.589	177.006	53.605	17.490
146	GLY	9.266	113.792	172.988	44.068	-
147	SER	7.880	113.339	173.483	61.409	62.594
148	VAL	7.678	118.074	174.864	58.621	35.451
149	GLY	9.591	106.473	174.634	43.284	-
150	PHE	9.758	120.870	171.915	55.343	41.024
151	ASN	8.408	116.640	173.359	51.478	42.140
152	ILE	3.344	117.003	173.777	59.412	36.348
153	ASP	8.304	127.942	175.894	52.525	41.132
154	TYR	8.835	123.459	173.394	60.517	34.615
155	ASP	8.358	121.540	174.735	52.495	39.781
156	CYS	7.941	119.547	173.974	56.365	27.783
157	VAL	8.964	130.554	174.313	61.300	30.316
158	SER	8.764	124.151	175.354	55.091	61.779

159	PHE	9.177	125.653	175.822	58.334	38.982
160	CYS	8.691	117.523	172.916	54.438	32.149
161	TYR	7.488	125.369	172.067	56.674	42.149
162	MET	8.780	127.855	173.814	53.193	34.159
163	HIS	9.210	127.195	176.617	57.461	31.492
164	HIS	7.629	122.673	-	54.374	-
165	MET	-	-	172.960	53.961	34.291
166	GLU	8.801	122.894	175.852	54.305	30.373
167	LEU	8.960	127.608	176.602	52.702	38.458
168	PRO	-	-	-	-	-
169	THR	-	-	175.496	61.286	-
170	GLY	8.449	110.175	173.163	44.818	-
171	VAL	6.198	109.810	174.031	58.620	30.767
172	HIS	8.763	120.921	172.727	58.854	33.392
173	ALA	8.880	121.476	177.523	49.170	22.394
174	GLY	9.609	109.062	170.974	44.991	-
175	THR	7.834	105.567	174.869	57.831	72.277
176	ASP	7.262	117.182	178.346	52.539	39.072
177	LEU	9.185	124.943	176.375	52.024	33.711
178	GLU	7.300	114.075	176.371	54.871	27.934
179	GLY	7.453	104.348	173.808	45.139	-
180	ASN	8.325	120.086	176.485	51.404	37.520
181	PHE	9.688	127.103	178.758	60.122	36.876
182	TYR	8.792	123.112	175.376	59.042	36.872
183	GLY	8.008	111.175	172.382	43.666	-
184	PRO	-	-	175.295	61.955	27.738
185	PHE	6.600	121.028	172.767	55.632	39.527
186	VAL	8.435	111.228	175.013	57.828	33.821
187	ASP	8.798	120.816	174.567	50.956	35.024
188	ARG	7.714	117.565	174.642	53.722	31.435
189	GLN	8.434	122.826	174.064	54.374	26.258
190	THR	7.631	117.594	173.381	58.858	68.939
191	ALA	8.205	126.926	176.882	52.361	16.052
192	GLN	7.730	122.672	173.853	54.332	30.077
193	ALA	8.428	124.252	176.472	50.570	19.310
194	ALA	8.571	124.486	178.427	50.372	19.825
195	GLY	9.284	108.358	173.857	43.052	-
196	THR	8.376	117.153	174.257	63.306	68.032
197	ASP	8.803	128.520	175.581	52.469	40.958
198	THR	7.479	114.536	173.064	59.380	70.314
199	THR	7.964	116.638	174.389	62.015	67.741
200	ILE	9.780	128.716	176.050	60.219	34.832
201	THR	7.805	129.341	175.984	66.696	65.529
202	VAL	8.827	119.966	174.457	64.232	29.045
203	ASN	6.702	122.137	179.530	53.669	35.990
204	VAL	7.881	125.370	178.804	65.511	29.400
205	LEU	8.172	117.013	178.073	57.482	40.400
206	ALA	8.056	120.685	178.189	55.117	16.628
207	TRP	8.415	121.530	176.747	60.110	27.634
208	LEU	8.518	120.104	180.709	56.872	-
209	TYR	8.151	117.828	178.406	63.646	36.982
210	ALA	8.085	122.765	180.021	53.792	15.119
211	ALA	8.096	123.728	179.313	54.376	16.028
212	VAL	7.862	120.408	181.239	65.563	29.741
213	ILE	8.533	124.561	177.369	64.644	36.286
214	ASN	7.494	116.702	174.522	54.133	39.494
215	GLY	7.788	107.631	173.428	45.137	-
216	ASP	8.464	125.987	173.683	52.885	40.125
217	ARG	7.892	115.294	177.896	53.769	30.411
218	TRP	7.349	120.131	175.584	57.764	29.245
219	PHE	5.885	114.403	175.275	54.414	35.604
220	LEU	6.455	124.562	176.165	54.262	38.981
221	ASN	8.298	122.266	174.662	51.341	39.293
222	ARG	8.040	117.394	176.154	54.279	27.458
223	PHE	7.823	121.273	174.109	55.374	38.242
224	THR	8.069	112.925	174.082	60.786	69.516
225	THR	7.805	117.467	172.288	58.666	68.889
226	THR	8.466	111.547	175.540	58.995	70.808
227	LEU	9.223	124.265	178.655	57.927	39.705
228	ASN	8.579	115.445	177.943	55.781	37.274
229	ASP	7.835	119.076	179.052	56.624	39.216
230	PHE	8.491	120.439	176.676	61.711	37.418
231	ASN	8.634	118.860	177.864	54.910	36.309
232	LEU	7.495	120.134	179.723	57.250	40.031
233	VAL	6.930	119.225	177.495	64.675	29.920
234	ALA	8.337	121.947	180.334	54.822	15.215
235	MET	7.912	114.559	179.496	57.998	30.977
236	LYS	7.348	120.165	177.167	57.348	29.970
237	TYR	7.424	116.568	174.922	57.407	37.251
238	ASN	7.889	115.596	173.457	54.189	35.566
239	TYR	7.998	120.329	176.175	56.641	35.967

**240**	GLU	8.245	121.529	175.050	53.586	29.247
**241**	PRO	-	-	175.997	62.285	29.830
**242**	LEU	9.548	125.434	178.212	52.546	43.256
**243**	THR	10.163	119.859	175.709	58.814	70.706
**244**	GLN	8.940	119.937	177.343	57.152	26.219
**245**	ASP	7.755	117.569	178.967	56.559	38.781
**246**	HIS	7.305	119.821	177.374	57.812	31.220
**247**	VAL	7.589	118.562	179.492	66.094	29.293
**248**	ASP	8.317	120.102	181.027	56.949	38.647
**249**	ILE	8.036	120.702	177.429	63.547	36.228
**250**	LEU	7.328	120.165	176.359	54.513	41.329
**251**	GLY	7.774	108.411	174.222	47.640	-
**252**	PRO	-	-	179.868	65.152	29.935
**253**	LEU	7.372	118.348	179.438	56.421	38.628
**254**	SER	7.745	117.551	177.552	59.924	61.689
**255**	ALA	8.516	123.835	180.228	53.597	16.422
**256**	GLN	7.692	116.653	177.714	57.681	27.289
**257**	THR	7.366	103.682	176.055	61.569	71.421
**258**	GLY	8.045	111.967	173.642	45.072	-
**259**	ILE	7.492	120.471	176.931	58.135	36.816
**260**	ALA	9.432	136.342	178.153	51.721	16.733
**261**	VAL	8.896	124.893	179.517	65.598	29.836
**262**	LEU	8.885	117.192	179.355	57.143	37.628
**263**	ASP	6.951	121.379	179.234	56.192	37.846
**264**	MET	8.041	124.468	179.929	56.572	29.581
**265**	CYS	8.692	117.900	176.342	63.330	24.709
**266**	ALA	7.708	123.912	180.630	54.558	16.413

**267**	SER	7.765	117.272	175.977	62.145	60.925
**268**	LEU	8.297	124.132	176.909	56.792	38.494
**269**	LYS	8.780	119.469	177.288	59.369	30.814
**270**	GLU	7.126	116.872	178.889	58.188	27.620
**271**	LEU	7.623	120.675	180.555	56.825	40.112
**272**	LEU	8.203	119.779	177.856	56.204	38.613
**273**	GLN	7.405	113.562	177.745	57.282	28.542
**274**	ASN	8.326	114.332	176.781	52.878	39.561
**275**	GLY	7.636	109.313	172.924	43.826	-
**276**	MET	8.532	115.193	177.855	55.947	32.083
**277**	ASN	8.988	118.320	175.169	53.264	36.995
**278**	GLY	8.498	105.173	174.426	44.755	-
**279**	ARG	7.238	120.544	174.794	54.320	29.707
**280**	THR	7.705	106.515	175.294	58.892	71.728
**281**	ILE	8.916	121.880	174.800	60.943	39.022
**282**	LEU	9.688	126.809	178.256	54.321	38.755
**283**	GLY	8.939	103.859	173.598	44.835	-
**284**	SER	8.556	115.198	174.000	55.936	-
**285**	ALA	8.158	127.691	174.676	50.521	18.456
**286**	LEU	7.820	117.940	175.692	52.349	42.549
**287**	LEU	8.140	120.125	175.470	54.295	37.263
**288**	GLU	4.215	-	175.576	54.567	28.981
**289**	ASP	7.643	122.852	176.036	51.544	42.687
**290**	GLU	7.581	114.472	173.079	55.160	26.335
**291**	PHE	8.734	115.715	174.547	56.325	39.194
**292**	THR	8.701	110.381	175.274	59.091	68.059
**293**	PRO	-	-	177.919	65.431	29.933

**294**	PHE	7.645	115.855	177.701	59.237	36.949
**295**	ASP	8.340	122.155	179.581	56.885	39.294
**296**	VAL	7.965	120.165	177.730	66.422	29.852
**297**	VAL	7.845	120.368	179.163	65.570	30.044
**298**	ARG	8.397	119.920	178.024	58.654	28.351
**299**	GLN	7.535	116.175	178.003	57.025	27.698
**300**	CYS	8.525	114.907	174.774	60.509	32.038
**301**	SER	7.941	113.536	174.637	58.010	63.144
**302**	GLY	7.558	111.355	173.536	45.217	-
**303**	VAL	7.446	119.197	176.077	62.114	30.840
**304**	THR	7.954	117.606	173.510	59.674	69.946
**305**	PHE	8.059	120.998	174.654	56.566	38.881
**306**	GLN	7.782	126.288	180.526	56.319	29.142

Table S2: M^{pro}_{C145A} assignment (308K)

Res. Number	Res. Type	H	N	C'	Cα	Cβ
1	SER	-	-	171.620	57.273	62.340
2	GLY	8.091	109.726	172.781	43.187	-
3	PHE	7.935	119.313	174.642	58.470	41.133
4	ARG	8.800	127.644	-	51.392	34.165
5	LYS	-	-	175.013	55.635	27.918
6	MET	7.608	128.095	174.188	54.808	34.253
7	ALA	8.591	128.633	177.665	48.796	19.923
8	PHE	8.808	120.353	-	57.913	37.213
9	PRO	-	-	178.583	61.993	28.182
10	SER	7.847	114.960	177.432	58.925	64.926
11	GLY	10.744	121.350	176.205	48.172	-
12	LYS	9.056	119.232	178.260	58.217	31.941
13	VAL	7.305	117.391	178.278	64.064	30.301
14	GLU	8.245	121.092	178.975	59.708	29.301
15	GLY	7.251	101.536	173.971	45.347	-
16	CYS	7.898	116.035	173.678	57.178	28.447
17	MET	7.042	118.131	177.116	53.044	27.648
18	VAL	8.787	114.676	174.095	58.095	33.674
19	GLN	8.662	120.879	174.548	53.819	30.239
20	VAL	8.731	125.547	173.751	60.671	32.493
21	THR	9.107	123.702	173.870	60.136	70.308
22	CYS	8.808	127.518	-	57.280	26.660
23	GLY	9.110	120.030	-	-	-
24	THR	8.607	117.876	174.516	60.695	68.027
25	THR	8.048	122.692	172.259	61.977	68.673
26	THR	8.464	123.959	173.558	60.576	70.012
27	LEU	9.169	125.270	174.140	53.240	39.304
28	ASN	7.743	115.772	172.950	51.879	40.856
29	GLY	8.452	102.853	170.195	42.607	-
30	LEU	8.736	122.927	174.252	52.224	44.026
31	TRP	10.233	132.015	174.459	52.911	29.431
32	LEU	8.981	127.369	177.001	51.736	43.137
33	ASP	8.879	124.716	174.828	56.156	38.119
34	ASP	8.477	123.246	173.281	52.165	37.334
35	VAL	8.070	119.268	173.866	59.983	33.781
36	VAL	8.975	124.892	174.620	59.255	31.541
37	TYR	9.373	127.337	176.364	55.342	38.617
38	CYS	8.917	118.136	-	54.783	28.366
39	PRO	-	-	-	-	-
40	ARG	-	-	-	-	-
41	HIS	-	-	-	-	-
42	VAL	7.129	123.888	-	62.948	29.988
43	ILE	7.364	109.821	175.153	-	-
44	CYS	7.812	120.308	176.393	56.880	28.806
45	THR	9.228	115.641	-	60.419	70.350
46	SER	8.760	116.211	177.010	61.215	70.242
47	GLU	8.055	120.209	178.596	57.903	27.756
48	ASP	7.565	119.919	176.879	55.462	40.465
49	MET	7.160	113.073	177.157	55.241	29.801
50	LEU	7.277	119.902	177.773	56.895	39.856
51	ASN	7.508	112.366	-	51.388	36.716
52	PRO	-	-	176.517	62.897	29.701
53	ASN	8.310	122.504	175.477	50.183	35.583
54	TYR	8.026	120.358	177.215	63.713	37.432
55	GLU	8.617	117.474	178.771	59.744	27.227
56	ASP	7.196	119.289	178.189	55.911	40.674
57	LEU	8.171	117.945	180.299	56.653	40.588
58	LEU	8.234	119.273	179.638	56.740	39.653
59	ILE	7.386	117.185	176.996	62.561	36.222
60	ARG	7.011	117.288	176.578	55.452	28.547
61	LYS	7.701	119.247	175.075	52.876	31.471
62	SER	8.295	115.289	-	55.688	64.273
63	ASN	-	-	-	-	-
64	HIS	7.520	111.672	176.214	56.507	28.453
65	ASN	7.610	116.746	173.292	54.130	38.372
66	PHE	7.342	117.618	173.320	56.531	39.391
67	LEU	8.897	125.999	175.780	52.798	40.789
68	VAL	8.949	128.363	174.454	59.538	30.904
69	GLN	8.681	126.106	173.538	53.492	30.960
70	ALA	8.856	131.033	177.480	48.928	18.727
71	GLY	8.925	115.862	-	46.578	-
72	ASN	8.741	124.736	174.694	52.475	37.097
73	VAL	7.811	121.678	174.457	61.139	31.881
74	GLN	8.421	126.618	175.647	54.332	27.317
75	LEU	8.243	126.438	175.757	52.185	40.502
76	ARG	8.266	124.461	175.086	55.472	29.121
77	VAL	8.082	125.981	176.618	61.890	29.848
78	ILE	9.162	121.558	176.016	59.809	37.085

**79**	GLY	7.649	111.775	-	44.787	-
**80**	HIS	8.109	115.672	172.954	54.687	31.231
**81**	SER	8.265	113.011	171.245	56.654	64.110
**82**	MET	8.898	123.564	174.312	54.284	34.470
**83**	GLN	8.954	129.832	173.061	53.384	28.716
**84**	ASN	8.966	123.970	174.893	55.012	36.253
**85**	CYS	8.437	117.648	174.165	58.232	26.659
**86**	VAL	8.345	119.233	-	59.146	33.600
**87**	LEU	9.014	124.946	174.259	52.479	42.353
**88**	LYS	8.430	121.310	175.972	53.784	30.476
**89**	LEU	9.462	127.178	175.078	51.671	39.229
**90**	LYS	8.355	127.457	176.486	54.174	31.026
**91**	VAL	8.715	120.126	176.114	57.765	33.610
**92**	ASP	8.106	117.536	175.448	53.940	39.710
**93**	THR	6.902	114.310	171.978	60.333	71.405
**94**	ALA	8.515	127.893	177.784	49.006	17.357
**95**	ASN	7.962	121.081	-	49.807	33.391
**96**	PRO	-	-	177.022	63.324	30.078
**97**	LYS	7.478	118.039	175.602	53.774	29.080
**98**	THR	6.918	118.845	-	62.559	69.864
**99**	PRO	-	-	176.171	61.049	30.470
**100**	LYS	8.303	121.123	176.750	57.360	29.892
**101**	TYR	8.021	122.861	173.918	55.488	41.467
**102**	LYS	8.780	118.215	174.488	54.030	34.909
**103**	PHE	8.775	122.786	175.690	52.453	35.931
**104**	VAL	8.224	120.440	173.517	59.100	34.215
**105**	ARG	8.408	126.520	177.400	53.729	29.050
**106**	ILE	8.318	124.224	173.959	59.579	38.618
**107**	GLN	7.983	117.897	-	51.880	27.888
**108**	PRO	-	-	176.821	62.824	30.901
**109**	GLY	9.181	113.297	173.578	43.986	-
**110**	GLN	7.654	117.982	174.965	55.518	28.142
**111**	THR	7.404	109.946	175.066	58.030	71.033
**112**	PHE	8.311	114.456	173.048	55.399	37.377
**113**	SER	8.683	114.106	171.845	56.335	63.523
**114**	VAL	8.944	123.163	174.620	59.962	32.391
**115**	LEU	8.284	126.470	175.359	51.542	41.482
**116**	ALA	8.711	131.483	176.654	51.800	17.172
**117**	CYS	8.521	123.430	170.420	57.098	30.377
**118**	TYR	7.965	115.240	176.664	57.143	40.317
**119**	ASN	10.369	123.585	175.099	53.890	36.568
**120**	GLY	8.861	105.012	172.733	44.336	-
**121**	SER	7.890	115.287	-	54.620	63.307
**122**	PRO	-	-	176.156	62.695	31.525
**123**	SER	9.776	118.372	-	58.014	64.570
**124**	GLY	7.672	104.541	170.030	45.037	-
**125**	VAL	8.865	119.946	174.290	57.728	34.609
**126**	TYR	8.018	120.421	171.613	54.769	38.153
**127**	GLN	8.318	122.641	175.292	53.647	28.658
**128**	CYS	8.669	121.313	170.703	56.181	29.424
**129**	ALA	6.972	117.272	176.686	48.869	19.265
**130**	MET	8.248	120.171	175.075	53.500	28.541
**131**	ARG	8.951	128.897	-	52.429	24.971
**132**	PRO	-	-	175.721	64.369	31.341
**133**	ASN	6.939	111.129	175.405	50.907	35.685
**134**	PHE	8.380	111.011	172.609	61.078	33.748
**135**	THR	7.039	104.561	172.897	59.216	71.821
**136**	ILE	8.680	110.581	175.567	58.753	39.564
**137**	LYS	9.167	125.600	176.834	53.521	28.536
**138**	GLY	9.321	113.183	172.493	42.831	-
**139**	SER	6.207	112.077	173.655	55.431	-
**140**	PHE	7.776	126.260	174.299	55.777	42.793
**141**	LEU	8.675	122.703	-	52.055	-
**142**	ASN	8.713	121.265	-	-	37.137
**143**	GLY	8.867	113.748	174.490	-	-
**144**	SER	8.105	112.228	177.650	57.549	-
**145**	ALA	8.692	128.643	177.031	53.635	17.490
**146**	GLY	9.253	113.802	173.000	44.098	-
**147**	SER	7.874	113.418	-	61.501	62.594
**148**	VAL	-	-	174.869	58.660	35.451
**149**	GLY	9.583	106.755	174.579	43.348	-
**150**	PHE	9.766	121.141	171.965	55.360	41.024
**151**	ASN	8.434	116.763	173.345	51.490	42.140
**152**	ILE	4.359	117.061	173.811	59.409	36.348
**153**	ASP	8.305	128.179	175.907	52.509	41.132
**154**	TYR	8.753	123.104	173.471	60.485	34.615
**155**	ASP	8.354	121.544	174.776	52.557	39.781
**156**	CYS	7.914	119.534	173.907	56.375	27.783
**157**	VAL	8.921	130.172	174.356	61.321	30.316
**158**	SER	8.741	124.191	175.288	55.074	61.779
**159**	PHE	9.145	125.493	175.937	58.318	38.982

160	CYS	8.676	117.681	172.938	54.526	32.149
161	TYR	7.485	125.365	172.109	56.727	42.149
162	MET	8.771	127.996	173.845	53.224	34.159
163	HIS	9.175	127.260	-	57.470	31.492
164	HIS	-	-	-	-	-
165	MET	-	-	172.932	53.988	34.291
166	GLU	8.738	122.986	175.840	54.352	30.373
167	LEU	8.939	127.636	-	52.734	38.458
168	PRO	-	-	-	-	-
169	THR	-	-	175.515	61.317	-
170	GLY	8.426	110.179	173.142	44.876	-
171	VAL	6.191	109.743	174.082	58.658	30.767
172	HIS	8.749	120.927	172.780	58.895	33.392
173	ALA	8.872	121.619	177.559	49.216	22.394
174	GLY	9.586	109.123	171.011	45.011	-
175	THR	7.821	105.690	174.914	57.883	72.277
176	ASP	7.277	117.316	178.354	52.579	39.072
177	LEU	9.124	124.874	176.342	52.092	33.711
178	GLU	7.318	114.265	176.380	54.908	27.934
179	GLY	7.442	104.405	173.646	45.136	-
180	ASN	8.338	120.163	176.493	51.429	37.520
181	PHE	9.614	127.098	178.754	60.172	36.876
182	TYR	8.760	123.253	175.393	59.060	36.872
183	GLY	7.944	111.148	-	43.668	-
184	PRO	-	-	175.296	61.969	27.738
185	PHE	6.590	121.027	172.789	55.644	39.527
186	VAL	8.382	111.367	174.949	57.932	33.821

187	ASP	8.817	120.926	174.633	51.052	35.024
188	ARG	7.714	117.682	174.705	53.769	31.435
189	GLN	8.394	122.787	174.155	54.458	26.258
190	THR	7.604	117.442	-	58.920	68.939
191	ALA	8.150	126.919	176.860	52.381	16.052
192	GLN	7.675	122.640	173.900	54.364	30.077
193	ALA	8.374	124.320	176.466	50.615	19.310
194	ALA	8.484	124.387	178.402	50.383	19.825
195	GLY	9.188	108.185	173.884	43.077	-
196	THR	8.302	117.062	174.206	63.305	68.032
197	ASP	8.717	128.472	175.616	52.486	40.958
198	THR	7.509	114.744	173.058	59.460	70.314
199	THR	7.958	116.862	174.453	62.084	67.741
200	ILE	9.753	128.796	176.132	60.248	34.832
201	THR	7.804	129.364	176.006	66.723	65.529
202	VAL	8.791	120.025	174.489	64.299	29.045
203	ASN	6.706	122.213	179.511	53.737	35.990
204	VAL	7.878	125.397	-	65.498	29.400
205	LEU	8.180	117.158	178.157	57.535	40.400
206	ALA	8.023	120.763	178.226	55.158	16.628
207	TRP	8.397	121.531	-	60.213	27.634
208	LEU	8.511	120.027	180.719	-	-
209	TYR	8.151	117.884	178.425	63.645	36.982
210	ALA	8.062	122.857	180.043	53.820	15.119
211	ALA	8.080	123.706	179.345	54.403	16.028
212	VAL	7.851	120.290	181.211	65.587	29.741
213	ILE	8.497	124.592	177.394	64.663	36.286

214	ASN	7.489	116.778	174.560	54.154	39.494
215	GLY	7.766	107.593	173.470	45.152	-
216	ASP	8.453	126.117	173.771	52.935	40.125
217	ARG	7.842	115.401	177.947	53.807	30.411
218	TRP	7.333	120.033	175.479	57.751	29.245
219	PHE	5.078	114.086	175.246	54.520	35.604
220	LEU	6.433	124.558	176.171	54.302	38.981
221	ASN	8.253	122.442	-	51.404	39.293
222	ARG	8.016	117.522	176.070	54.358	27.458
223	PHE	7.768	121.177	174.119	55.380	38.242
224	THR	7.996	113.088	174.080	60.876	69.516
225	THR	7.763	117.595	172.314	58.725	68.889
226	THR	8.427	111.379	175.557	59.007	70.808
227	LEU	9.159	124.099	178.646	57.957	39.705
228	ASN	8.503	115.345	177.856	55.837	37.274
229	ASP	7.790	119.106	179.002	56.661	39.216
230	PHE	8.464	120.355	176.697	61.731	37.418
231	ASN	8.636	118.937	177.852	54.902	36.309
232	LEU	7.482	120.181	179.677	57.278	40.031
233	VAL	6.933	119.191	-	64.692	29.920
234	ALA	8.328	122.064	180.344	54.846	15.215
235	MET	7.893	114.543	179.468	58.042	30.977
236	LYS	7.351	120.155	177.155	57.350	29.970
237	TYR	7.415	116.654	174.939	57.417	37.251
238	ASN	7.879	115.610	173.455	54.206	35.566
239	TYR	7.988	120.381	176.166	56.642	35.967
240	GLU	8.202	121.576	-	53.631	29.247

241	PRO	-	-	176.042	62.251	29.830
242	LEU	9.505	125.057	178.245	52.625	43.256
243	THR	10.081	119.414	175.774	58.832	70.706
244	GLN	8.884	119.913	177.379	57.288	26.219
245	ASP	7.746	117.547	178.936	56.546	38.781
246	HIS	7.281	119.712	177.361	57.881	31.220
247	VAL	7.543	118.515	179.427	66.085	29.293
248	ASP	8.243	120.080	180.998	56.991	38.647
249	ILE	7.986	120.501	-	63.522	36.228
250	LEU	7.300	119.953	176.530	54.523	41.329
251	GLY	7.768	108.592	-	47.656	-
252	PRO	-	-	179.786	-	29.935
253	LEU	7.298	118.045	179.451	56.379	38.628
254	SER	7.732	117.542	177.537	59.957	61.689
255	ALA	8.488	123.921	180.194	53.642	16.422
256	GLN	7.642	116.565	177.693	57.660	27.289
257	THR	7.357	103.796	176.041	61.631	71.421
258	GLY	8.035	111.950	173.691	45.084	-
259	ILE	7.486	120.569	176.907	58.219	36.816
260	ALA	9.361	136.243	178.203	51.728	16.733
261	VAL	8.834	124.804	179.479	65.628	29.836
262	LEU	8.818	117.330	179.332	57.149	37.628
263	ASP	6.951	121.448	179.276	56.227	37.846
264	MET	8.011	124.420	179.908	56.580	29.581
265	CYS	8.681	118.007	176.348	63.348	24.709
266	ALA	7.706	123.941	180.507	54.587	16.413
267	SER	7.740	117.073	175.974	62.139	60.925
268	LEU	8.281	124.121	176.951	56.824	38.494
269	LYS	8.751	119.555	177.354	59.406	30.814
270	GLU	7.130	117.008	178.940	58.221	27.620
271	LEU	7.614	120.788	180.504	56.860	40.112
272	LEU	8.186	119.742	177.845	56.255	38.613
273	GLN	7.387	113.634	177.700	57.313	28.542
274	ASN	8.312	114.433	176.758	52.968	39.561
275	GLY	7.635	109.317	172.975	43.832	-
276	MET	8.460	115.287	177.847	55.960	32.083
277	ASN	8.915	118.088	175.173	53.279	36.995
278	GLY	8.444	105.266	174.424	44.793	-
279	ARG	7.237	120.572	174.794	54.338	29.707
280	THR	7.654	106.562	175.283	58.919	71.728
281	ILE	8.885	121.887	-	61.012	39.022
282	LEU	9.685	126.955	178.140	-	38.755
283	GLY	8.876	103.771	-	44.833	-
284	SER	8.484	115.242	174.010	-	-
285	ALA	8.120	127.717	174.694	50.582	18.456
286	LEU	7.797	117.962	-	52.397	42.549
287	LEU	8.110	120.130	175.457	-	37.263
288	GLU	4.220	125.150	175.585	54.555	28.981
289	ASP	7.638	122.801	176.063	51.574	42.687
290	GLU	7.558	114.643	173.137	55.166	26.335
291	PHE	8.697	115.843	174.595	56.352	39.194
292	THR	8.697	110.527	-	59.115	68.059
293	PRO	-	-	177.846	65.427	29.933
294	PHE	7.592	115.744	-	59.269	36.949
295	ASP	8.300	122.117	179.559	56.957	39.294
296	VAL	7.944	120.129	177.768	66.460	29.852
297	VAL	7.833	120.314	179.115	65.676	30.044
298	ARG	8.366	119.889	178.008	58.678	28.351
299	GLN	7.527	116.186	177.903	57.060	27.698
300	CYS	8.486	114.777	174.801	60.409	32.038
301	SER	7.923	113.628	174.631	58.069	63.144
302	GLY	7.552	111.205	173.548	45.210	-
303	VAL	7.409	119.134	176.016	62.050	30.840
304	THR	7.890	117.453	173.531	59.740	69.946
305	PHE	8.025	121.119	174.630	56.611	38.881
306	GLN	7.745	126.342	-	56.369	29.142

Table S3: M^{pro}_{H41Q} assignment (308K)

Res. Number	Res. Type	H	N	C'	Cα	Cβ
1	SER	-	-	171.617	57.706	62.228
2	GLY	8.113	109.703	172.908	43.700	-
3	PHE	7.937	119.309	174.699	58.877	41.823
4	ARG	8.786	127.605	-	-	35.362
5	LYS	-	-	174.937	56.095	28.930
6	MET	7.660	128.053	174.140	55.210	35.250
7	ALA	8.572	128.522	177.573	49.253	20.683
8	PHE	8.783	120.219	-	58.375	38.047
9	PRO	-	-	178.859	62.501	-
10	SER	7.838	115.053	177.383	59.342	65.529
11	GLY	10.775	121.405	176.191	48.585	-
12	LYS	9.056	119.174	178.212	58.644	32.911
13	VAL	7.321	117.296	178.361	64.573	31.211
14	GLU	8.282	121.090	178.882	60.236	29.775
15	GLY	7.252	101.379	173.966	45.824	-
16	CYS	7.910	115.985	173.832	57.603	29.011
17	MET	7.065	118.194	177.177	53.501	28.451
18	VAL	8.774	114.668	174.052	58.618	34.594
19	GLN	8.648	120.932	174.616	54.298	31.134
20	VAL	8.774	125.722	173.874	61.095	33.106
21	THR	9.118	123.599	173.774	60.566	71.147
22	CYS	8.852	127.480	-	57.741	27.164
23	GLY	9.145	120.303	174.469	-	-
24	THR	8.593	117.854	174.494	61.111	68.346
25	THR	8.067	122.846	172.159	62.321	69.129
26	THR	8.503	123.856	173.195	60.741	70.782
27	LEU	9.196	123.470	174.156	54.109	39.619
28	ASN	7.755	115.747	172.840	52.706	41.387
29	GLY	8.483	103.055	170.290	43.093	-
30	LEU	8.771	122.752	174.154	52.722	45.105
31	TRP	10.247	131.921	174.526	53.405	30.493
32	LEU	9.027	127.474	176.919	52.221	44.039
33	ASP	8.916	124.803	174.921	56.576	38.929
34	ASP	8.507	123.222	173.202	52.596	38.095
35	VAL	8.084	119.252	173.943	60.516	34.718
36	VAL	9.036	124.981	174.531	59.783	32.480
37	TYR	9.464	127.525	176.431	55.761	39.331
38	CYS	9.006	117.707	-	54.943	29.043
39	PRO	-	-	177.624	63.095	31.730
40	ARG	8.284	122.612	175.755	58.394	28.377
41	GLN	8.060	115.739	175.853	57.741	29.049
42	VAL	7.417	124.183	173.993	63.513	30.958
43	ILE	7.449	110.074	174.696	61.703	-
44	CYS	7.746	120.307	176.460	56.794	29.262
45	THR	9.242	115.404	-	60.791	71.049
46	SER	8.764	116.177	177.101	-	-
47	GLU	8.029	120.292	178.832	58.437	28.569
48	ASP	7.568	120.178	177.131	56.137	41.080
49	MET	7.175	112.902	177.347	55.378	30.388
50	LEU	7.262	119.930	177.984	57.295	40.803
51	ASN	7.479	111.985	-	51.928	37.341
52	PRO	-	-	176.504	63.337	30.407
53	ASN	8.298	122.625	175.430	50.565	36.201
54	TYR	8.013	120.290	177.199	64.178	38.212
55	GLU	8.632	117.289	178.742	60.208	28.174
56	ASP	7.208	119.231	178.216	56.303	41.430
57	LEU	8.185	117.971	180.252	57.145	41.650
58	LEU	8.225	119.349	179.646	57.141	40.835
59	ILE	7.378	116.954	176.903	63.041	37.296
60	ARG	7.010	117.226	176.621	55.885	29.630
61	LYS	7.735	119.117	174.976	53.285	32.451
62	SER	8.289	114.954	-	56.114	65.017
63	ASN	-	-	-	-	-
64	HIS	-	-	176.134	56.933	-
65	ASN	7.627	116.663	173.377	54.558	39.093
66	PHE	7.362	117.524	173.331	57.024	40.412
67	LEU	8.954	125.974	175.825	53.253	42.046
68	VAL	8.917	128.070	174.378	60.127	31.880
69	GLN	8.766	126.291	173.603	53.988	31.782
70	ALA	8.884	131.192	177.437	49.407	18.989
71	GLY	8.917	115.589	-	47.053	-
72	ASN	8.771	124.713	174.687	52.986	38.075
73	VAL	7.815	121.529	174.445	61.623	32.739
74	GLN	8.414	126.492	175.657	54.674	28.340
75	LEU	8.247	126.203	175.796	52.652	41.741
76	ARG	8.283	124.375	175.097	55.903	30.147
77	VAL	8.121	125.866	176.645	62.378	30.737
78	ILE	9.181	121.520	175.948	60.295	38.026
79	GLY	7.628	111.592	-	45.306	-

80	HIS	8.121	115.745	172.856	55.240	32.211
81	SER	8.237	112.786	171.142	57.125	64.756
82	MET	8.932	123.969	174.237	54.765	35.356
83	GLN	8.995	129.995	172.928	53.855	29.510
84	ASN	8.981	124.007	175.016	55.330	36.839
85	CYS	8.367	117.331	174.270	58.717	27.279
86	VAL	8.395	119.753	173.614	59.723	34.610
87	LEU	9.173	124.862	174.336	52.894	43.474
88	LYS	8.462	121.230	175.974	54.293	31.619
89	LEU	9.478	127.120	175.061	52.096	40.351
90	LYS	8.378	127.515	176.548	54.724	31.937
91	VAL	8.736	120.096	176.027	58.260	34.437
92	ASP	8.128	117.473	175.470	54.340	40.424
93	THR	6.924	114.245	171.971	60.805	72.075
94	ALA	8.536	127.856	177.733	49.473	17.975
95	ASN	7.972	121.045	-	50.180	34.060
96	PRO	-	-	176.959	63.789	31.151
97	LYS	7.492	117.985	175.634	54.232	30.036
98	THR	6.944	118.836	-	63.012	70.485
99	PRO	-	-	176.267	61.508	31.204
100	LYS	8.317	121.067	176.758	57.809	30.751
101	TYR	8.043	122.771	173.859	55.949	42.244
102	LYS	8.818	118.176	174.519	54.495	35.864
103	PHE	8.795	122.846	175.571	52.896	36.752
104	VAL	8.255	120.540	173.454	59.619	35.200
105	ARG	8.415	126.433	177.387	54.201	30.084
106	ILE	8.315	124.091	173.837	60.102	39.454
107	GLN	7.977	117.726	-	52.361	28.872
108	PRO	-	-	176.881	63.316	-
109	GLY	9.206	113.271	173.485	44.460	30.959
110	GLN	7.677	117.908	175.047	55.964	28.992
111	THR	7.416	109.911	175.002	58.431	71.762
112	PHE	8.357	114.441	172.960	55.833	38.245
113	SER	8.704	114.107	171.933	56.807	64.259
114	VAL	8.949	123.285	174.491	60.566	33.252
115	LEU	8.370	126.428	175.395	51.924	42.667
116	ALA	8.747	131.428	176.688	52.291	18.057
117	CYS	8.399	123.421	170.667	57.465	30.821
118	TYR	8.033	116.377	176.485	57.393	40.657
119	ASN	10.111	124.272	174.935	54.222	36.947
120	GLY	8.783	104.644	172.815	44.749	-
121	SER	7.865	115.343	-	55.022	63.786
122	PRO	-	-	176.227	63.201	32.282
123	SER	9.721	118.588	175.445	58.583	65.064
124	GLY	7.694	105.191	170.103	-	-
125	VAL	8.899	120.035	174.104	58.265	35.462
126	TYR	7.996	120.526	171.639	55.205	39.032
127	GLN	8.342	122.581	175.326	54.131	29.624
128	CYS	8.682	121.251	170.732	56.671	30.384
129	ALA	6.996	117.280	176.791	49.342	20.037
130	MET	8.286	120.222	174.959	54.007	29.740
131	ARG	9.026	128.986	-	52.897	25.961
132	PRO	-	-	175.842	64.755	31.941
133	ASN	6.966	111.161	175.333	51.321	36.333
134	PHE	8.394	110.949	172.653	61.471	34.569
135	THR	7.063	104.715	173.051	59.647	72.660
136	ILE	8.771	110.263	175.555	59.245	39.868
137	LYS	9.179	125.392	176.794	54.174	-
138	GLY	9.283	113.032	172.452	43.268	-
139	SER	6.227	111.878	173.557	55.805	-
140	PHE	7.820	126.247	174.349	56.312	43.425
141	LEU	8.609	122.633	-	52.531	-
142	ASN	8.708	121.541	175.657	-	-
143	GLY	8.758	112.151	174.875	-	-
144	SER	8.108	112.362	177.373	58.109	-
145	CYS	8.196	125.873	175.008	61.646	25.804
146	GLY	9.432	118.649	172.440	44.950	-
147	SER	7.891	113.883	173.697	62.101	63.549
148	VAL	7.691	118.190	174.776	59.305	35.509
149	GLY	9.552	106.838	174.546	43.852	-
150	PHE	9.788	120.947	171.982	55.880	41.765
151	ASN	8.484	116.775	-	51.940	42.779
152	ILE	-	-	173.935	59.748	37.259
153	ASP	8.345	128.412	175.803	52.853	41.864
154	TYR	8.760	123.143	173.570	60.822	35.323
155	ASP	8.372	121.286	174.697	53.077	40.398
156	CYS	7.926	119.474	173.888	56.848	28.403
157	VAL	8.949	130.097	174.319	61.825	31.179
158	SER	8.772	124.279	175.334	55.494	62.569
159	PHE	9.153	125.383	175.904	58.866	39.870
160	CYS	8.688	117.487	172.906	55.038	32.875

161	TYR	7.511	125.284	171.935	57.303	43.062
162	MET	8.665	128.345	173.884	53.750	35.540
163	HIS	9.167	127.275	176.398	57.991	32.419
164	HIS	7.632	122.620	174.348	58.105	31.042
165	MET	8.388	114.710	173.587	54.074	34.869
166	GLU	8.748	123.547	175.687	55.058	31.251
167	LEU	8.966	127.838	-	53.213	39.514
168	PRO	-	-	-	-	-
169	THR	-	-	175.449	61.633	-
170	GLY	8.433	110.082	173.178	45.410	-
171	VAL	6.200	109.663	173.965	59.104	31.751
172	HIS	8.787	120.674	172.854	59.165	34.287
173	ALA	8.935	121.447	177.487	49.670	23.386
174	GLY	9.686	109.140	170.895	45.491	-
175	THR	7.896	105.780	174.820	58.311	72.892
176	ASP	7.266	117.236	178.297	52.864	39.918
177	LEU	9.140	124.823	176.397	52.650	35.066
178	GLU	7.328	114.204	176.451	55.391	28.871
179	GLY	7.449	104.294	173.527	45.568	-
180	ASN	8.331	120.036	176.439	51.817	38.460
181	PHE	9.609	127.087	178.771	60.489	37.632
182	TYR	8.820	123.124	175.371	59.371	37.123
183	GLY	7.962	111.065	-	44.154	-
184	PRO	-	-	175.238	62.372	28.443
185	PHE	6.643	121.069	172.556	56.012	40.554
186	VAL	8.363	110.968	174.944	58.476	34.716
187	ASP	8.885	121.082	174.463	51.459	35.747
188	ARG	7.650	117.260	174.562	54.184	32.463
189	GLN	8.488	123.067	174.041	54.870	26.899
190	THR	7.658	117.722	-	59.289	69.695
191	ALA	-	-	176.918	52.878	16.967
192	GLN	7.675	122.619	173.857	54.857	31.067
193	ALA	8.395	124.113	176.508	51.040	20.207
194	ALA	8.516	124.415	178.342	50.863	20.216
195	GLY	9.169	108.039	173.890	43.534	-
196	THR	8.314	117.038	174.257	63.743	68.818
197	ASP	8.726	128.434	175.607	52.861	41.649
198	THR	7.526	114.813	173.077	59.916	70.948
199	THR	7.978	116.890	174.406	62.533	68.436
200	ILE	9.770	128.764	176.180	60.726	35.804
201	THR	7.821	129.324	175.997	67.177	66.363
202	VAL	8.809	119.983	174.578	64.787	29.830
203	ASN	6.724	122.199	179.438	54.136	36.747
204	VAL	7.888	125.313	178.802	66.000	30.271
205	LEU	8.195	117.147	178.060	57.877	-
206	ALA	8.049	120.720	178.307	55.511	-
207	TRP	8.411	121.575	-	-	-
208	LEU	8.525	120.045	180.733	-	-
209	TYR	8.163	117.929	-	64.088	37.857
210	ALA	-	-	180.009	-	-
211	ALA	8.099	123.666	179.434	54.866	17.025
212	VAL	7.874	120.270	181.115	66.035	30.591
213	ILE	8.502	124.497	177.407	65.095	37.263
214	ASN	7.523	116.772	174.483	54.563	39.911
215	GLY	7.757	107.435	173.464	45.597	-
216	ASP	8.473	126.078	173.728	53.356	40.890
217	ARG	7.858	115.147	178.001	54.271	31.260
218	TRP	7.345	120.037	-	-	29.983
219	PHE	-	-	175.312	54.888	36.704
220	LEU	6.448	124.504	176.114	54.726	40.153
221	ASN	8.267	122.416	174.592	51.806	39.989
222	ARG	8.026	117.508	176.148	54.797	28.298
223	PHE	7.783	121.114	174.104	55.811	38.914
224	THR	8.008	113.059	174.096	61.322	70.213
225	THR	7.781	117.550	172.330	59.175	69.471
226	THR	8.442	111.331	175.524	59.442	71.630
227	LEU	9.167	124.067	178.665	58.372	40.786
228	ASN	8.497	115.291	177.860	56.220	37.939
229	ASP	7.803	119.061	178.975	57.058	40.072
230	PHE	8.478	120.300	176.767	62.141	38.182
231	ASN	8.653	118.899	177.782	55.297	37.205
232	LEU	7.497	120.139	179.764	57.742	41.151
233	VAL	6.949	119.153	177.425	65.179	30.818
234	ALA	8.342	122.046	180.450	55.298	16.153
235	MET	7.908	114.498	179.432	58.484	31.970
236	LYS	7.367	120.147	177.163	57.815	31.030
237	TYR	7.432	116.619	174.911	57.847	37.984
238	ASN	7.896	115.576	173.447	54.616	36.242
239	TYR	8.002	120.288	176.097	57.091	36.854
240	GLU	8.214	121.547	-	54.132	30.166
241	PRO	-	-	176.029	62.699	30.971

**242**	LEU	9.517	124.985	178.178	53.077	44.273
**243**	THR	10.092	119.319	175.842	59.266	71.461
**244**	GLN	8.899	119.893	177.332	57.728	27.003
**245**	ASP	7.768	117.513	178.946	56.936	39.482
**246**	HIS	7.297	119.657	177.390	58.350	32.001
**247**	VAL	7.553	118.456	179.386	66.555	30.188
**248**	ASP	8.252	120.024	181.045	57.375	39.542
**249**	ILE	7.993	120.590	177.238	63.991	37.288
**250**	LEU	7.307	119.861	176.636	54.940	41.841
**251**	GLY	7.769	108.506	-	48.125	-
**252**	PRO	-	-	179.805	65.577	-
**253**	LEU	7.286	117.951	179.439	56.851	39.490
**254**	SER	7.741	117.555	177.521	60.437	62.535
**255**	ALA	8.488	123.836	180.261	54.106	17.292
**256**	GLN	7.652	116.573	177.654	58.149	28.219
**257**	THR	7.367	103.664	176.023	62.016	71.772
**258**	GLY	8.039	111.854	173.676	45.556	-
**259**	ILE	7.508	120.558	176.922	58.739	37.759
**260**	ALA	9.369	136.998	178.260	52.214	17.708
**261**	VAL	8.847	124.747	179.416	66.120	30.749
**262**	LEU	8.824	117.300	179.361	57.586	38.782
**263**	ASP	6.969	121.412	179.227	56.623	38.548
**264**	MET	8.022	124.360	179.986	57.004	30.488
**265**	CYS	8.698	117.979	176.293	63.799	25.276
**266**	ALA	7.721	123.888	180.504	55.041	17.284
**267**	SER	7.753	117.004	175.996	62.572	61.515
**268**	LEU	8.298	124.080	176.923	57.265	39.597
**269**	LYS	8.763	119.512	177.372	59.871	31.796
**270**	GLU	7.147	116.980	178.880	58.692	28.513
**271**	LEU	7.630	120.751	180.598	57.310	41.173
**272**	LEU	8.201	119.705	177.770	56.716	39.613
**273**	GLN	7.402	113.594	177.768	57.760	29.282
**274**	ASN	8.312	114.445	176.692	53.385	39.988
**275**	GLY	7.632	109.156	173.033	44.292	-
**276**	MET	8.473	115.279	177.827	56.214	32.697
**277**	ASN	8.927	118.153	175.194	53.782	-
**278**	GLY	8.444	105.203	174.417	45.252	-
**279**	ARG	7.253	120.520	174.706	54.788	30.586
**280**	THR	7.666	106.507	175.282	59.366	72.366
**281**	ILE	8.903	121.842	-	-	40.263
**282**	LEU	9.694	126.962	178.182	-	39.087
**283**	GLY	8.881	103.709	-	45.316	-
**284**	SER	8.473	115.464	173.940	56.398	-
**285**	ALA	8.145	127.735	174.660	51.041	-
**286**	LEU	7.815	117.954	-	52.870	43.695
**287**	LEU	8.078	120.098	-	-	-
**288**	GLU	-	-	175.607	55.012	29.732
**289**	ASP	7.649	122.622	176.028	51.983	43.419
**290**	GLU	7.565	114.660	173.139	55.659	27.242
**291**	PHE	8.695	115.855	174.556	56.758	39.952
**292**	THR	8.710	110.534	-	59.566	68.796
**293**	PRO	-	-	177.721	65.858	30.727
**294**	PHE	7.600	115.709	177.699	59.714	37.701
**295**	ASP	8.315	122.122	179.526	57.380	39.965
**296**	VAL	7.974	120.003	177.851	66.981	30.667
**297**	VAL	7.941	120.569	179.033	66.169	30.925
**298**	ARG	8.314	119.698	177.894	59.099	29.371
**299**	GLN	7.518	115.712	177.639	57.398	28.984
**300**	CYS	8.442	114.433	174.846	60.516	-
**301**	SER	7.965	114.315	174.558	58.678	63.495
**302**	GLY	7.668	110.930	173.563	45.478	-
**303**	VAL	7.448	119.098	175.916	62.297	31.764
**304**	THR	7.924	117.471	173.605	60.434	70.267
**305**	PHE	8.074	121.676	174.575	57.049	39.482
**306**	GLN	7.752	126.383	-	56.900	29.914

Table S4: M^{pro}_{C145A}:SAVLQSGFRK assignment (298K)

Res. Number	Res. Type	H	N	C'	Cα	Cβ
1	SER	-	-	171.328	57.281	-
2	GLY	8.035	108.859	172.962	42.994	-
3	PHE	8.021	119.376	174.634	58.484	41.245
4	ARG	8.852	127.739	177.414	51.206	34.147
5	LYS	8.768	122.278	174.978	55.658	27.733
6	MET	7.615	127.902	175.502	54.780	34.307
7	ALA	8.625	128.795	177.748	48.792	19.863
8	PHE	8.887	120.303	-	57.940	37.277
9	PRO	-	-	-	62.548	-
10	SER	7.905	117.445	-	59.169	-
11	GLY	10.748	121.256	176.194	48.168	-
12	LYS	9.103	119.135	178.290	58.180	31.939
13	VAL	7.349	117.367	-	63.950	30.386
14	GLU	8.185	120.842	-	59.673	-
15	GLY	7.303	101.585	-	45.317	-
16	CYS	7.915	116.037	173.584	58.131	-
17	MET	7.039	117.886	177.068	52.989	27.687
18	VAL	8.763	114.402	174.120	58.085	33.702
19	GLN	8.787	121.576	174.603	53.928	29.876
20	VAL	8.587	125.512	173.914	60.737	-
21	THR	8.971	123.312	173.318	60.154	-
22	CYS	8.893	128.159	174.998	57.198	26.499
23	GLY	9.211	120.810	174.279	46.048	-
24	THR	8.812	117.723	173.940	60.332	-
25	THR	8.058	122.928	172.359	62.009	68.559
26	THR	8.455	121.815	170.754	58.646	70.405
27	LEU	9.007	121.485	174.807	54.337	37.114
28	ASN	8.206	117.559	-	51.836	40.841
29	GLY	8.549	103.031	170.072	42.453	-
30	LEU	8.748	122.787	-	52.118	43.844
31	TRP	10.255	131.935	174.484	52.963	-
32	LEU	9.007	127.252	177.066	51.691	43.004
33	ASP	8.961	124.956	174.835	56.144	38.233
34	ASP	8.470	123.127	173.238	52.123	37.434
35	VAL	8.075	119.199	173.717	60.109	33.827
36	VAL	8.955	124.931	175.232	59.223	31.632
37	TYR	9.342	127.184	176.530	55.125	-
38	CYS	8.923	117.453	-	54.736	-
39	PRO	-	-	177.334	62.341	-
40	ARG	8.119	120.279	-	57.389	29.620
41	HIS	7.518	114.031	174.863	58.760	-
42	VAL	6.754	122.385	173.792	62.516	27.096
43	ILE	7.262	108.976	174.996	61.292	-
44	CYS	7.834	120.336	176.169	58.369	-
45	THR	9.083	114.093	176.724	60.639	-
46	SER	9.079	116.890	177.294	61.325	-
47	GLU	8.074	119.511	178.115	57.742	27.765
48	ASP	7.587	118.977	175.839	55.001	41.651
49	MET	7.086	112.716	175.807	58.296	31.276
50	LEU	7.255	120.301	176.502	57.222	-
51	ASN	7.560	112.107	-	50.590	37.207
52	PRO	-	-	176.611	62.798	29.768
53	ASN	8.390	122.440	175.519	50.048	35.525
54	TYR	8.026	120.093	177.271	63.183	37.319
55	GLU	8.678	117.479	178.901	59.831	27.244
56	ASP	7.234	119.605	178.250	55.982	40.733
57	LEU	8.293	117.761	180.369	56.594	40.551
58	LEU	8.263	119.045	179.924	56.557	39.749
59	ILE	7.536	118.539	176.910	62.783	36.307
60	ARG	6.958	116.859	176.431	55.466	28.612
61	LYS	7.684	118.905	175.033	52.685	31.558
62	SER	8.425	115.606	-	55.444	64.409
63	ASN	-	-	-	-	-
64	HIS	-	-	176.145	56.741	-
65	ASN	7.562	116.476	173.255	54.339	38.507
66	PHE	7.329	117.342	173.029	56.669	39.201
67	LEU	8.918	126.202	175.870	52.688	40.600
68	VAL	9.164	129.479	174.406	59.621	30.692
69	GLN	8.801	126.338	173.516	53.383	31.059
70	ALA	8.914	131.285	177.455	48.895	18.502
71	GLY	8.956	115.906	174.740	46.517	-
72	ASN	8.857	125.180	174.792	52.567	37.130
73	VAL	7.843	121.766	174.453	61.129	31.890
74	GLN	8.511	126.644	175.725	54.163	27.291
75	LEU	8.280	126.527	175.754	52.133	40.577
76	ARG	8.365	124.785	175.054	55.598	29.030
77	VAL	8.079	126.218	176.606	61.829	29.629
78	ILE	9.220	120.973	175.921	59.762	37.026

79	GLY	7.664	112.031	171.475	44.971	-
80	HIS	8.410	115.315	172.062	54.331	-
81	SER	8.350	113.779	170.931	56.701	-
82	MET	8.954	123.602	174.156	54.174	34.633
83	GLN	8.980	129.882	173.003	53.393	28.720
84	ASN	9.038	124.116	-	54.960	36.241
85	CYS	8.462	117.442	174.350	58.083	-
86	VAL	8.390	119.175	-	59.032	33.836
87	LEU	9.121	123.953	174.400	52.281	-
88	LYS	8.424	120.322	175.928	53.841	-
89	LEU	9.489	127.188	175.019	51.356	39.178
90	LYS	8.189	127.553	176.528	54.177	31.067
91	VAL	8.796	120.332	176.096	57.751	33.674
92	ASP	8.078	117.503	175.382	53.845	39.732
93	THR	6.898	114.077	171.966	60.279	71.464
94	ALA	8.587	127.837	177.802	48.995	17.374
95	ASN	7.996	120.998	-	49.772	33.424
96	PRO	-	-	177.032	63.299	30.166
97	LYS	7.472	117.982	175.595	53.768	29.031
98	THR	6.940	118.747	-	62.548	70.025
99	PRO	-	-	176.193	61.049	31.297
100	LYS	8.370	121.155	176.784	57.333	29.813
101	TYR	8.098	122.879	173.965	55.538	41.501
102	LYS	8.807	118.197	174.544	54.056	34.979
103	PHE	8.847	122.778	175.709	52.365	36.090
104	VAL	8.292	120.359	173.497	59.056	34.303
105	ARG	8.483	126.485	177.490	53.654	28.997
106	ILE	8.354	124.463	173.958	59.592	38.691
107	GLN	8.032	117.836	-	51.850	27.936
108	PRO	-	-	176.900	62.780	30.917
109	GLY	9.266	113.503	173.669	43.939	-
110	GLN	7.680	118.034	174.948	55.423	28.225
111	THR	7.454	109.900	175.090	58.011	70.947
112	PHE	8.367	114.392	-	55.461	37.386
113	SER	8.702	114.089	171.824	56.368	63.589
114	VAL	8.989	123.125	174.651	59.963	32.576
115	LEU	8.339	126.251	175.417	51.486	-
116	ALA	8.771	131.442	176.783	51.781	17.183
117	CYS	8.499	123.158	-	57.205	-
118	TYR	7.996	114.800	176.656	57.235	-
119	ASN	10.280	122.643	175.322	53.579	-
120	GLY	8.706	104.634	172.664	44.326	-
121	SER	7.877	115.091	-	54.676	-
122	PRO	-	-	-	62.696	-
123	SER	9.801	118.536	-	58.102	66.199
124	GLY	7.716	104.777	170.052	45.022	-
125	VAL	8.897	120.007	174.393	57.795	34.610
126	TYR	8.065	120.418	171.668	54.833	38.146
127	GLN	8.398	122.689	175.353	53.653	29.510
128	CYS	8.706	121.526	170.837	56.267	28.855
129	ALA	6.997	117.202	176.690	48.879	19.304
130	MET	8.314	120.209	174.956	53.424	28.468
131	ARG	9.043	129.177	-	52.475	24.887
132	PRO	-	-	175.913	64.412	31.427
133	ASN	7.018	111.378	175.335	50.939	35.631
134	PHE	8.417	110.924	174.710	61.076	33.700
135	THR	7.038	104.354	172.855	59.050	71.949
136	ILE	8.879	110.571	-	58.670	39.771
137	LYS	9.280	125.635	176.660	53.848	29.131
138	GLY	9.270	113.518	-	42.741	-
139	SER	6.152	112.203	173.668	55.436	-
140	PHE	7.876	124.170	174.189	56.041	42.508
141	LEU	8.700	120.601	179.400	51.625	36.410
142	ASN	8.938	122.450	175.283	56.612	36.227
143	GLY	7.743	111.151	-	44.602	-
144	SER	8.332	113.326	177.763	-	-
145	ALA	8.022	127.497	176.826	53.225	-
146	GLY	9.544	114.509	170.882	44.149	-
147	SER	7.844	113.205	-	61.519	61.388
148	VAL	-	-	-	58.570	-
149	GLY	9.569	106.694	174.661	43.299	-
150	PHE	9.791	121.038	171.907	55.372	41.015
151	ASN	8.409	116.636	-	51.512	42.290
152	ILE	-	-	173.836	59.390	36.626
153	ASP	8.309	128.013	175.899	52.502	41.106
154	TYR	8.819	123.381	-	60.536	34.649
155	ASP	-	-	174.712	52.557	-
156	CYS	7.942	119.556	173.994	56.483	27.660
157	VAL	8.965	130.507	174.369	61.306	30.393
158	SER	8.776	124.217	175.323	55.208	61.859
159	PHE	9.185	125.617	175.807	58.314	39.014

**160**	CYS	8.717	117.598	172.922	54.547	-
**161**	TYR	7.492	125.298	171.915	56.779	42.095
**162**	MET	8.790	128.126	173.941	53.311	34.427
**163**	HIS	9.187	126.615	173.116	57.873	31.867
**164**	HIS	7.325	121.831	-	58.272	-
**165**	MET	8.382	115.147	172.009	54.533	31.739
**166**	GLU	9.157	122.824	175.868	53.949	31.938
**167**	LEU	9.110	127.559	-	52.381	38.630
**168**	PRO	-	-	-	-	28.504
**169**	THR	8.548	123.834	175.313	61.185	68.594
**170**	GLY	8.506	110.502	-	45.027	-
**171**	VAL	6.174	108.558	174.063	58.166	31.061
**172**	HIS	9.121	120.412	172.979	59.825	33.637
**173**	ALA	8.890	120.709	-	49.343	-
**174**	GLY	9.540	108.683	171.064	44.824	-
**175**	THR	7.858	105.436	174.811	57.670	72.566
**176**	ASP	7.312	117.255	-	52.611	39.366
**177**	LEU	9.115	124.630	-	52.130	34.094
**178**	GLU	7.342	114.143	-	54.808	28.052
**179**	GLY	7.463	104.355	173.689	45.192	-
**180**	ASN	8.381	120.242	176.522	51.439	37.595
**181**	PHE	9.726	127.002	178.860	60.332	38.768
**182**	TYR	8.708	123.221	175.308	59.089	36.757
**183**	GLY	8.013	111.367	-	43.628	-
**184**	PRO	-	-	175.204	61.878	27.500
**185**	PHE	6.752	121.177	172.847	55.453	39.861
**186**	VAL	8.272	111.180	174.961	57.881	33.668
**187**	ASP	9.143	120.539	173.670	51.595	33.715
**188**	ARG	7.950	118.834	174.854	53.879	31.349
**189**	GLN	8.498	121.263	174.424	55.278	24.493
**190**	THR	7.597	112.303	175.566	58.180	70.155
**191**	ALA	8.525	125.717	176.577	52.700	-
**192**	GLN	7.323	124.507	173.465	54.578	33.185
**193**	ALA	8.475	122.852	176.474	50.153	19.983
**194**	ALA	8.661	124.533	178.440	50.421	19.447
**195**	GLY	9.285	108.328	173.886	43.031	-
**196**	THR	8.382	117.111	174.286	63.385	68.113
**197**	ASP	8.805	128.569	-	52.426	40.788
**198**	THR	7.562	114.447	173.051	59.440	70.434
**199**	THR	7.957	116.942	174.388	62.087	67.779
**200**	ILE	9.798	128.765	176.130	60.191	35.025
**201**	THR	7.815	129.285	175.980	66.726	65.733
**202**	VAL	8.833	119.953	-	64.260	28.979
**203**	ASN	6.725	122.146	179.522	53.694	36.086
**204**	VAL	7.893	125.348	-	65.493	29.525
**205**	LEU	8.179	116.991	-	57.665	40.427
**206**	ALA	8.058	120.633	-	55.098	-
**207**	TRP	8.420	121.524	-	60.160	27.764
**208**	LEU	-	-	-	-	-
**209**	TYR	8.177	117.836	-	-	-
**210**	ALA	8.115	122.893	180.002	53.790	-
**211**	ALA	8.104	123.760	-	54.396	15.857
**212**	VAL	7.872	120.388	181.250	65.589	-
**213**	ILE	8.560	124.617	177.476	64.666	36.262
**214**	ASN	7.490	116.760	174.554	54.194	39.643
**215**	GLY	7.798	107.580	173.431	45.153	-
**216**	ASP	8.471	126.053	173.763	52.877	40.058
**217**	ARG	7.889	115.295	-	53.788	30.341
**218**	TRP	7.371	120.193	-	57.989	-
**219**	PHE	-	-	175.308	-	35.632
**220**	LEU	6.463	124.564	176.183	54.260	38.993
**221**	ASN	8.298	122.302	174.686	51.325	39.365
**222**	ARG	8.051	117.413	176.162	54.301	27.346
**223**	PHE	7.823	121.267	174.135	55.397	38.274
**224**	THR	8.075	112.933	174.107	60.785	69.624
**225**	THR	7.814	117.480	172.307	58.690	68.888
**226**	THR	8.471	111.545	175.571	59.002	70.783
**227**	LEU	9.228	124.259	178.678	57.927	39.765
**228**	ASN	8.582	115.455	177.974	55.796	37.268
**229**	ASP	7.841	119.091	179.090	56.639	39.234
**230**	PHE	8.500	120.431	176.705	61.721	37.470
**231**	ASN	8.648	118.895	177.895	54.897	36.563
**232**	LEU	7.504	120.170	179.740	57.256	40.053
**233**	VAL	6.945	119.270	177.552	64.695	29.957
**234**	ALA	8.352	121.993	180.339	54.841	15.091
**235**	MET	7.913	114.519	179.500	57.985	31.039
**236**	LYS	7.359	120.182	177.187	57.346	29.987
**237**	TYR	7.433	116.542	174.943	57.446	37.295
**238**	ASN	7.899	115.646	173.511	54.217	35.569
**239**	TYR	8.017	120.484	176.179	56.619	35.213
**240**	GLU	8.212	121.463	-	53.610	29.163

**241**	PRO	-	-	176.041	62.269	29.897
**242**	LEU	9.569	125.367	178.218	52.580	43.303
**243**	THR	10.102	119.630	175.731	58.807	70.728
**244**	GLN	8.952	119.983	177.391	57.190	26.221
**245**	ASP	7.790	117.595	178.952	56.494	38.718
**246**	HIS	7.313	119.782	177.364	57.809	31.180
**247**	VAL	7.598	118.575	179.554	66.106	29.327
**248**	ASP	8.326	120.163	181.078	56.938	38.793
**249**	ILE	8.032	120.637	177.419	63.558	36.285
**250**	LEU	7.341	120.142	176.421	54.525	41.412
**251**	GLY	7.789	108.489	-	47.642	-
**252**	PRO	-	-	179.870	65.128	30.082
**253**	LEU	7.361	118.234	-	56.459	38.706
**254**	SER	7.763	117.550	177.591	60.064	61.891
**255**	ALA	8.526	123.770	180.252	53.615	16.365
**256**	GLN	7.681	116.580	-	57.660	27.326
**257**	THR	7.375	103.708	176.083	61.601	71.140
**258**	GLY	8.059	111.969	173.656	45.074	-
**259**	ILE	7.499	120.463	-	58.137	36.709
**260**	ALA	9.445	137.408	178.178	51.712	16.531
**261**	VAL	8.906	124.884	179.551	65.620	29.804
**262**	LEU	8.894	117.239	179.385	57.145	37.695
**263**	ASP	6.961	121.388	179.216	56.196	37.824
**264**	MET	8.049	124.450	180.014	56.582	29.608
**265**	CYS	8.711	117.932	176.350	63.428	-
**266**	ALA	7.722	123.924	-	54.575	16.405
**267**	SER	7.780	117.361	175.969	62.304	60.802
**268**	LEU	8.306	124.138	176.942	56.796	38.571
**269**	LYS	8.790	119.495	177.310	59.376	30.909
**270**	GLU	7.138	116.882	178.911	58.199	27.591
**271**	LEU	7.635	120.671	180.569	56.825	40.188
**272**	LEU	8.209	119.767	177.869	56.195	38.680
**273**	GLN	7.426	113.579	177.775	57.289	28.384
**274**	ASN	8.343	114.370	176.819	52.899	39.489
**275**	GLY	7.640	109.314	-	43.830	-
**276**	MET	8.538	115.220	177.887	55.981	31.941
**277**	ASN	8.993	118.331	175.193	53.281	37.043
**278**	GLY	8.506	105.201	174.461	44.757	-
**279**	ARG	7.254	120.554	174.815	54.341	29.729
**280**	THR	7.710	106.539	175.295	58.898	71.818
**281**	ILE	8.916	121.879	-	60.960	39.191
**282**	LEU	9.720	126.778	-	54.341	36.410
**283**	GLY	8.953	103.829	172.966	44.820	-
**284**	SER	8.561	115.342	174.150	55.905	-
**285**	ALA	8.145	127.729	174.799	50.596	-
**286**	LEU	7.830	117.927	175.776	52.311	42.683
**287**	LEU	8.163	120.182	-	54.318	37.344
**288**	GLU	-	-	-	54.553	29.160
**289**	ASP	7.649	122.912	175.599	51.582	-
**290**	GLU	7.604	114.508	-	55.224	26.185
**291**	PHE	8.746	115.691	174.586	56.372	39.267
**292**	THR	8.703	110.354	-	59.072	68.053
**293**	PRO	-	-	177.983	65.427	29.773
**294**	PHE	7.661	115.852	-	59.267	36.955
**295**	ASP	8.361	122.191	179.593	56.918	39.798
**296**	VAL	7.948	120.190	177.795	66.383	29.795
**297**	VAL	7.845	120.322	179.143	65.606	-
**298**	ARG	8.438	119.928	177.980	58.573	28.303
**299**	GLN	7.559	116.463	-	57.057	28.115
**300**	CYS	8.502	114.735	-	60.608	-
**301**	SER	7.858	113.343	174.618	58.096	-
**302**	GLY	7.599	111.313	173.711	45.309	-
**303**	VAL	7.520	119.037	176.075	62.083	30.902
**304**	THR	7.902	117.062	173.573	59.878	69.821
**305**	PHE	8.048	121.084	174.691	56.514	39.040
**306**	GLN	7.801	126.233	-	56.356	29.109

Table S5: M^{pro}_{C145A}:VyLQ transferred backbone assignment (298K) achieved and validated using the 3D NOESY-TROSY experiment described in Table S6.

Res. Number	Res. Type	H	N
2	GLY	8.022	107.984
3	PHE	8.068	119.698
4	ARG	8.87	127.757
6	MET	7.572	128.142
7	ALA	8.628	128.494
8	PHE	8.863	120.314
10	SER	7.884	114.892
11	GLY	10.74	121.275
12	LYS	9.125	119.309
13	VAL	7.297	117.526
14	GLU	8.281	121.157
15	GLY	7.247	101.499
16	CYS	7.904	115.996
17	MET	7.042	118.146
18	VAL	8.785	114.541
19	GLN	8.703	120.763
20	VAL	8.928	125.88
21	THR	9.091	123.519
22	CYS	8.835	127.645
24	THR	8.767	118.072
25	THR	8.069	122.847
26	THR	8.548	123.811
27	LEU	9.099	124.028
28	ASN	7.752	115.392
29	GLY	8.472	102.716
30	LEU	8.736	122.877
31	TRP	10.271	132.003
32	LEU	9.005	127.339
33	ASP	8.91	124.948
34	ASP	8.481	123.28
35	VAL	8.065	119.266
36	VAL	9.091	124.801
37	TYR	9.377	127.334
38	CYS	8.96	118.023
40	ARG	8.157	120.256
41	HIS	7.686	119.954
42	VAL	7.028	123.298
43	ILE	7.251	109.269
44	CYS	7.516	119.465
45	THR	9.157	115.185
47	GLU	8.122	120.067
48	ASP	7.622	119.632
49	MET	7.222	112.742
50	LEU	7.342	119.56
51	ASN	7.532	112.344
53	ASN	8.418	122.633
54	TYR	8.096	120.298
55	GLU	8.684	117.567
56	ASP	7.213	119.513
57	LEU	8.247	117.794
58	LEU	8.271	119.274
59	ILE	7.437	117.638
60	ARG	6.97	117.096
61	LYS	7.707	119.222
62	SER	8.345	114.983
64	HIS	7.518	111.991
65	ASN	7.584	116.735
66	PHE	7.35	117.452
67	LEU	8.926	125.985
68	VAL	9.019	128.43
68	VAL	9.04	128.689
69	GLN	8.707	126.126
70	ALA	8.897	131.078
71	GLY	9.04	116.154
71	GLY	9.004	116.076
72	ASN	8.831	124.954
73	VAL	7.841	121.706
74	GLN	8.486	126.581
75	LEU	8.284	126.438
76	ARG	8.249	124.358
76	ARG	8.325	124.608
77	VAL	8.148	126.003
77	VAL	8.117	126.19
78	ILE	9.202	121.122
79	GLY	7.67	112.121
80	HIS	8.352	115.381
81	SER	8.306	112.936

82	MET	8.959	123.376
83	GLN	8.983	129.863
84	ASN	9.02	124.057
85	CYS	8.453	117.386
86	VAL	8.348	119.418
87	LEU	8.988	124.941
88	LYS	8.458	121.172
89	LEU	9.498	127.192
90	LYS	8.348	127.552
91	VAL	8.765	120.195
92	ASP	8.108	117.535
93	THR	6.901	114.16
94	ALA	8.588	127.87
95	ASN	7.984	121.064
97	LYS	7.489	117.99
98	THR	6.934	118.787
100	LYS	8.382	121.236
101	TYR	8.08	122.826
102	LYS	8.789	118.068
103	PHE	8.834	122.755
104	VAL	8.271	120.414
105	ARG	8.479	126.585
106	ILE	8.364	124.643
107	GLN	8.046	117.949
109	GLY	9.28	113.518
110	GLN	7.679	118.077
111	THR	7.463	110.016

112	PHE	8.365	114.433
113	SER	8.706	114.045
114	VAL	8.96	122.954
115	LEU	8.212	126.248
116	ALA	8.706	131.649
117	CYS	8.599	123.179
118	TYR	8.03	114.891
119	ASN	10.514	123.276
120	GLY	8.912	104.913
121	SER	7.927	115.472
123	SER	9.856	118.081
124	GLY	7.627	103.712
125	VAL	8.872	119.864
126	TYR	8.02	120.53
127	GLN	8.358	122.665
128	CYS	8.71	121.362
128	CYS	8.721	121.658
129	ALA	6.985	117.151
130	MET	8.349	120.339
131	ARG	9.087	129.297
133	ASN	7.007	111.399
134	PHE	8.413	111.027
135	THR	7.003	104.41
136	ILE	8.941	110.73
137	LYS	9.312	125.557
138	GLY	9.25	113.635
139	SER	6.151	112.397

140	PHE	7.732	127.509
146	GLY	9.135	113.551
147	SER	7.825	113.67
148	VAL	7.685	118.085
149	GLY	9.532	106.367
150	PHE	9.773	120.92
151	ASN	8.395	116.682
153	ASP	8.301	127.743
154	TYR	8.838	123.512
155	ASP	8.367	121.754
156	CYS	7.951	119.635
157	VAL	8.989	130.76
158	SER	8.766	124.13
159	PHE	9.188	125.695
160	CYS	8.697	117.559
161	TYR	7.47	125.481
162	MET	8.839	127.878
163	HIS	9.111	126.822
164	HIS	9.125	124.498
165	MET	8.789	125.431
166	GLU	8.973	125.928
167	LEU	8.954	128.019
170	GLY	8.452	110.57
171	VAL	6.209	108.817
172	HIS	8.962	120.916
173	ALA	9.22	120.428
174	GLY	9.588	108.845

**175**	THR	7.939	105.725
**176**	ASP	7.328	117.396
**177**	LEU	9.188	124.44
**178**	GLU	7.347	114.328
**179**	GLY	7.442	104.242
**180**	ASN	8.373	120.359
**181**	PHE	9.726	127.137
**182**	TYR	8.765	123.392
**183**	GLY	8.035	111.325
**185**	PHE	6.626	121.592
**185**	PHE	6.692	121.544
**186**	VAL	8.575	112.073
**187**	ASP	9.058	120.686
**188**	ARG	7.851	119.487
**189**	GLN	8.232	122.275
**191**	ALA	8.021	125.911
**192**	GLN	7.237	123.306
**193**	ALA	8.386	123.103
**194**	ALA	8.698	124.519
**194**	ALA	8.607	124.389
**195**	GLY	9.343	108.194
**196**	THR	8.373	117.116
**197**	ASP	8.803	128.505
**198**	THR	7.579	114.565
**199**	THR	7.958	116.899
**200**	ILE	9.8	128.829
**201**	THR	7.811	129.348

**202**	VAL	8.836	120.021
**203**	ASN	6.719	122.19
**204**	VAL	7.885	125.449
**205**	LEU	8.159	117.088
**206**	ALA	8.054	120.551
**207**	TRP	8.42	121.606
**208**	LEU	8.524	120.209
**209**	TYR	8.156	117.914
**211**	ALA	8.108	123.925
**212**	VAL	7.856	120.469
**213**	ILE	8.557	124.576
**214**	ASN	7.464	116.637
**215**	GLY	7.769	107.468
**216**	ASP	8.466	126.112
**217**	ARG	7.877	115.379
**218**	TRP	7.366	120.16
**220**	LEU	6.449	124.581
**221**	ASN	8.299	122.319
**222**	ARG	8.058	117.484
**223**	PHE	7.826	121.258
**224**	THR	8.068	112.998
**225**	THR	7.797	117.495
**226**	THR	8.474	111.616
**227**	LEU	9.229	124.343
**228**	ASN	8.581	115.53
**229**	ASP	7.841	119.127
**230**	PHE	8.485	120.41

**231**	ASN	8.637	118.924
**232**	LEU	7.515	120.174
**233**	VAL	6.935	119.184
**234**	ALA	8.347	121.99
**235**	MET	7.931	114.626
**236**	LYS	7.352	120.205
**237**	TYR	7.436	116.564
**238**	ASN	7.897	115.7
**239**	TYR	7.977	120.396
**240**	GLU	8.231	121.567
**242**	LEU	9.547	125.469
**243**	THR	10.139	119.851
**244**	GLN	8.953	120.022
**245**	ASP	7.765	117.574
**246**	HIS	7.305	119.847
**247**	VAL	7.596	118.644
**248**	ASP	8.334	120.21
**249**	ILE	8.031	120.717
**250**	LEU	7.335	120.273
**251**	GLY	7.774	108.455
**253**	LEU	7.395	118.401
**254**	SER	7.755	117.555
**255**	ALA	8.52	123.821
**256**	GLN	7.7	116.576
**257**	THR	7.369	103.752
**258**	GLY	8.047	111.969
**259**	ILE	7.489	120.491

**260**	ALA	9.445	136.390
**261**	VAL	8.896	124.969
**262**	LEU	8.898	117.276
**263**	ASP	6.957	121.448
**264**	MET	8.046	124.559
**265**	CYS	8.707	117.948
**266**	ALA	7.715	123.985
**267**	SER	7.778	117.313
**268**	LEU	8.301	124.179
**269**	LYS	8.788	119.551
**270**	GLU	7.138	116.924
**271**	LEU	7.629	120.772
**272**	LEU	8.199	119.727
**273**	GLN	7.409	113.493
**274**	ASN	8.33	114.343
**275**	GLY	7.652	109.382
**276**	MET	8.539	115.226
**277**	ASN	8.996	118.376
**278**	GLY	8.504	105.2
**279**	ARG	7.248	120.552
**280**	THR	7.705	106.584
**281**	ILE	8.917	121.93
**282**	LEU	9.731	126.854
**283**	GLY	8.95	103.844
**284**	SER	8.561	115.292
**285**	ALA	8.143	127.752
**286**	LEU	7.824	117.958

**287**	LEU	8.162	121.019
**289**	ASP	7.637	122.963
**290**	GLU	7.682	114.695
**292**	THR	8.713	110.405
**294**	PHE	7.621	115.875
**295**	ASP	8.345	122.282
**296**	VAL	7.936	120.104
**297**	VAL	7.762	119.962
**298**	ARG	8.437	120.111
**299**	GLN	7.622	116.659
**300**	CYS	8.54	115.031
**302**	GLY	7.501	111.438
**303**	VAL	7.436	119.183
**304**	THR	8.088	118.405
**304**	THR	7.89	117.335
**304**	THR	7.86	117.211
**305**	PHE	8.052	120.721
**306**	GLN	7.776	126.233
**306**	GLN	7.76	126.452

Table S6: NMR acquisition parameters for NOE-based experiments. Acquisition parameters for the SILLY-TROSY spectra, where values are doubled due to interleaving, are denoted with an asterisk.

Experiment	NOESY-TROSY (VyLQ)			SILLY - NOESY - TROSY		NOESY-TROSY (SAVLQSGFRK)		
Magnetic field strength	21.1 T (900 MHz)			18.8 T (800 MHz)		18.8 T (800 MHz)		
Dimension	F3	F2	F1	F2	F1	F3	F2	F1
Nucleus Observed	1H	15N	1H	1H	15N	1H	15N	1H
Quadrature detection	Direct	Echo-AntiEcho	States-TPPI	Direct	Echo-AntiEcho	Direct	Echo-AntiEcho	States-TPPI
Topspin TD size	2048	290	256	2284	400 (800)*	3072	308	288
No. of increment (i.e. complex pairs)	1024	145	128	1142	200 (400)*	1536	154	144
Spectral width (Hz)	14705.9	2924.0	10803.3	14423.1	2777.8	12820.5	2564.1	9615.4
Acquisition time (msec)	69.6	49.6	11.8	79.2	72	119.8	60	15
SMILE extended acquisition time (msec)	N/A	74.4	16.8	N/A	N/A	N/A	90	22.5
NOESY mixing period	200ms			70-500ms		200ms		
Topspin NUS sampling random seed	2102031620			N/A		54321		
NUS sampling percentage (effective)	6.47% (3.06%)			N/A		40.0% (17.85%)		
Total No. of FID (NUS points) recorded	4,800 (1200)			6400 (N/A)		35,480 (8,870)		
Interscan delay (d1)	1.8 s			1.8 s		1.7 s		
Total experiment acquisition time	10.5h			3.4h		3d 3h		

Author Contributions

AJR carried out the NMR measurements, analyzed the data and drafted the original manuscript with support from JY; AB directed the study and edited the manuscript.

References

References

[1] M. Cai, Y. Huang, R. Yang, R. Craigie, G. M. Clore, *J. Biomol. NMR* **2016**, *66*, 85-91.
[2] aM. A. C. Reed, A. M. Hounslow, K. H. Sze, I. G. Barsukov, L. L. P. Hosszu, A. R. Clarke, C. J. Craven, J. P. Waltho, *J. Mol. Biol.* **2003**, *330*, 1189-1201; bK. H. Gardner, L. E. Kay, *J. Am. Chem. Soc.* **1997**, *119*, 7599-7600.
[3] A. I. Iorgu, N. J. Baxter, M. J. Cliff, J. P. Waltho, S. Hay, N. S. Scrutton, *Biomol NMR Assign* **2018**, *12*, 79-83.
[4] H. P. Chang, C. Y. Chou, G. G. Chang, *Biophys. J.* **2007**, *92*, 1374-1383.
[5] M. Salzmann, G. Wider, K. Pervushin, H. Senn, K. Wüthrich, *J. Am. Chem. Soc.* **1999**, *121*, 844-848.
[6] F. Delaglio, S. Grzesiek, G. W. Vuister, G. Zhu, J. Pfeifer, A. Bax, *J. Biomol. NMR* **1995**, *6*, 277-293.
[7] aT. D. Goddard, D. G. Kneller, *University of California, San Francisco*; bW. Lee, M. Tonelli, J. L. Markley, *Bioinformatics* **2015**, *31*, 1325-1327.
[8] W. Lee, M. Rahimi, Y. Lee, A. Chiu, *Bioinformatics* **2021**.
[9] E. Schmidt, P. Guntert, *J. Am. Chem. Soc.* **2012**, *134*, 12817-12829.
[10] Y. Shen, A. Bax, *J. Biomol. NMR* **2010**, *48*, 13-22.
[11] S. Zhang, N. Zhong, X. Ren, C. Jin, B. Xia, *Biomol NMR Assign* **2011**, *5*, 143-145.
[12] aC. R. Harris, K. J. Millman, S. J. van der Walt, R. Gommers, P. Virtanen, D. Cournapeau, E. Wieser, J. Taylor, S. Berg, N. J. Smith, R. Kern, M. Picus, S. Hoyer, M. H. van Kerkwijk, M. Brett, A. Haldane, J. F. Del Rio, M. Wiebe, P. Peterson, P. Gerard-Marchant, K. Sheppard, T. Reddy, W. Weckesser, H. Abbasi, C. Gohlke, T. E. Oliphant, *Nature* **2020**, *585*, 357-362; bP. Virtanen, R. Gommers, T. E. Oliphant, M. Haberland, T. Reddy, D. Cournapeau, E. Burovski, P. Peterson, W. Weckesser, J. Bright, S. J. van der Walt, M. Brett, J. Wilson, K. J. Millman, N. Mayorov, A. R. J. Nelson, E. Jones, R. Kern, E. Larson, C. J. Carey, I. Polat, Y. Feng, E. W. Moore, J. VanderPlas, D. Laxalde, J. Perktold, R. Cimrman, I. Henriksen, E. A. Quintero, C. R. Harris, A. M. Archibald, A. H. Ribeiro, F. Pedregosa, P. van Mulbregt, C. SciPy, *Nat. Methods* **2020**, *17*, 261-272; cJ. D. Hunter, *Comput. Sci. Eng.* **2007**, *9*, 90-95.
[13] J. J. Helmus, C. P. Jaroniec, *J. Biomol. NMR* **2013**, *55*, 355-367.
[14] J. Jumper, R. Evans, A. Pritzel, T. Green, M. Figurnov, O. Ronneberger, K. Tunyasuvunakool, R. Bates, A. Zidek, A. Potapenko, A. Bridgland, C. Meyer, S. A. A. Kohl, A. J. Ballard, A. Cowie, B. Romera-Paredes, S. Nikolov, R. Jain, J. Adler, T. Back, S. Petersen, D. Reiman, E. Clancy, M. Zielinski, M. Steinegger, M. Pacholska, T. Berghammer, S. Bodenstein, D. Silver, O. Vinyals, A. W. Senior, K. Kavukcuoglu, P. Kohli, D. Hassabis, *Nature* **2021**, *596*, 583-589.
[15] A. J. Robertson, J. M. Courtney, Y. Shen, J. Ying, A. Bax, *J. Am. Chem. Soc.* **2021**, *143*, 19306-19310.
[16] R. Evans, M. O'Neill, A. Pritzel, N. Antropova, A. Senior, T. Green, A. Žídek, R. Bates, S. Blackwell, J. Yim, O. Ronneberger, S. Bodenstein, M. Zielinski, A. Bridgland, A. Potapenko, A. Cowie, K. Tunyasuvunakool, R. Jain, E. Clancy, P. Kohli, J. Jumper, D. Hassabis, *bioRxiv* **2021**.
[17] D. A. Case, T. E. Cheatham, 3rd, T. Darden, H. Gohlke, R. Luo, K. M. Merz, Jr., A. Onufriev, C. Simmerling, B. Wang, R. J. Woods, *J. Comput. Chem.* **2005**, *26*, 1668-1688.
[18] M. Zweckstetter, *Protein Sci.* **2021**.
[19] J. M. Word, S. C. Lovell, J. S. Richardson, D. C. Richardson, *J. Mol. Biol.* **1999**, *285*, 1735-1747.
[20] F. Delaglio, S. Grzesiek, G. W. Vuister, G. Zhu, J. Pfeifer, A. Bax, *J. Biomol. NMR* **1995**, *6*, 277-293.
[21] M. P. Williamson, *Prog. Nucl. Magn. Reson. Spectrosc.* **2013**, *73*, 1-16.
[22] F. X. Cantrelle, E. Boll, L. Brier, D. Moschidi, S. Belouzard, V. Landry, F. Leroux, F. Dewitte, I. Landrieu, J. Dubuisson, B. Deprez, J. Charton, X. Hanoulle, *Angew. Chem. Int. Ed. Engl.* **2021**.
[23] S. Chen, F. Jonas, C. Shen, R. Hilgenfeld, *Protein Cell* **2010**, *1*, 59-74.
[24] J. F. Ying, F. Delaglio, D. A. Torchia, A. Bax, *J. Biomol. NMR* **2017**, *68*, 101-118.
[25] aA. J. Shaka, J. Keer, R. Freeman, *J. Mag. Res.,* **1983**, *53*, 313-340.
[26] K. Kazimierczuk, P. Kasprzak, P. S. Georgoulia, I. Matecko-Burmann, B. M. Burmann, L. Isaksson, E. Gustavsson, S. Westenhoff, V. Y. Orekhov, *Chem. Comm.* **2020**, *56*, 14585-14588.
